# Therapeutic Strategies to Modulate Gut Microbial Health: Approaches for Chronic Metabolic Disorder Management

**DOI:** 10.3390/metabo15020127

**Published:** 2025-02-13

**Authors:** Mariangela Rondanelli, Sara Borromeo, Alessandro Cavioni, Clara Gasparri, Ilaria Gattone, Elisa Genovese, Alessandro Lazzarotti, Leonardo Minonne, Alessia Moroni, Zaira Patelli, Claudia Razza, Claudia Sivieri, Eugenio Marzio Valentini, Gaetan Claude Barrile

**Affiliations:** 1 Department of Public Health, Experimental and Forensic Medicine, University of Pavia, 27100 Pavia, Italy; mariangela.rondanelli@unipv.it; 2 Endocrinology and Nutrition Unit, Azienda di Servizi alla Persona “Istituto Santa Margherita”, University of Pavia, 27100 Pavia, Italy; sara.borromeo01@universitadipavia.it (S.B.); alessandro.cavioni01@universitadipavia.it (A.C.); clara.gasparri01@universitadipavia.it (C.G.); ilaria.gattone01@universitadipavia.it (I.G.); elisa.genovese01@universitadipavia.it (E.G.); alessandro.lazzarotti01@universitadipavia.it (A.L.); leonardo.minonne01@universitadipavia.it (L.M.); alessia.moroni02@universitadipavia.it (A.M.); zaira.patelli01@universitadipavia.it (Z.P.); claudia.razza01@universitadipavia.it (C.R.); claudia.sivieri01@universitadipavia.it (C.S.); eugeniomarzio.valentini01@universitadipavia.it (E.M.V.)

**Keywords:** intestinal microbiota, diabetes, prediabetes, obesity, metabolic syndrome, sarcopenia, dyslipidemia, hyperhomocysteinemia, non-alcoholic fatty liver disease, probiotics, prebiotics, dietary supplements, dysbiosis, eubiosis

## Abstract

Numerous recent studies have suggested that the composition of the intestinal microbiota can trigger metabolic disorders, such as diabetes, prediabetes, obesity, metabolic syndrome, sarcopenia, dyslipidemia, hyperhomocysteinemia, and non-alcoholic fatty liver disease. Since then, considerable effort has been made to understand the link between the composition of intestinal microbiota and metabolic disorders, as well as the role of probiotics in the modulation of the intestinal microbiota. The aim of this review was to summarize the reviews and individual articles on the state of the art regarding ideal therapy with probiotics and prebiotics in order to obtain the reversion of dysbiosis (alteration in microbiota) to eubiosis during metabolic diseases, such as diabetes, prediabetes, obesity, hyperhomocysteinemia, dyslipidemia, sarcopenia, and non-alcoholic fatty liver diseases. This review includes 245 eligible studies. In conclusion, a condition of dysbiosis, or in general, alteration of the intestinal microbiota, could be implicated in the development of metabolic disorders through different mechanisms, mainly linked to the release of pro-inflammatory factors. Several studies have already demonstrated the potential of using probiotics and prebiotics in the treatment of this condition, detecting significant improvements in the specific symptoms of metabolic diseases. These findings reinforce the hypothesis that a condition of dysbiosis can lead to a generalized inflammatory picture with negative consequences on different organs and systems. Moreover, this review confirms that the beneficial effects of probiotics on metabolic diseases are promising, but more research is needed to determine the optimal probiotic strains, doses, and administration forms for specific metabolic conditions.

## 1. Introduction

The intestinal microbiota is not only a simple component of the gastrointestinal tract but appears to have a specific role in the pathogenesis of many metabolic diseases, such as obesity, prediabetes [1], diabetes [2], and non-alcoholic fatty liver disease [3].

The factors that contribute to the diversity and interindividual variability of the microbiota are many and are related to genetics, nutrition and lifestyle, some drugs, such as antibiotics, and changes in the composition of the diet [4].

Dietary intake significantly influences the composition of the intestinal microbiota, which in turn affects various organs throughout the body, including the brain, kidneys, heart, and lungs. This relationship is complex and involves multiple mechanisms that underscore the gut microbiota’s role as a critical component of human health [5].

Considering the mechanisms of action that affect multiple organs, these include alteration of endocrine functions, neurotransmitter production, immune modulation, and metabolic regulation. Regarding endocrine functions, the gut microbiota functions similarly to an endocrine organ by producing metabolites that can influence hormone secretion and systemic metabolism. This includes the production of molecules that regulate appetite and energy balance, potentially affecting conditions like obesity and diabetes [6].

Regarding neurotransmitter production, gut bacteria can produce neurotransmitters such as serotonin and gamma-aminobutyric acid (GABA), which may influence mood and cognitive functions through the gut–brain axis. This highlights how dietary choices can indirectly affect brain health via microbial activity [5].

Considering immune modulation, the gut microbiota plays a crucial role in modulating immune responses. An imbalance in gut bacteria (dysbiosis) can lead to increased intestinal permeability (“leaky gut”), allowing harmful substances to enter circulation and trigger systemic inflammation. This has been linked to chronic diseases affecting various organs, including cardiovascular diseases [7].

Regarding metabolic regulation, gut microbes are involved in the metabolism of bile acids and other metabolites that can influence lipid metabolism and cardiovascular health. For instance, certain bacterial metabolites can lower levels of trimethylamine-N-oxide (TMAO), a compound associated with increased cardiovascular risk.

Various dietary patterns, including plant-based, Mediterranean, and Western diets, have been shown to produce distinct effects on gut microbiota composition and functionality [8].

Considering key dietary influences, plant-based and mediterranean diets are fiber-rich diets and are consistently linked with positive changes in gut microbiota. These diets enhance SCFA production and microbial diversity while lowering TMAO levels. They are associated with lower risks of non-communicable diseases (NCDs) due to their anti-inflammatory properties and ability to maintain gut homeostasis [9].

These fiber-rich diets promote the growth of beneficial bacteria that produce short-chain fatty acids (SCFAs), which are important for gut health. High fiber intake is associated with increased microbial diversity and reduced levels of harmful bacteria [8,10].

A rapid switch from a fiber-rich diet to a meat-based diet can lead to significant changes in gut microbiota diversity within just 24 h [10].

Short-term dietary changes can induce rapid shifts in gut microbiota composition; however, long-term dietary habits have a more profound impact on establishing a stable “core” microbiome [11]. 

Research indicates that habitual dietary patterns significantly influence the resilience and functionality of gut microbiota over time [11].

Conversely, Western diets, which are typically high in fats and low in fiber, lead to dysbiosis—an imbalance in gut microbiota characterized by reduced diversity and increased pathogenic bacteria [8,10].

This dietary pattern is linked to higher levels of trimethylamine-N-oxide (TMAO), a compound associated with negative health outcomes.

Diet affects gut microbiota through several mechanisms, such as fermentation (non-digestible carbohydrates (dietary fibers) are fermented by gut bacteria, producing SCFAs that provide energy for colon cells and modulate immune responses [10]), microbial metabolites (the metabolites produced by gut bacteria influence host metabolism, immune function, and even neurological health [9]), and dysbiosis (changes in diet can disrupt the balance of gut bacteria, leading to dysbiosis, which is implicated in various health disorders including obesity, diabetes, and inflammatory bowel diseases [8,9,10]).

In conclusion, the composition of the intestinal microbiota is heavily influenced by dietary intake, with significant implications for health. Diets rich in fiber and plant-based foods promote beneficial microbial communities that support overall health, while Western dietary patterns contribute to dysbiosis and increased disease risk. Understanding these relationships may pave the way for personalized dietary interventions aimed at improving gut health and preventing chronic diseases.

So, observational studies over the past two decades have linked the gut microbiota to the development of various metabolic disorders, including diabetes, prediabetes, obesity, metabolic syndrome, sarcopenia, dyslipidemia, hyperhomocysteinemia, and non-alcoholic fatty liver disease [12].

This review discusses the current knowledge on how gut microbiota and derived microbial compounds may link to the metabolism of the healthy host or to the pathogenesis of common metabolic diseases. Key findings on this topic include the following: metabolic pathologies are associated with an altered gut microbiota composition; specific changes in gut microbial species and functions have been implicated in the pathogenesis of metabolic diseases; transplantation of gut microbiota from lean to obese individuals can improve insulin sensitivity in the recipients, demonstrating a causal link.

Various mechanisms link gut microbiota to metabolic inflammation. The gut microbiota can contribute to metabolic inflammation and insulin resistance through several mechanisms; for example, microbial metabolites like short-chain fatty acids can modulate host metabolism and immunity; bacterial components can promote inflammation by stimulating immune cells and cytokine production in metabolic tissues; and gut microbiota dysbiosis can impair intestinal barrier function, leading to increased translocation of microbial products that drive inflammation [12].

Another interesting new metabolic pathology is sarcopenia. Sarcopenia, characterized by the progressive loss of skeletal muscle mass and function, is increasingly recognized as a metabolic disease, particularly in the context of metabolic syndrome (MetS) [13].

Sarcopenia is linked to various metabolic dysfunctions, including insulin resistance (IR) and chronic inflammation, which are hallmark features of MetS. MetS itself is a cluster of conditions that increase the risk of heart disease, stroke, and diabetes, characterized by obesity, hypertension, dyslipidemia, and elevated blood glucose levels. The relationship between sarcopenia and MetS is complex. Skeletal muscle plays a crucial role in glucose metabolism; thus, its loss can lead to increased IR, further contributing to the metabolic disturbances seen in MetS. Factors such as oxidative stress, inflammatory cytokines, and mitochondrial dysfunction are implicated in both conditions, suggesting a bidirectional relationship where each condition can exacerbate the other [14].

Another interesting study, in which 13,620 participants were enrolled, has demonstrated that sarcopenia, by BIA, is independently associated with the risk of MetS and has a dose–response relationship [15].

In conclusion, the gut microbiota influences host metabolism through several mechanisms, such as the production of metabolites, the regulation of satiety, the modulation of inflammation, and the regulation of gut barrier function.

Considering the production of metabolites, the gut microbiota produces various metabolites, including short-chain fatty acids, trimethylamine-N-oxide (TMAO), and bile acids, which can modulate glucose and lipid homeostasis, energy production, and inflammation [12,16].

Butyrate, for example, is known to enhance insulin sensitivity and has protective effects against obesity-related inflammation [17].

Alterations in bile acid composition have been linked to metabolic disorders, such as obesity and type 2 diabetes [18,19].

Regarding the regulation of satiety, gut microbiota-derived metabolites can regulate appetite and satiety by affecting the production of hormones like glucagon-like peptide-1 (GLP-1) and peptide YY (PYY) [20]. Regarding the modulation of inflammation, an altered gut microbiota can promote low-grade inflammation, which is a key feature of metabolic diseases [21]. As far as regulation of gut barrier function is concerned, changes in gut microbiota composition can impair the intestinal barrier, leading to increased permeability and translocation of bacterial products like lipopolysaccharide (LPS), contributing to systemic inflammation and metabolic disorders. Strategies to modulate the gut microbiota, such as prebiotics, probiotics, symbiotics, and fecal microbiota transplantation, have shown potential in optimizing metabolic health and preventing or treating metabolic diseases. They can improve gut barrier function, reduce inflammation, and enhance the production of beneficial metabolites like short-chain fatty acids (SCFAs) [22].

Moreover, beyond short-chain fatty acids, trimethylamine-N-oxide (TMAO), and bile acids, other key metabolites from the gut microbiota include branched-chain amino acids (BCAAs) and indole derivatives. BCAAs are derived from protein metabolism and can be metabolized by gut microbiota into various bioactive compounds. Elevated levels of BCAAs have been associated with insulin resistance and metabolic syndrome [18,19].

Indoles are produced from the metabolism of tryptophan by gut bacteria. These metabolites can influence serotonin production and have been linked to mood regulation and metabolic functions [19,23].

These metabolites produced by gut microbiota exert their effects through several mechanisms, such as energy harvesting, immune modulation, and hormonal modulation. Regarding energy harvesting, the microbiota enhances the host’s ability to extract energy from complex carbohydrates that are indigestible by human enzymes, leading to increased caloric intake and potential weight gain [24].

Considering immune modulation, SCFAs can promote regulatory T-cell differentiation, thereby modulating immune responses and reducing inflammation associated with metabolic diseases [17,25].

Regarding hormonal regulation, bile acids interact with FXR to regulate the expression of genes involved in lipid metabolism, glucose homeostasis, and energy expenditure. This interaction can influence insulin sensitivity and fat storage [17,18].

Probiotics, prebiotics, and symbiotics have emerged as potential positive modulators of gut microbiota and immunity [26].

Probiotics may also influence host metabolism, energy homeostasis, and glucose and lipid metabolism through various mechanisms [27].

Also, symbiotics, as a mixture of live microorganisms (e.g., probiotics) and substrates (e.g., prebiotics) that are selectively utilized by host microorganisms, have emerged as potential modulators of gut microbiota and immunity [26].

Recent studies highlight their potential benefits in modulating immune responses and improving gut health, which is closely linked to overall immunity, through various mechanisms, such as the modulation of gut microbiota, cytokine production, and reductions in inflammatory markers. Regarding the modulation of gut microbiota, synbiotics can alter the composition of the gut microbiota, promoting beneficial bacteria such as *Bifidobacterium* and *Lactobacillus*. This shift can enhance the production of short-chain fatty acids (SCFAs) and other metabolites that positively influence immune function [28].

As several studies have already demonstrated the potential of using probiotics and prebiotics in the treatment of metabolic disorders, detecting significant improvements in the specific symptoms of metabolic diseases, the aim of this review was the evaluation of the state of the art regarding ideal therapy with probiotics and prebiotics in order to obtain the reversion of dysbiosis (alteration in microbiota) to eubiosis during metabolic diseases, such as metabolic syndrome, diabetes, prediabetes, obesity, hyperhomocysteinemia, dyslipidemia, sarcopenia, and non-alcoholic fatty liver disease.

## 2. Materials and Methods

**Figure 1:**
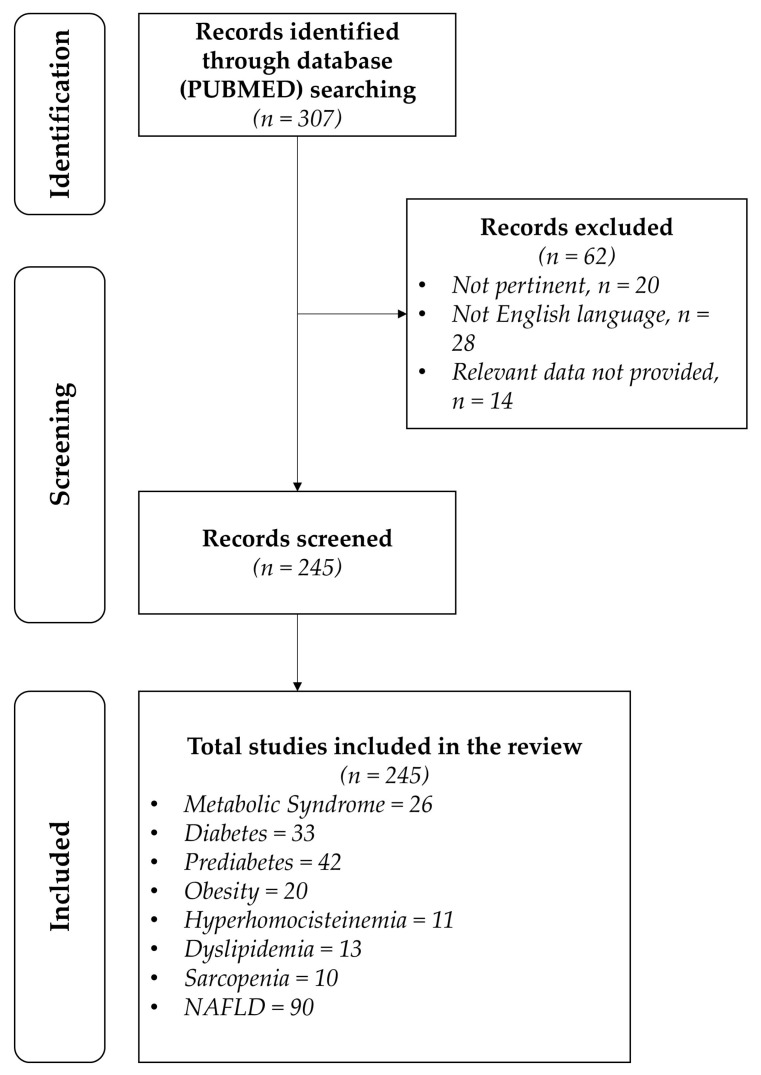
Flow chart of the eligible studies.

This narrative review was performed following these steps [29,30]:

(1) Configuration of a working group: Three operators skilled in clinical nutrition were gathered (one acting as a methodological operator and two participating as clinical operators).
(2) Formulation of the revision question on the basis of considerations made in the abstract: “the state of the art regarding ideal therapy with probiotics and prebiotics in order to obtain the reversion of dysbiosis (alteration in microbiota) to eubiosis during metabolic diseases, such as metabolic syndrome, diabetes, prediabetes, obesity, hyperhomocysteinemia, dyslipidemia, sarcopenia, and non-alcoholic fatty liver disease”.
(3) Identification of relevant studies: A research strategy was planned on PubMed (Public MEDLINE run by the National Center of Biotechnology Information (NCBI) of the National Library of Medicine of Bethesda (Bethesda, MD, USA)) as follows: (a) definition of the keywords (metabolic syndrome, diabetes, prediabetes, obesity, hyperhomocysteinemia, dyslipidemia, sarcopenia, non-alcoholic fatty liver disease, microbiota, probiotics, prebiotics, and dietary supplements), allowing the definition of the interest field of the documents to be searched, grouped in inverted commas (“. . .”), and used separately or in combination; (b) use of the Boolean (a data type with only two possible values: true or false) AND operator, which allows the establishment of logical relations among concepts; (c) research modalities: advanced search; (d) limits (time limits: papers published in the last 20 years; humans; languages: English); (e) manual search performed by senior researchers experienced in clinical nutrition through the revision of reviews and individual articles on the state of the art regarding ideal therapy with probiotics and prebiotics in order to obtain the reversion of dysbiosis (alteration in microbiota) to eubiosis during metabolic diseases, such as metabolic syndrome, diabetes, prediabetes, obesity, hyperhomocysteinemia, dyslipidemia, sarcopenia, and non-alcoholic fatty liver disease published in journals qualified in the Index Medicus.
(4) Analysis and presentation of the outcomes: The data extrapolated from the “revised studies” were collocated in tables, particularly, for each study specified, the author and year of publication and the study characteristics; for each topic, we built three types of tables depending on the type of study: tables with reviews and meta-analyses, tables with observational human studies, and tables with interventional human studies. In the tables (obviously except for reviews and meta-analyses), only studies on humans are reported, while, in the text, in vitro studies and studies on animal models are also cited, if useful to explain some mechanisms of action. Moreover, in tables, for all studies, the level of evidence has been added [31].
(5) The analysis was carried out in the form of a narrative review of the reports. At the beginning of each section, the keywords considered and the kind of studies chosen have been reported. We evaluated, as suitable for the narrative review, the studies of any design that considered the state of the art of ideal therapy with probiotics and prebiotics in order to obtain the reversion of dysbiosis (alteration in microbiota) to eubiosis during metabolic diseases, such as metabolic syndrome, diabetes, prediabetes, obesity, hyperhomocysteinemia, dyslipidemia, sarcopenia, and non-alcoholic fatty liver disease. Figure 1 shows the eligible studies.

## 3. Results

### 3.1. Metabolic Syndrome

This research was conducted based on the keywords “gut microbiota” OR “pre-biotics” OR “probiotics” OR “synbiotics” AND “metabolic syndrome” OR “X Syndrome” OR “metabolic disorder syndrome”.

A total of 26 articles were sourced: 7 systematic reviews, 5 meta-analyses, 7 randomized controlled trials (RCTs), 1 observational study, 2 pilot studies, 2 narrative reviews, and 2 interventional studies.

Table 1A,B include studies that assessed the connection between gut microbiota and metabolic syndrome; Table 1C,B include studies focused on the impact of probiotics, prebiotics, and synbiotics on metabolic syndrome alongside the strength of the evidence.

### 3.2. Diabetes

The keywords used in this research were “microbiota” OR “probiotics” OR “prebiotics” AND “diabetes” OR “glycemic control” OR “metabolic health”.

A total of 33 studies were sourced, including 8 meta-analyses, 8 randomized controlled trials (RCTs), 5 observational or cohort studies, 4 animal studies, 1 in vitro study, and 7 systematic reviews.

Table 2A,B include studies that assessed the connection between gut microbiota and diabetes, focusing on the role of gut microbiota in influencing metabolic markers such as blood glucose, HbA1c, and insulin resistance. Table 2C,D present studies evaluating the effects of probiotics, prebiotics, and synbiotics on metabolic outcomes, including blood glucose levels, HbA1c, and other key metabolic parameters, along with the strength of evidence supporting these interventions.

### 3.3. Prediabetes

This research was conducted based on the keywords “gut microbiota” OR “pre-biotics” OR “probiotics” OR “synbiotics” AND “prediabetes” OR “blood glucose” OR “diabetes mellitus” OR “metabolic syndrome”.

Forty-two articles were sourced: three narrative reviews, one systematic review of observational studies, three systematic reviews of randomized controlled trials, two systematic reviews and meta-analyses, sixteen clinical trials (six observational studies, one case–control study and nine randomized controlled trials), two in vitro studies, and twenty-six studies on animal models.

Table 3A,B (clinical trials and reviews) show the studies that evaluated the relationship between microbiota, diet, and prediabetes with their strength of evidence. Table 3C,D (clinical trials and reviews) show the studies that evaluated the relationship between the use of prebiotics, probiotics, and synbiotics in prediabetes (and predisposing conditions) with their strength of evidence.

### 3.4. Obesity

This research was conducted based on the keywords “microbiota” OR “probiotics” OR “prebiotics” AND “obesity” OR “weight management” OR “metabolic health”.

A total of twenty articles were sourced: four systematic reviews, four meta-analysis, ten randomized controlled trials (RCTs), one observational study, and one interventional study.

Table 4A,B include studies that assessed the connection between gut microbiota and obesity; Table 4B,C include studies focused on the impact of probiotics, prebiotics, and synbiotics on body weight, fat mass, and metabolic markers, alongside the strength of evidence.

### 3.5. Hyperhomocisteinemia

The keywords used in this research were “hyperhomocysteinemia” OR “high blood homocysteine” OR “high metionine and high fat diet” AND “microbiota” OR “intestinal microbiota”.

Eleven articles were sourced: three narrative reviews, one systematic review, five controlled clinical trials, one pilot study controlled clinical trial, and one randomized double-blind placebo-controlled study.

Table 5A includes studies that assessed the connection between hyperhomocysteinemia and microbiota, alongside the strength of evidence. Table 5B shows the studies that evaluated the relationship between the use of prebiotics, probiotics, and synbiotics and hyperhomocysteinemia with their strength of evidence.

### 3.6. Dyslipidemia

The keywords used in this research were “microbiota” OR “probiotics” OR “prebiotics” AND “dyslipidemia” OR “lipid metabolism” OR “cardiovascular health”.

A total of thirteen articles were sourced, including two meta-analyses, four systematic reviews, three randomized controlled trials (RCTs), and four clinical studies.

Table 6A,B show the studies (clinical trials and reviews) that evaluated the relationship between microbiota, diet, and dyslipidemia with their strength of evidence. Table 6C,D show the studies (clinical trials and reviews) that evaluated the relationship between the use of prebiotics, probiotics, and synbiotics and dyslipidemia with their strength of evidence.

### 3.7. Sarcopenia

The keywords used in this search were “sarcopenia” AND “muscle mass” AND “gut microbiota” OR “probiotics” OR “prebiotics”.

Ten articles were selected: one narrative review, one systematic review, one longitudinal study, two case–control studies, one cross-sectional study, one uncontrolled experimental study, and three double-blind randomized controlled trials.

Table 7A summarizes the evidence on the studies that investigated the composition of the microbiota in sarcopenic patients, showing reductions in microbial diversity, deficiencies in key species such as *Bifidobacterium longum* and *Prevotella copri*, and increases in pro-inflammatory bacteria. Table 7B focuses on studies evaluating the effects of interventions with probiotics or prebiotics in improving muscle strength and body mass in subjects with sarcopenia.

### 3.8. NAFLD

This research was conducted based on the keywords “gut microbiota” OR “pre-biotics” OR “probiotics” OR “synbiotics” AND “NAFLD” OR “steatosis” OR “steato inflammation” OR “metabolic syndrome”.

Ninety articles were sourced: two systematic reviews, two reviews related to animal model studies, one guideline, one meta-analysis, one practice guidance, four systematic reviews and meta-analyses, twenty-three narrative reviews, thirteen clinical trials (four observational studies, one comparative study, two cross-sectional studies, one study cohort, four randomized controlled trials, one four-arm parallel, randomized, and single blind trial, two comments, one book, thirteen in vitro studies, and twenty-seven studies of animal models.

Table 8A,B show the studies (clinical trials and reviews) that evaluated the relationship between microbiota, diet, and NAFLD with their strength of evidence. Table 8C,D show the studies (clinical trials and reviews) that evaluated the relationship between the use of prebiotics, probiotics, and synbiotics and NAFLD with their strength of evidence.

## 4. Discussion

### 4.1. Metabolic Syndrome

Metabolic syndrome (MetS), which is brought on by urbanization, excess calorie intake, rising obesity rates, and sedentary lifestyles, is a significant and growing global public health and clinical concern.

The World Health Organization defines metabolic syndrome as a pathologic state characterized by abdominal obesity, insulin resistance, hypertension, and dyslipidemia [161].

Over five to ten years, MetS increases the risk of type 2 diabetes mellitus (T2DM) by five times and the risk of cardiovascular disease (CVD) by two times [162]. In addition, regardless of a prior history of cardiovascular events, patients with the MetS have a two- to four-fold greater risk of stroke, a three- to four-fold increased risk of myocardial infarction (MI), and a two-fold increased chance of dying from such an event [163].

According to the 2015 review by Portela-Cidade J.P. et al. [32], microbiome dysbiosis is prevalent in metabolic syndrome: in patients with MetS, there is an increased presence of Firmicutes bacteria and a lower presence of Bacteroidetes than in the general population. The authors also theorize about the correlation of unbalanced microbiota and increased expression of Toll-like receptor, a well-known inflammatory receptor, that could lead to a pro-inflammatory unbalanced state. In particular, studies on TLR2-knockout mice placed emphasis on the fact that TLR2 inexpression is associated with better glucose tolerance and insulin sensitivity (as well as reduced levels of leptin and tumor necrosis factor) compared to normal mice in a germ-free environment and whilst intaking a high-fat diet, but in a standard (non-germ-free) environment, TLR2-knockout mice have higher levels of Bacteroidetes and firmicutes in gut microbiota than the control group and have increased lipopolysaccharide absorption, glucose intolerance, insulin resistance, and obesity. This particular evidence suggests that TLR expression and activity is deeply connected to metabolic outcomes and to microbiota composition.

Fecal microbiota transplantation (FMT) is the process of transferring stool from a healthy donor to a recipient who likely has an unhealthy gut bacterial composition, causing disease, with the objective of re-establishing a healthy balance in the microbiome. The reasoning behind this practice is based on the fact that various diseases have been associated with dysbiosis [164], a condition where the gut microbiome compared to healthy individuals is less diverse, contains more inflammatory microbes, and produces fewer short-chain fatty acids and other beneficial metabolites [165].

Also known as stool transplantation or fecal bacteriotherapy, FMT has garnered increasing interest in public discourse and scientific studies. One of the main applications of FMT has been in the treatment of resistant Clostridium difficile infection (rCDI) [166].

Given that the primary objective of FMT is to re-establish the typical bacterial community in an imbalanced colon, researchers have analyzed the gut microbiome in individuals with CDI both pre- and post-FMT, detecting a noticeable change in the bacterial composition in the recipient’s gut after FMT, with a result that resembles the microbiota of the donor’s stool. Genetic sequencing of stool samples revealed higher levels of Firmicutes and Bacteroidetes and reduced amounts of Proteobacteria and Actinobacteria after FMT, indicating swift integration of the donor’s bacteria [167]. The donor and the recipient may or may not be related as this distinction does not seem to be clinically significant [168]. Screening of the donor has yet to be standardized, although researchers commonly suggest screening for the most common parenteral infectious diseases. Once acquired, the stool sample is processed by removing particulate matter and placing it in a bacteriostatic liquid. It is then administered through either oral means (either a capsule or through artificial enteral nutrition tubes), colonoscopy, or an enema [166]. Due to the success of this practice when treating rCDI in the very first trials, FMT has been readily approved as treatment for this condition in gastroenterology guidelines [169]. This success has sparked interest in the potential of the practice to treat other conditions, including, among others, obesity and metabolic syndrome.

In their 2020 meta-analysis on the impact of FMT on metabolic syndrome parameters, Proença et al. included six RCTs [170] for a total of 154 patients with ages ranging from 18 to 69. The only significant difference between the placebo and the FMT group was HbA1c at 6 weeks, which was lower (MD = −1.69 mmol) in the FMT group. HDL levels were slightly higher in the intervention group (MD = 0.09 mmol/L), while LDL levels were lower in the placebo group. This difference was not present at 16 weeks. Fasting glucose, triglycerides, total cholesterol, BMI, and weight did not differ between the two groups. At 12 weeks, the difference in HbA1c was no longer present.

Similarly, the results of a 2024 scoping review by Horvath et al. [36] show that the procedure has yet to prove itself useful from a clinical perspective. Moreover, the authors point out that FMT is unlikely to ever be successful as a treatment for the general population suffering from various metabolic diseases, even if it does show more promising results in the future, because of how resource- and personnel-intensive it is. In the study in question, the researchers have also analyzed the efficacy of both prebiotics and probiotics on metabolic syndrome. When it comes to prebiotics, such as fructo-oligosaccharides, inulin, and galacto-oligosaccharides, they seem capable of modifying the composition of the microbiome, but they rarely seem to make a significant impact on metabolic syndrome parameters. As for probiotics, while some studies show promising results when it comes to several metabolic syndrome parameters such as body weight and fasting plasma glucose, the available data are heterogeneous when it comes to both study designs and outcomes, which makes it difficult to draw solid conclusions from.

The latest meta-analysis available by Pakhmer et al. [52] confirms the trend in the literature by analyzing 11 different studies for a total of 864 patients with ages ranging from 18 to 70 years old: insulin showed a significant decrease by 24.7 pmol/L (weighted mean difference [WMD], –24.77; 95% CI, –48.704 to –0.848) in short-term follow-up, and HDL increased by 0.1 mmol/L (WMD, 0.106; 95% CI, 0.027 to 0.184), also in short-term follow-up; both those effects tended to become irrelevant in the long term. No significant changes were seen in other metabolic parameters (lipid profile, blood glucose, insulin resistance) and in anthropometric indices (weight, BMI, circumferences). In addition, multiple studies reported gut microbiota alterations after the intervention, including an increase in butyrate-producing species.

In conclusion, fecal microbiota transplantation (FMT) has garnered interest as a potential therapeutic approach for metabolic syndrome. However, current evidence indicates that the effects of FMT on metabolic parameters remain inconclusive and somewhat limited. The meta-analysis by Proença et al. demonstrated that while FMT can temporarily improve certain parameters, such as HbA1c and lipid profiles, these effects tend to diminish over time. Similarly, the results from the 2024 scoping review by Horvath et al. suggest that FMT is not yet proven to be clinically beneficial for MetS and is unlikely to become a widely applicable treatment due to its resource-intensive nature.

While some studies show promising results, particularly in short-term improvements in specific metabolic markers, the overall evidence does not strongly support FMT as a reliable treatment for MetS. More research with larger sample sizes and longer follow-up periods is needed to determine the true efficacy and potential of FMT in managing metabolic syndrome, especially in patients confirmed to have significant gut dysbiosis.

#### Probiotics

In an animal model study, Wang et al. [171] investigated the effects of the supplementation of three different probiotic strains for 12 weeks (*Lactobacillus paracasei* CNCM I-4270, *L. rhamnosus* I-3690, and *Bifidobacterium animalis* subsp. *lactis* I-2494) in a high-fat diet (HFD): forty mice were divided into five groups each of eight mice; one group was administered with a standard diet (control) and placebo, one group with HFD and placebo, and the other three groups with HFD and a different probiotic strain each. Each group supplemented with probiotics in HFD showed reduced weight gain, less macrophage infiltration of adipose tissue, and improved glucose–insulin homeostasis in comparison to the HFD group without supplementation. Weighted analysis based of fecal bacterial genes showed that the supplementation of each of the three probiotic strains tended to shift the composition of the HFD-disrupted gut microbiota toward the microbiota of a control mouse (fed with a standard diet and no probiotic). *L. paracasei* and *L. rhamnosus* supplementation was associated with increased cecal acetate, but no significant effect was found on circulating lipopolysaccharide-binding protein; in contrast, *Bifidobacterium* supplementation did not increase acetate but significantly decreased adipose and hepatic TNF-α gene expression. This animal model study suggests that *Lactobacillus* and *Bifidobacterium* supplementation differentially attenuate obesity comorbidities by reprogramming the gut microbiota composition.

A recent systematic review and meta-analysis by Hadi A. et al. [37] investigated the correlation between probiotic supplementation and anthropometric and metabolic parameters in metabolic syndrome. A total of nine RCTS, with a total of 344 subjects, were selected for the final analysis. The total daily dose of probiotic consumption was variable between 10^6^ and 10^11^ colony-forming units (CFU) in the different studies; five studies were about a single strain of probiotics (two studies with *Lactobacillus Plantarum*, two with *Lactobacillus casei* Shirota, and one with *Lactobacillus acidophilus*), and four were characterized by a supplementation of multiple strains of probiotics (mainly *Lactobacilaceaei*, *bifidobacteriaceae*, and *Streptococcus thermophilus*); adults with MetS who received the supplementation with pro- and synbiotics had lower total cholesterol (TC) compared to placebo (MD: −6.66 mg/dL, 95% CI: −13.25 to −0.07, *p* = 0.04, I^2^ = 28.8%, n = 7), but no effect was observed on the majority of the anthropometric and metabolic outcomes considered (weight, waist circumference, body mass index, fasting blood sugar, insulin resistance, insulin, triglyceride, low-density lipoprotein cholesterol, or high-density lipoprotein cholesterol; *p* > 0.05). The authors suggested that larger sample sizes may be useful to confirm or identify the utility of probiotics in MetS.

*Lactobacillus casei Shirota* was investigated as a beneficial probiotic in metabolic syndrome by three studies by Leber et al. [38], Tripolt et al. [39]., and Stadlbauer et al. [40], but no significant evidence was found on the impact of supplementation of three servings (65 mL each) of probiotic-enriched milk with 10^8^ cells/mL on metabolic and anthropometric parameters.

Barreto FM et al. [41] investigated the impact of fermented milk with *L. plantarum* on metabolic parameters in postmenopausal women with metabolic syndrome: 24 women were recruited and paired by age, ethnicity, and body mass index in two groups; one group of 12 received 80 mL/day of fermented milk, with 1.25 × 10^7^ UFC/g of *Lactobacillus plantarum,* and the other a placebo made of 80 mL of non-fermented milk, both for 90 days (3 months). A significant reduction in glucose and homocysteine levels was observed in the treatment group compared with the placebo group (*p* = 0.037 and *p* = 0.019, respectively), but no significant difference was found for total cholesterol, blood lipids, or inflammatory biomarkers.

Another study by Benini et al. [42] investigated the potential role of probiotic supplementation in metabolic syndrome: 51 patients with Mets were divided into two groups, one receiving placebo (n = 25) and the other (n = 26) receiving a daily serving of fermented milk with *Bifidobacterium Lactis HN019* (80 mL per dose with 2.72 × 10^10^ colony-forming units); after the 45-day trial, subjects in the treatment group showed a significant decrement in body mass index (*p* = 0.017), total cholesterol (*p* = 0.009), and low-density lipoprotein (*p* = 0.008) compared with the baseline and control group, as well as a significant reduction in pro-inflammatory cytokines (tumor necrosis factor-α (*p* = 0.033) and interleukin-6 (*p* = 0.044)).

Guevara-Cruz et al.’s [172] study included three stages: first, a cross-sectional study that determined the prevalence of metabolic syndrome in a sample of 1065 individuals, then a pragmatic study that investigated the effects of lifestyle interventions in a sample of 146 subjects with metabolic syndrome, and finally a randomized, double-blind, placebo-controlled study on the effects of lifestyle interventions in a sample of healthy subjects, subjects with metabolic syndrome, and subjects with metabolic syndrome and class III obesity, totaling 171 individuals. The cross-sectional study found a prevalence of 53% for metabolic syndrome and a direct correlation between BMI and gut dysbiosis. The pragmatic study found that after a hypocaloric, low-saturated-fat diet, plus the consumption of functional foods (that included inulin, nopal, chia seeds, and oats), the prevalence of metabolic syndrome was reduced by 44.8%, while the randomized trial analyzed the gut microbiota of the subjects and discovered that, after the lifestyle intervention, there was a decrease in gut dysbiosis caused by a lower ratio of Prevotella to Bacteroides and an increase in the number of *Faecalibacterium prausnitzii* and *Akkermansia muciniphila*.

In a systematic review by Cheng et al. [33], the authors analyzed eleven RCTs for a total of 608 patients in order to determine the effects of probiotics and synbiotics on MetS. Supplementation with probiotics was determined to have a statistically significant effect on lowering BMI, LDL-c levels, and fasting blood glucose but did not achieve better results than placebo when it came to lowering blood pressure, although the authors attributed the result to a significantly high level of heterogeneity among the studies that were selected. While this systematic review both had a bigger sample size than most others on the same subject and found lower heterogeneity for all the parameters successfully lowered, one of the limitations it had was that the time frame of the studies analyzed did not extend more than three months, which is probably not enough to see a significant reversal in the parameters of metabolic syndrome, nor, on the other hand, is it enough to determine whether the effects regress to the mean after more time has passed.

Wastyk HC et al. [43] ran an RCT for eighteen weeks on 39 adults with elevated parameters for metabolic syndrome. Sixteen adults were randomly assigned to the placebo arm, while twenty-three individuals received the probiotic selected for study, which was a mixture of three different strains (*Limosilactobacillus reuteri* NCIMB 30242, *Lactiplantibacillus plantarum* UALp-05™, and *Bifidobacterium animalis* subsp. *lactis* B420™) that were already reported to have significant positive effects on metabolic syndrome parameters. Patients in both arms of the study were instructed to not undergo behavioral changes pertaining to food intake or physical exercise. The study found no statistically significant effect for blood pressure, fasting blood glucose, triglycerides, HDL-c, or waist circumference. The authors then performed hierarchical clustering of the patients in terms of response and found that the intervention group could be split between responders who saw within-patient differences in diastolic blood pressure and triglycerides and non-responders. However, the *p*-values of this analysis are close to non-significance, and splitting the group between these two categories revealed that non-responders tended to become worse as time went on during the intervention. In conclusion, while the data are compatible with there being individuals who are responders and some who are not, the low level of statistical significance, paired with there being effects only on two parameters (one of which is only one kind of blood pressure), the data from this study suggest that the blend of probiotics in question does not have a significant effect on MetS parameters.

As far as probiotics are concerned, in conclusion, research has identified several specific probiotic strains that may be particularly effective in managing metabolic syndrome (MetS). Here are some of the notable strains and their potential benefits: *Lactobacillus acidophilus*: This strain has been shown to improve glycemic control and reduce body fat in various studies [34]. It may help in managing insulin sensitivity and lowering cholesterol levels, making it beneficial for individuals with MetS [33]. *Lactobacillus casei*: Like *L. acidophilus*, *L. casei* has demonstrated antidiabetic and anti-inflammatory effects in animal models [34]. It has been associated with improvements in glucose metabolism and lipid profiles [33]. *Bifidobacterium animalis* subsp. *lactis* (B420): This specific strain has been noted for its ability to counteract the negative effects of high-fat diets, thereby improving metabolic parameters associated with MetS [35]. *Akkermansia muciniphila*: Emerging research highlights this strain’s potential in enhancing glucose metabolism and reducing insulin resistance. It has shown promise in animal studies for its ability to ameliorate type 2 diabetes symptoms [34]. *Bifidobacterium bifidum*: This strain is often included in probiotic formulations aimed at improving gut health and may also contribute positively to metabolic health, although specific studies are still needed to confirm its efficacy in MetS [33].

Probiotics exert their beneficial effects through several mechanisms: modulation of gut microbiota (probiotics help restore a healthy balance of gut bacteria, which is often disrupted in individuals with MetS); reduction in inflammation (many probiotics have anti-inflammatory properties, which can help mitigate the chronic inflammation associated with MetS); and improvement of metabolic parameters (probiotics can influence lipid metabolism, glucose homeostasis, and body weight regulation, contributing to better overall metabolic health).

### 4.2. Diabetes

More than 400 million people worldwide (about 9% of adults aged 20–79) suffer from diabetes (425 million according to 2017 data published by Forouhi et al. in 2019; 463 according to Saeedi et al., 2019), and the number will rise to about 700 million by 2045 [173,174].

Regarding the relationship between type 1 diabetes mellitus and alterations in microbiota, the review by Han et al. reports on the various studies evaluating the relationship between the gut microbiota and type 1 diabetes mellitus using genome sequencing methods such as metagenomics and 16S rRNA sequencing, from the perspective of stability, connectivity, abundance, and composition of the gut microbiota. In this review, the group highlights how dysbiosis (understood as an alteration in the composition of the gut microbiota) is associated with the pathogenesis of type 1 DM with regard to insulin dysfunction, so it is hypothesized that the microbiota may be a therapeutic target [175].

Furthermore, the scientific literature reports a change in composition, with a reduction in the phylum of Firmicutes and an increase in that of Bacteroidetes, as demonstrated in a study by Pellegrini et al. in 2017, in which they assessed the microbiota composition and inflammatory status of 19 patients with DM1, 19 with celiac disease, and 16 healthy controls. The authors analyzed duodenal mucosa biopsies by cytokine analysis (with increases in patients with DM1 in CCL13, CCL19, CCL22, CCR2, COX2, IL4R, CD68, PTX3, TNFα and VEGFA), performed an evaluation by immunohistochemical analysis of the inflammatory cells (with increased infiltration of the monocyte/macrophage lineage in DM1 patients), and performed an evaluation of microbiota (with increased Firmicutes and consequent increase in the Firmicutes/Bacteroidetes ratio and reduction in Proteobacteria and Bacteroidetes); these features delineate a specific inflammation picture of DM1 patients compared to controls (i.e., celiac disease patients and healthy controls) [54].

In a 2015 study, Alkanani et al. analyzed patients with DM1, evaluating their microbiota by high-throughput sequencing of bacteria 16S rRNA genes. The study correlated the microbiota of newly diagnosed (<6 months) DM1 patients with healthy controls, relatives of diabetic individuals with the HLA3/4 serotype, and relatives with negative HLA3/4 serotype, with the following results: subjects with HLA3/4+ had increased Bacteroidetes, RC9 family, *Prevotellaceae*, and *Catenibacterium* family, while HLA3/4− subjects had decreased *Prevotellaceae*. It was therefore hypothesized that the altered microbiota may correlate with a different susceptibility to the onset of DM1 and its progression [56].

Furthermore, there are studies that have shown an increase in the F/B ratio in children who were later diagnosed with DM1: in a 2018 study by Leiva-Gea, for example, comparing, via RNA sequencing (16S rRNA pyrosequencing), the microbiota of subjects with DM1 (15 subjects) and MODY2 (maturity-onset diabetes of the young, a type of monogenic diabetes; 15 subjects) and healthy controls (13 subjects), a different composition was seen in DM1 subjects as follows: a reduction in microbiota diversity, an increase in *Bacteroides*, *Ruminococcus*, *Veillonella*, *Blautia,* and *Streptococcus*, a reduction in *Bifidobacterium*, *Roseburia*, *Faecalibacterium,* and *Lachnospira*, and an increase in pro-inflammatory cytokines and lipopolysaccharides. In MODY2 subjects, on the other hand, there was an increase in Prevotella and a reduction in *Ruminococcus* and *Bacteroides*. In both DM1 and MODY2, an increase in intestinal permeability was seen [176].

Despite these results, there is no unanimity regarding the correlation: some studies report a positive association between DM1 and Proteobacteria abundance (e.g., the study by Brown et al. of 2011, which studied the microbiota of DM1 patients and healthy controls using metagenomics and 16S rRNA sequencing, showing that DM1 patients had an increase in Actinobacteria, Bacteroidetes and Proteobacteria, while controls had an abundance of *Firmicutes*, *Fusobacteria*, *Tenericutes*, and *Verrucomicrobia*), others a negative association (such as Leiva-Gea et al. of 2018, see above), and others no relation (such as Murri et al. of 2013, in which they again compared by 16S rRNA sequencing the microbiota of 16 children with DM1 and 16 healthy children, and found differences in microbiota with regard to other bacterial strains: in DM1, there was an increase in Actinobacteria and Firmicutes and thus an increase in the Firmicutes/Bacteroidetes ratio, and a reduction in Bacteroidetes, while the amount of Proteobacteria was similar to that of the control group) [58,177].

In the 2021 review by Mokhtari et al., it was shown that DM1 patients have greater abundance of twelve genera, namely *Bifidobacterium*, *Bacteroides*, *Escherichia*, *Veillonella*, *Clostridium*, *Enterobacter*, *Lactobacillus*, *Ruminococcus*, *Streptococcus*, *Sutterella*, *Lactococcus*, and *Blautia*, in addition to Bacteroides, which is always the most represented genus [62].

According to a 2021 review, there is a relationship between the relative abundance of *Bifidobacterium* and elevated risk of DM1, although the causal relationship between the presence of a bacterium in the microbiota and phenotypic exposure is still unclear, so further studies will be needed [178].

Furthermore, there are various factors that can influence the composition of microbiota and the onset of diabetes; for instance, in the 2016 Livanos et al. animal model study, they showed that the incidence of DM1 increased in mice that received antibiotics. Livanos’ group used various methods, such as flow cytometry (immune phenotyping by flow cytometry) and analysis via microarray and qPCR for gene expression at the level of the ileum, surface stains for the expression of CD3 and CD4 on T-helper cells, and staining for the expression of FOXP3 and RORγT to identify T-reg and Th17 cells, respectively. The objective was to evaluate the effects of antibiotics in sub-therapeutic continuous (STAT) or therapeutic pulsed (PAT) doses on NOD (non-obese diabetic) susceptible DM1 mice that are genetically susceptible to T1D, with the following results: treatment with antibiotics early in life effectively altered the composition of the gut microbiota and its metabolic capacity, the intestinal expression of several genes, and the T-cell populations present, accelerating the onset of DM1 in mice [179].

With regard to the relationship between type 2 diabetes mellitus and alterations in microbiota, as in DM1, there is also a difference in DM2 with the microbiota of healthy individuals, as demonstrated in the Integrative Human Microbiome Project (IR), in which the researchers found differences in molecular and microbiota patterns between the two groups of individuals with regard to beta-diversity (while alpha-diversity seemed unchanged). In particular, Que et al. in 2021 carried out a study by meta-analysis of seven different studies (a total of 600 DM2 cases, 543 healthy controls, i.e., 1143 samples in total) to assess the composition of microbiota in DM2 by means of NGS for the 16s rRNA gene, with the following results: relative increase in Firmicutes (class *Negativicutes*, order *Selenomonadales*, family *Veillonellaceae*) and Actinobacteria and relative decrease in Bacteroidetes (class *Bacteroidia*, order *Bacteroidales*) in DM2 patients [53,180].

From the point of view of genome analysis, there are conditioned pathogens (i.e., organisms that only cause disease in the presence of specific predisposing factors, such as *Escherichia coli*, *Bacteroides caccae*, *Clostridium*, *Eggerthella*, and *Proteobacteria*) and other bacteria such as *Lactobacillus gasseri*, *Streptococcus mutans*, some *Clostridial* species, and *Lactobacillus*, as shown in a 2022 review that considers various studies comparing the microbiota of DM2 patients with that of healthy controls [61].

Furthermore, a 2023 review by Crudele et al. describes how the presence of bacteria such as Bacteroidetes influences microbiota, being Gram-negative bacteria whose presence correlates with increased LPS; there is consequently inflammation precisely in relation to LPS, with production of pro-inflammatory cytokines such as IL-1, IL-6, and TNF-α (tumor necrosis factor-α), which appear to play a role in the pathogenesis of insulin resistance [60].

On the contrary, the presence of butyrate-producing bacteria such as *Roseburia intestinalis*, *Roseburia inulinivorans*, *Eubacterium rectale*, *Faecalibacterium prausnitzii*, *Bacteroides* and *Clostridiales* sp. SS3/4 and other bacteria such as *Akkermansia mucinphila* (which stimulates mucus production by the intestinal mucosa through degeneration of intestinal mucin) was reduced, as shown in a 2012 study by Qin et al., in which they studied the microbiota of DM2 patients (specifically, 345 individuals) using an MGWAS (metagenome-wide association study) [181].

In 2018, Allin et al. studied the microbiota of subjects with prediabetes and healthy controls (134 vs. 134, respectively) by 16S rRNA gene sequencing, showing that, in subjects with prediabetes, there was a reduction in Clostridium and *A. muciniphila* [75].

This imbalance of the intestinal microbiota is due to various factors such as drugs (including the excessive use of antibiotics), the type of birth (cesarean section vs. eutocic birth), nutrition of the newborn, diet in adults, stress and anxiety, genetic factors and any associated pathologies, lifestyle, and geographical origin, as described by Bajinka et al. in a 2023 review [59].

The relationship between microbiota and DM2 is confirmed in several aspects by many studies, e.g., the reviews by Bajinka et al. in 2023 and Ye et al. in 2022, with regard to, e.g., insulin resistance and inflammation, changes in metabolism, modulation of glucose metabolism, and changes in microbiota during the development of DM, although the mechanisms in the pathogenesis of diabetes itself have not yet been fully clarified. Furthermore, the correlation between the gut microbiota and DM complications such as diabetic retinopathy, diabetes-induced cognitive impairment, diabetic peripheral neuropathy, and diabetic nephropathy has also been demonstrated [59,61].

There is a great deal of evidence linking the development of DM to intestinal dysbiosis, linked to the entry of antigens into the circulatory system through the intestinal mucosa, which then causes an immune response trigger that damages the beta cells of the pancreatic islets. These antigens, in addition to direct damage to the beta cells of the pancreas, can also trigger autoimmunity in the pancreatic islets themselves, and metabolites from the gut microbiota can have hormonal effects that also cause other metabolic disorders [63,182].

Diet and gut microbiota, when altered, cause low-grade systemic inflammation that can lead to insulin resistance through increased levels of pro-inflammatory cytokines in the circulatory system, such as TNF-α, IL-6, β kinase inhibitor (IKKβ), and c-Jun N-terminal kinase (JNK). All these cytokines can activate the insulin receptor substrate (IRS), exerting a negative effect on insulin signaling, which can cause insulin resistance [59].

The gut microbiota produces active metabolites, including short-chain fatty acids (SCFAs), ammonium, phenols, endotoxins, etc., via macronutrients from the diet, and are preventive against the development of DM, while other substances such as branched-chain amino acids (BCAAs), phenols, p-cresols, methane, amines, and ammonium promote the progression of DM [183,184].

Two receptors for SCFAs, GPR41, and GPR43 (free fatty acid receptor 3 and 2, respectively), expressed in enteroendocrine, intestinal epithelial, and pancreatic islet tissues, have been identified as being directly implicated in the development of DM2. Activation of GPR41 stimulates the secretion of leptin (which regulates energy expenditure and long-term food intake) and peptide YY (which increases satiety), in addition to activation of the orthosympathetic system, which stimulates energy expenditure and reduces the risk of DM2. Activation of GPR43 increases insulin secretion, reduces glucagon secretion and increases satiety. There are studies in animal models such as that of Bjursell et al. in 2011, in which they showed that in mice transgenic for GPR43, there is an improvement in metabolic parameters, such as reduced obesity, increased homeostasis, increased quality of lean mass, and increased secretion of GLP-1 [177,185,186].

There have been several studies correlating the effect of dietary fiber with a positive effect on metabolism, reducing insulin resistance in DM2 patients, as demonstrated in a 2020 review by Silva’s group. Furthermore, there are correlations with the biodiversity of microbiota and the production of favorable metabolites, such as butyrate [187].

In particular, microbiota-accessible carbohydrates (MACs) in dietary fiber are the compounds that most influence and determine the composition of the gut microbiota, expanding the variety of bacterial species.

In a 2014 controlled clinical trial, David et al. examined a group of healthy volunteers (six males and four females, aged 21 to 33 years, BMI between 19 and 32 kg/m^2^), who followed two diets—one ‘plant-based’, rich in cereals, legumes, fruit, and vegetables, and the other ‘animal-based’, rich instead in meat, eggs and cheese—demonstrating by means of microbiota analysis (by means of 16S rRNA sequencing, the specific software ‘Insights Into Microbial Ecology (QIIME)’, some specific Python scripts, the analysis of SCFAs by gas chromatography, and the analysis of bile acids by enzymatic analysis and mass spectrometry) the rapid change in microbiota composition with the change from one diet to another. In particular, Prevotella abundance correlates with a higher dietary fiber intake, while the amount is lower in the animal-based diet than in the vegetarian diet [57].

Many antidiabetic drugs also have action on the gut microbiota, such as metformin, liraglutide, acarbose, and thiazolidinedione, as reported in the 2020 review by Gurung et al. [78,188].

The study by Smits et al. in 2021 enrolled 51 DM patients treated with GLP1 agonists (glucagon-like peptide-1 receptor agonists, such as Liraglutide) or DPP-4 inhibitors (dipeptidyl peptidase-4 inhibitors, such as Sitagliptin) to analyze any changes in microbiota and found that there were no statistically significant changes [189].

Other drugs, such as acarbose, have instead demonstrated an effect on microbiota: in the 2021 study by Takewaki et al. on 18 patients with DM2, it was shown that there was an increase mainly in Actinobacteria and Bacteroidetes after 4 weeks of treatment with acarbose [190].

A 2023 review by Crudele et al. explained how metformin can influence microbiota; e.g., this drug is associated with the strengthening of tight junctions, thus counteracting one of the mechanisms related to intestinal dysbiosis. In addition, there is also a change in the composition of the microbiota itself, with a tendency to more closely resemble that of a healthy individual: in fact, it reduces the levels of *Clostridium bartlettii* (whose presence correlates with insulin resistance), thus increasing insulin sensitivity; it increases the presence of Enterobacteriales, *Akkermansia muciniphila*, Escherichia coli, and Ruminococcus, while it reduces *Intestinibacter bartlettii* and *Roseburia intestinalis*, thus having a protective effect towards intestinal eubiosis with regard to the relative abundance of 80 species of bacteria [60].

In the 2018 study by Bauer et al. in animal models, the group investigated the effects of metformin on the gut microbiota and glucose metabolism: germ-free mice received a fecal transplant from metformin-treated donors, and subsequently the hosts showed increased glucose tolerance. In addition, *Lactobacillus* levels in the first tract of the small intestine were increased, and there was a restoration of sodium–glucose cotransporter-1 (SGLT1) expression, thus increasing glucose sensitivity [191].

In the study by Wu et al. of 2017, they studied the effects of metformin treatment on the gut microbiota, analyzing it by means of 16S rRNA sequencing, metagenomics, hematochemical examinations, and glucose tolerance tests in a double-blind study (22 controls given placebo and 18 given metformin), with the following results: an increase in beneficial bacteria such as Akkermansia and a reduction in pathogenic bacteria, as well as effects on glucose metabolism such as increased glucose metabolism and glucose sensitivity [55].

Concerning fecal transplantation in diabetes, as described in Ye et al.’s review of 2022, through studies in animal models, it has been shown that FMT early in life can significantly delay the development of DM1 in both MyD88-deficient mice and NOD mice receiving FMT with non-selective human microbiota. In contrast, the incidence of DM1 is increased when mice receive antibiotics. [61] In the same review, they also report on a possible use of auto-FMT in humans with DM1: in fact, it was shown how, in patients newly diagnosed with DM1 (<12 months after onset), a fecal self-transplant can prevent the reduction in endogenous insulin secretion, thus suggesting how this can prevent β-cell damage [61].

Recently, several studies have also been conducted on the link between FMT and DM2: in Yej’s review of 2022, it is reported that fecal transplantation can reduce hyperglycemia, improve insulin resistance and insulin sensitivity, inhibit levels of chronic pancreatic inflammation, reduce β-cell apoptosis, increase beneficial bacteria such as *Bifidobacterium*, and reduce sulfate-reducing bacteria (Desulfovibrio and Bilophila) [61].

FMT can have problems with gut colonization, but repeated FMT transplants can significantly increase microbiota engraftment, and the combination of transplantation with lifestyle and diet intervention has shown better results [61].

#### Probiotics

There have been several studies on animal models showing that oral administration of probiotics increases anti-inflammatory cytokines such as TGF-β and IL-10, reduces pro-inflammatory cytokines such as TNF-α, IL-6, and IL-1β, and regulates the balance of Th1/Th2/Th17/Treg cells [192].

In a 2011 study by Lau et al. in animal models (specifically, mice referred to as BBDP, or Bio-Breeding diabetes-prone rats), they investigated the effects of supplementation with *Lactobacillus johnsonii* strain N6.2 (LjN6. 2, with doses of 1 × 3 10^8^ CFU) in the modulation of the immune response in DM1 by means of lymphocyte profiling, cytokine levels, analysis of lymph nodes and spleen, analysis of cytokines by ELISA, stu4dy of dendritic cells and T cells, and in vitro and in vivo immuno response assays. Lau’s group demonstrated that LjN6.2 modulated an immune response that conferred resistance to DM1 by influencing the differentiation of Th17 cells, with high levels of 17, IL-23R, IL-6, and IL-23 [192].

In a 2020 meta-analysis by the Kocsis group, 32 clinical trials (RCTs) evaluating the effect of probiotics as supplementation in individuals with DM2 were analyzed, with a total of 1676 patients considered. The probiotics considered in the various studies were mainly *Lactobacillus, Bifidobacterium, Saccharomyces, Enterococcus, E. coli, and Streptococcus*, administered tendentially in cocktails containing 2–17 species. The meta-analysis assessed BMI, total cholesterol, LDL, triglycerides, HDL, PRC, HbA1c, fasting blood glucose, fasting insulinemia, and systolic and diastolic blood pressure, indeed demonstrating a statistically significant positive effect on all parameters except BMI and LDL [70].

In a 2022 meta-analysis, the group of Zhang et al. focused on the dosage at which probiotics are effective in DM2 patients, evaluating 33 trials with a total of 1927 patients. The probiotics consisted of *Bifidobacterium, Lactobacillus*, and yeast, with an average dose of 10^10^ CFU/day and an average treatment duration of 8 weeks; the significant results were the reduction in glycated hemoglobin levels by 0.19%, fasting blood glucose by 1.00 mmol/L, fasting insulinemia by 5.73 pmol/L, and HOMA-IR by 1.00. Furthermore, the type of bacteria also determines the efficacy of probiotic therapy: Brewer’s yeast reduces fasting glycaemia, whereas *Lactobacillus casei* and *Lactobacillus sporogenes* have no statistically significant effects when administered as monotherapy (single strain formulas) [68].

In a 2022 meta-analysis by Naseri et al., 46 papers (sample size of 3067 patients) on probiotics and synbiotics were studied, concluding that these significantly reduced glycated hemoglobin HbA1c and the HOMA-IR index, while there was no effect on the glycemic curve after OGTT glucose loading. Both probiotic cocktails and single-strain synbiotics (all types of supplementation: tablets, capsules, powders, bread, milk, yogurt and honey) were included in the study [69].

In the meta-analysis by Ayesha et al. of 2023, 22 RCTs investigating the administration of probiotics (mainly various strains of *Lactobacillus* and *Bifidobacterium*) in a total of 2218 DM2 patients, both short- (8 weeks maximum) and long-term (at least 12 weeks), were analyzed, which demonstrated a significant improvement in HOMA-IR, HbA1c, and fasting blood glucose. The studies compared different therapies in terms of duration and the strains of probiotics used in relation to the effects on the parameters considered but did not clearly refer to greater efficacy on the basis of short vs. long duration or the strains examined. The strains considered are as follows:

*Bifidobacterium: B. animalis* subsp. *lactis* (BB-12, M8, W52), *B. animalis* (dn-173 010, V9, subsp. *lactis*), *B. longum*, *B. breve*, *B. breve* ceppo Yakult, *B. bifidum* (W23), and *B. infantis*.

*Lactobacillus*: *L. paracasei* (strain Shirota, HII01), *L. rhamnosus* (GG, Probio-M9), *L. casei* (UBLC42, Zhang, W56), *L. plantarum* (UBLP40, P-8, A7), *L. bulgaricus*, *L. acidophilus* (UBLA34, W37, La-5), *L. salivarius* (UBLS22, W24), *L. brevis* W63, *L. reuteri* DSM 17938, and *L. lactis* (W58, W19).

*Streptococcus thermophilus*, *Bacillus coagulans*, *Clostridium butyricum*, *C. beijerinckii*, *Akkermansia muciniphila*, and *Anaerobutyricum hallii*.

Other compounds: fructo-oligosaccharide, galacto-oligosaccharide, and berberine [67].

In a 2023 meta-analysis by Li et al., they evaluated 30 RCTs studying the efficacy of probiotics in 1827 DM2 patients, with an improvement in glycemic control (reduction in fasting blood glucose, insulinemia, HbA1c, HOMA-IR). They divided the RCTs considered according to various parameters: ethnicity (Caucasian vs. Asian), dose of probiotic (> or <10^10^ CFU/day), duration of intervention (> or < of 8 weeks), type of probiotic (*Lactobacillus* vs. *Lactobacillus + Bifidobacterium*), type of vehicle used for administration (powder/tablet/capsule vs. food), and BMI at baseline (> or <30 kg/m^2^). The best results were seen in subgroups of patients with Caucasian ethnicity, higher BMI at baseline (BMI ≥ 30.0 kg/m^2^), and use of probiotics with *Bifidobacterium*. On the other hand, there were no differences between high and low doses of probiotics or between short and longer duration of the intervention [65].

In a 2023 meta-analysis by Moravejolahkami et al., the group studied the effect of prebiotics, probiotics, and synbiotics on the microbiota of DM1 patients regarding parameters such as fasting blood glucose (FBG), HbA1c, C-peptide, and the need for insulin therapy by means of a meta-analysis of five RCTs (total subjects studied: 356). The probiotic strains examined were the following: *L. sporogenesis* GBI-30, *L. paracasei* DSM 24733, *L. plantarum* DSM 24730, *L. acidophilus* DSM 24735, *L. delbrueckii* subsp. *bulgaricus* DSM 24734, *B. longum* DSM 24736, *B. infantis* DSM 24737, *B. breve* DSM 24732, *Streptococcus thermophilus* DSM 24731, *L. salivarius* subsp. *salicinius* AP-32, *L. johnsonii* MH-68, and *B. animalis* subsp. *lactis* CP-9. The only statistically significant results concerned fasting blood glucose, whereas there were no significant results concerning glycated hemoglobin, C-peptide, and insulin dose [66].

In a 2024 meta-analysis by Wang et al., the effects of different probiotics on individuals with DM2 (following PRISMA guidelines, using RevMan 5.4 software, and assessing the risk of bias using the Cochrane Handbook for Systematic Reviews 5.1.0) were evaluated, including eight RCT studies with a total of 507 patients, demonstrating a positive effect on glycated hemoglobin HbA1c, insulinemia, and homeostatic model assessment for insulin resistance (HOMA-IR) levels, while there was no statistical significance for fasting blood glucose and BMI. Below are the studies evaluated in the meta-analysis with the probiotics examined: Asemi et al., 2013 (*L. acidophilus* 2 × 10^9^ CFU, *L. casei* 7 × 10^9^ CFU, *L. rhamnosus* 1.5 × 10^9^ CFU, *L. bulgaricus* 2 × 10^8^ CFU, *Bifidobacterium breve* 2 × 10^10^ CFU, *B. longum* 7 × 10^9^ CFU, and *Streptococcus thermophilus* 1.5 × 10^9^ CFU); Mazloom et al., 2013 (*L. acidophilus*, *L. bulgaricus*, *L. bifidum*, and *L. casei*); Tonucci et al., 2017 (*Lactobacillus acidophilus* La-5 10^9^ CFUs, 83 *Bifidobacterium* animal subsp 10^9^ CFUs, and Lactis BB-12); Firouzi et al., 2017 (*Lactobacillus*, *Firmicutes phyla*, *Bifidobacterium*, and *Actinobacteria phyla*); Razmpoosh et al., 2019 (*Lactobacillus acidophilus* 2 × 10^9^ CFU, *L. casei* 7 × 10^9^ CFU, *L. rhamnosus* 1.5 × 10^9^ CFU, *L. bulgaricus* 2 × 10^8^ CFU, *Bifidobacterium breve* 3 × 10^10^ CFU, *B. longum* 7 × 10^9^ CFU, and *Streptococcus thermophilus* 1.5 × 10^9^ CFU); Kobyliak et al., 2020 (*Lactobacillus* 1.0 × 10^9^ CFU/g, *Bifidobacterium* 1.0 × 10^9^ CFU/g, *Lactococcus* 1.0 × 10^8^ CFU/g, *Propionibacterium* 1.0 × 10^8^ CFU/g, and *Acetobacter* 1.0 × 10^5^ CFU/g); Savytska et al., 2023 (*Lactobacillus Lactococcus* 6 × 10^10^ CFU/g, *Bifidobacterium* 1 × 10^10^/g, *Propionibacterium* 3 × 10^10^/g, and Acetobacter 1 × 10^6^/g); and Zikou et al., 2023 (*Lactobacillus acidophilus* 1.75 × 10^9^ CFU, *L. plantarum* (0.5 × 10^9^ CFU, *Bifidobacterium lactis* 1.75 × 10^9^ CFU, and *Saccharomyces boulardii* 1.5 × 10^9^ CFU) [64].

Regarding prebiotics, it is important to highlight how these are a source of fermentation products such as SCFAs, which have a positive effect on microbiota, while PUFAs (polyunsaturated fatty acids) have positive effects in terms of anti-inflammatory, antioxidant, immune modulation, anti-carcinogenic, and anti-estrogenic effects, as described by the Bajinka group in the review published in 2023 [59].

In particular, Crudele et al., in a 2023 study, also reported some data on prebiotics: complex carbohydrates, polyunsaturated fatty acids, and polyphenols increase fecal consistency and are fermented into SCFAs (short-chain fatty acids). For example, oligofructose has shown positive effects on glucose homeostasis, inflammation, leptin sensitivity, GLP-1 production, and the integrity of the intestinal epithelial barrier. Berberine, resveratrol, alliin, capsaicin, betacyanin, and cranberry proanthocyanins have demonstrated antidiabetic effects, so they could be associated with pharmacological therapies already used in DM [60].

In summary, to date, there have been seven meta-analyses on probiotics [65,66,67,68,69,70], including a total of 93 RCTs (randomized controlled trials) and 6558 patients (see table in separate file for details about RCTs considered, strains, patients, etc.). The probiotics in the studies included a large pool of bacterial species and strains; the most frequent were *Bifidobacterium* and *Lactobacillus*, as well as other bacteria such as *Streptococcus thermophilus*, *Akkermansia muciniphila*, *Clostridium butyricum*, etc., both in single administration and in cocktails with various species. Most meta-analyses agree that there are significantly positive effects on fasting blood glucose, HbA1c, and HOMA-IR, while only a few demonstrate significant effects on the remaining parameters considered (BMI, insulinemia, total cholesterol, triglycerides, HDL, PRC, systolic and diastolic blood pressure).

In conclusion, probiotics, defined as live microorganisms that provide health benefits when consumed in adequate amounts, have gained attention for their potential role in managing type 2 diabetes mellitus (T2DM). This interest is largely due to the increasing recognition of the gut microbiota’s influence on metabolic health and the pathogenesis of diabetes.

The beneficial effects of probiotics on glycemic control may be attributed to several mechanisms, such as gut microbiota modulation, where probiotics help restore dysbiosis—an imbalance in gut microbiota—which is linked to insulin resistance and obesity (specific strains, such as *Lactobacillus rhamnosus* GG and *Bifidobacterium animalis*, have been highlighted for their potential therapeutic effects); inflammation reduction, where probiotics may exert immunomodulatory effects that reduce systemic inflammation, a known contributor to insulin resistance; and cholesterol metabolism, where some studies suggest that probiotics can lower cholesterol levels by reducing intestinal absorption and inhibiting cholesterol synthesis.

Research indicates that certain probiotic strains may be particularly beneficial for managing type 2 diabetes mellitus (T2DM). The following strains have shown promise based on clinical studies and meta-analyses:

1. *Lactobacillus acidophilus*

*Lactobacillus acidophilus* is known for its potential antidiabetic effects, particularly due to its ability to improve epithelial barrier function and reduce inflammation. These effects may contribute to more effective regulation of glucose metabolism and lipid metabolism, which are critical aspects in managing type 2 diabetes mellitus (T2DM). Several studies support this perspective: research has shown that yogurt containing *Lactobacillus acidophilus* can improve the lipid profile in patients with T2DM, suggesting the therapeutic potential of this probiotic in modulating metabolic parameters.

2. *Lactobacillus plantarum*

*Lactobacillus plantarum* plays a role in the regulation of glucose metabolism and has shown potential benefits in reducing low-grade inflammation, a factor often associated with insulin resistance. Although human trials remain limited, animal studies suggest promising outcomes, indicating that *Lactobacillus plantarum* may help mitigate hyperglycemia and improve insulin sensitivity. These findings underscore the potential of this strain in supporting metabolic health, particularly in conditions related to impaired glucose regulation.

3. *Bifidobacterium lactis*

*Bifidobacterium lactis* has been associated with improvements in key metabolic parameters, including reductions in fasting blood glucose (FBG) and glycated hemoglobin (HbA1c) levels. This strain is frequently included in probiotic formulations designed to enhance glycemic control, highlighting its potential role in supporting better management of blood sugar levels and overall metabolic health.

4. *Lactobacillus rhamnosus* GG

*Lactobacillus rhamnosus* GG has been associated with improved glycemic control and may contribute to reducing inflammation related to diabetes. Studies have highlighted this strain as a beneficial adjunct therapy for managing type 2 diabetes mellitus (T2DM), suggesting its potential role in enhancing traditional treatment approaches by supporting both metabolic and inflammatory regulation.

5. *Saccharomyces boulardii*

*Saccharomyces boulardii*, although primarily studied in animal models, shows potential in promoting metabolic health and positively influencing gut microbiota composition. Research indicates that this strain may aid in regulating glucose levels, though further human studies are necessary to confirm these effects. Its promising impact on metabolic parameters suggests it could be a valuable addition to therapeutic strategies aimed at supporting glucose regulation and overall metabolic health.

The administration of these probiotics has been linked to significant improvements in metabolic profiles, including reductions in HbA1c, FBG, and insulin resistance markers like HOMA-IR. However, the efficacy can vary based on the specific strains used, dosage, and individual patient factors. Further research is necessary to establish standardized recommendations for probiotic use in diabetes management. Incorporating these probiotics into dietary strategies may offer a supportive approach to enhancing glycemic control alongside conventional diabetes therapies.

### 4.3. Prediabetes

Evidence for the involvement of the gut microbiota in the regulation of glucose metabolism and the progression of type 2 diabetes mellitus (T2DM) is accumulating. Understanding microbial dysbiosis and the specific alterations in gut microbiota composition that occur during the early stages of glucose intolerance is of paramount importance, as recently reported in the systematic review by Letchumanan [77].

In the pathogenesis of prediabetes and diabetes, inflammatory response, nutrition, intestinal permeability, glycolipid metabolism, insulin sensitivity, and energy homeostasis play an important role, as reported by Gurung M’s review [78].

The gut microbiota influences host metabolic disorders through the modulation of metabolites, including SCFAs, the endotoxin LPS, BA, and TMAO, as well as by mediating the interaction between the gastrointestinal system and other organs [87].

An in vitro study demonstrated that LPS stimulates the immune system by binding to the Toll-like receptor (TLR), triggering immune cells to release inflammatory cytokines, which promote insulin resistance caused by an endotoxin-induced inflammatory response [68,193]. Furthermore, a study conducted on animal models demonstrated how the intestinal microbiota protects against the development of obesity, metabolic syndrome, and prediabetes with an immune-mediated mechanism, inducing specific commensal Th17 cells. A diet rich in fats and sugars has been shown to promote the metabolic syndrome by depleting those microorganisms that induce Th17; conversely, the recovery of commensal Th17 cells restores their protective effect. Indeed, it has been observed that the loss of protective Th17 cells is induced by diet, in particular by simple sugars. The elimination of sugar from diets has shown a protective role against obesity and metabolic syndrome in an animal model through a mechanism dependent on specific commensal Th17 cells. Sugar and ILC3 promote the growth of bacterial species, such as Faecalibaculum rodentium, which dysregulate Th17-inducing microbiota [194].

Another potential mechanism associated with intestinal ecosystem homeostasis is the endocannabinoid system. LPS interacts with endocannabinoid receptors (eCB1), modulating intestinal permeability and translocation of LPS, increasing circulating levels of LPS, and inducing metabolic endotoxemia [195].

Lipopolysaccharides (LPSs), components of the outer cell membrane of Gram-negative bacteria, can be found in high concentrations and be absorbed from the intestine [71].

The study of Larsen N et al. included 36 male adults, among which 18 subjects were diagnosed with type 2 diabetes, and investigated their fecal bacterial composition by real-time quantitative PCR (qPCR) and, in a subgroup of subjects (N = 20), by tag-encoded amplicon pyrosequencing of the V4 region of the 16S rRNA gene. The intestinal microbiota across the subjects with type 2 diabetes was relatively enriched with Gram-negative bacteria, belonging to the phyla Bacteroidetes and Proteobacteria. The main compounds of outer membranes in Gram-negative bacteria are lipopolysaccharides (LPSs), known as potent stimulators of inflammation, which can exhibit endotoxemia. Consequently, LPS will continue to be produced within the gut, which might trigger an inflammatory response and play a role in the development of diabetes.

In an animal model, it has been demonstrated that SCFAs, especially butyrate, promote the secretion of GLP-1, which prevents glucagon secretion, inhibits gluconeogenesis in the liver, and improves insulin sensitivity [196].

Additionally, again in an animal model, it has been demonstrated that SCFAs may prevent low-grade inflammation caused by the migration of bacteria from the gut into the mesenteric adipose tissue and blood [197]. In conclusion, these data from the animal model suggest that increasing SCFAs, especially butyrate, is important for preventing and controlling prediabetes.

In humans, it was observed that the number of bacteria responsible for the production of short-chain fatty acids (SCFAs) was lower in patients with T2D [2]. In particular, Qin J et al. developed a protocol for a metagenome-wide association study (MGWAS) and undertook a two-stage MGWAS based on deep shotgun sequencing of the gut microbial DNA from 345 Chinese individuals. They identified and validated approximately 60,000 type 2 diabetes-associated markers and established the concept of a metagenomic linkage group, enabling taxonomic species-level analyses. MGWAS analysis showed that patients with type 2 diabetes were characterized by a moderate degree of gut microbial dysbiosis, a decrease in the abundance of some universal butyrate-producing bacteria, and an increase in various opportunistic pathogens, as well as an enrichment of other microbial functions conferring sulfate reduction and oxidative stress resistance.

A review by AW reported that an altered gut microbiota can lead to decreased SCFA production and increased inflammation and can affect insulin secretion and pancreatic β-cell sensitivity, leading to insulin resistance [79].

Another mechanism related to the alteration of the microbiota in prediabetes involves intestinal bacteria that metabolize nutrients to produce trimethylamine (TMA), which is then converted into trimethylamine N-oxide (TMAO) in the liver. Animal models have shown that nutrient-derived TMAO can exacerbate impaired glucose tolerance and increase fasting insulin levels by blocking the hepatic insulin signaling pathway and causing inflammation in adipose tissue [198]. A prospective study demonstrated that higher intake of phosphatidylcholine (the precursor to the generation of TMAO) was independently associated with an increased risk of T2D [72]; the association between TMAO and T2D has not reached a consistent conclusion, but TMAO levels have been shown to be elevated in patients with T2D [73].

Roi et al. observed that plasma TMAO levels are associated with an increased prevalence of prediabetes in a nonlinear manner but are not related to insulin resistance or fasting plasma glucose [74].

As regards the composition of the microbiota in prediabetes, in a 2018 Danish case–control study it was demonstrated that individuals with this condition have an altered intestinal microbiota compared to healthy subjects, characterized by a decrease in the genus Clostridium and the mucin-degrading bacterium *Akkermansia munichipila*. These results are comparable to observations made in chronic diseases characterized by chronic low-grade inflammation [75].

This study analyzed the gut microbiota of 134 Danish adults with prediabetes, overweight, insulin resistance, dyslipidemia, and low-grade inflammation and 134 age- and sex-matched individuals with normal glucose regulation. Fecal microbiota composition was profiled by sequencing the V4 region of the 16S rRNA gene on an Illumina MiSeq instrument (llumina RTA v1.17.28; MCS v2.5, Illumina, Inc., San Diego, USA, and the NucleoSpin Soil kit sourced from Macherey-Nagel GmbH & Co. KG, Düren, Germany) with 515F and 806R primers designed for dual indexing and the V2 Illumina kit (2 × 250 bp paired-end reads).

A study published in the journal *eBioMedicine* in the same period came to different conclusions, especially with regard to the bacterium *Akkermansia munichipila*. The authors used a combination of in-depth metagenomics and metaproteomic analyses of stool samples from treatment-naïve (TN-DM2, n = 77), prediabetic (preDM, n = 80), and normo-glucose-tolerant (NGT, n = 97) individuals with type 2 diabetes to study the relative functional and compositional changes in the gut microbiota and fecal content of microbial and host proteins to elucidate possible host–microbial interactions, characterizing the different stages of the disease, from prediabetes to treatment-naïve DM2 [76].

In total, 11,980 meta-proteins and 425 human proteins were identified. The analysis allowed us to observe several distinctive features in preDM and NGT compared to TN-DM2, such as a higher abundance of *Akkermansia muciniphila* and a lower abundance of *Bacteroides* spp. The first is a well-known mucin-degrading bacterium, which in some studies has already been shown to be able to reduce some pathologies included in metabolic syndrome, both in mice and in humans. On the other hand, the relative abundance of several Firmicutes species which produce butyrate was lower in preDM and TN-DM2 than in NGT. These findings are in line with a gradual progression of the disease through prediabetes to overt DM2. Specifically, the authors found higher abundances of Enterobacteriaceae species (dominated by *E. coli*) and lower levels of host proteins that are potentially involved in Proteobacteria-specific responses in preDM, such as galectin-3 and proteins within the immunoglobulin superfamily. An increased abundance of intestinal Enterobacteriaceae has been widely reported in patients with metabolic diseases such as obesity and atherosclerotic cardiovascular disease and in patients with chronic inflammatory bowel disease. These unique traits, associated with preDM, could link potential gut microbial signals to an increase in chronic low-grade systemic inflammation.

The authors also noted that pancreatic enzyme content differed in fecal samples among the three groups of individuals studied. These results suggest that unique and distinctive nonlinear changes to the gut ecosystem may exist in individuals with preDM prior to transition to overt DM2. The authors of the study themselves emphasize that further large-scale longitudinal follow-up studies will be needed to delineate how microbial functions change from prediabetes to diabetes and to address the nature of the interactions between the gut microbiota and the host in the transient phases leading to frank DM2, also in the future therapeutic perspective.

A subsequently published systematic review (2022) summarized the existing evidence regarding the composition and diversity of the microbiota in individuals with prediabetes (preDM) and individuals newly diagnosed with T2DM (newDM) compared to individuals with normal glucose tolerance (nonDM). A total of 18 observational studies (5489 participants) that analyzed the gut microbiota of subjects with preDM and newDM were included. Low microbial diversity was found overall in both preDM and newDM compared with nonDM. Differences in gut microbiota composition between preDM and newDM and those with nonDM were inconsistent across included studies. Four of the eighteen studies found an increased abundance of phylum Firmicutes together with a decrease in the abundance of Bacteroidetes in the new DM. At the genus/species level, a decrease in *Faecalibacterium prausnitzii*, *Roseburia*, *Dialister*, *Flavonifractor*, *Alistipes*, *Haemophilus*, and *Akkermansia muciniphila* and an increase in the abundance of *Lactobacillus*, *Streptococcus*, *Escherichia*, *Veillonella*, and *Collinsella* in the disease groups (preDM and newDM) were observed in at least two studies. In four studies, *Lactobacillus* was also found to correlate positively with fasting plasma glucose (FPG), HbA1c, and/or insulin resistance (HOMA-IR). This calls for further investigations on the species- or strain-specific role of the endogenously present *Lactobacillus* in the glucose regulation mechanism and in the progression of T2DM disease. Differences in dietary intake caused significant variations in the variety of bacterial species [77].

In conclusion, as regards the composition of the microbiota in patients with prediabetes, to date, the studies do not agree, but they do agree in stating that the intake of probiotics (multiple strains for at least 8 weeks) could be useful in the management of blood sugar metabolism in subjects with prediabetes.

Considering human studies, the scientific literature agrees in demonstrating that the intestinal microbiota plays an important role in regulating glucose metabolism. It was observed that the number of bacteria responsible for the production of short-chain fatty acids (SCFAs) was lower in patients with T2D. Decreased SCFA production leads to increased inflammation and can affect insulin secretion and pancreatic β-cell sensitivity, leading to insulin resistance. These data suggest that elevation of SCFAs, particularly butyrate, is important in glycemic control in prediabetes. Furthermore, a study conducted on animal models demonstrated how the intestinal microbiota protects against the development of obesity, metabolic syndrome, and prediabetes with an immune-mediated mechanism, inducing specific commensal Th17 cells. The gut microbiota influences host metabolic disorders through the modulation of several metabolites, such as the endotoxin LPS, BA, and TMAO, as well as SCFAs.

#### 4.3.1. Probiotics

Probiotics demonstrate promising potential in the management of prediabetes through their effects on insulin resistance and lipid profiles, as evidenced by both animal and human studies. The findings from animal models suggest that probiotics could serve as a complementary approach to lifestyle changes and pharmacotherapy in managing prediabetes. In the animal model, the following were studied in particular: lactobacilli and bifidobacteria.

Lactobacilli:

Within the species *L. plantarum*, several strains have successfully improved glycemic control and have anti-obesity effects in animals [88].

*L. rhamnosus* strains have improved glycemic control, lipid profile, and liver function in animal models [199,200,201].

*L. casei* strains 431, CCFM0412, and Shirota improved glucose tolerance in animals [202].

Several *L. paracasei* strains improved glycemic control in animals [203] or restored liver function in diet-induced hypercholesterolemic animals [204,205].

*L. gasseri* strains SBT2055 and BNR17 showed antidiabetic effects in high-fat-diet-fed mice [206].

Some *L. reuteri* strains led to a reduction in HbA1c and glucose levels in diabetic animals [207].

Bifidobacteria:

Within *B. animalis* ssp. lactis, strains 420 and I-2494 prevented weight gain and glucose intolerance in high-fat-diet-fed mice [208]. Strain 420 also reduced inflammatory cytokine levels and induced improved insulin sensitivity in this type of animal model [197].

As in the animal model, the following probiotics have also been studied in depth in humans: lactobacilli and bifidobacteria.

Lactobacilli:

A randomized, double-blind study conducted on 21 subjects (10 normal weight and 11 obese) aimed to verify whether the ingestion of probiotics could modify the intestinal microbiota and influence insulin resistance and the development of diabetes, hypothesizing that the daily intake of *Lactobacillus reuteri* increases insulin sensitivity by modifying the release of cytokines and insulin secretion itself through the modulation of the release of glucagon-like peptides (GLPs)-1 and -2. Participants received capsules containing placebo or 1010 *L. reuteri* cells to be ingested twice daily for 4 consecutive weeks. In glucose-tolerant volunteers, daily administration of *L. reuteri* SD5865 increased glucose-stimulated GLP-1 and GLP-2 release by 76% (*p* < 0.01) and 43% (*p* < 0.01), respectively, compared with placebo, along with 49% more insulin (*p* < 0.05) and 55% more C-peptide secretion (*p* < 0.05). However, the intervention did not alter peripheral and hepatic insulin sensitivity, body mass, ectopic fat content, or circulating cytokines. Thus, the study concluded that enrichment of the gut microbiota with *L. reuteri* increases insulin secretion, probably due to increased incretin release, but does not directly affect insulin sensitivity or body fat distribution. This suggests that oral ingestion of a specific strain may serve as a novel therapeutic approach to enhance glucose-dependent insulin release [80].

A 2016 review of randomized controlled trials (RCTs) suggested that prebiotics have a neutral effect on body weight, decreasing fasting and postprandial blood glucose and improving insulin sensitivity and lipid profile, while it appears that some inflammatory markers are decreased, sometimes substantially (20–30%). As for probiotics, these have shown significant but limited effects on body weight (<3%) and metabolic parameters. The effect was mainly observed with fermented milk or yogurt as opposed to administration in capsule form and with consumption for at least 8 weeks of multiple rather than single bacterial strains. Pickled and fermented foods, especially vegetables and legumes, could serve as a dietary source of pre-, pro-, and synbiotics. These foods have shown possible benefits with respect to morbidity and mortality in prospective cohort studies [87].

A 2017 review investigated the mechanisms employed by specific probiotic strains from the genera *Lactobacillus* and *Bifidobacterium*, which have shown efficacy in the treatment of obesity and T2D in both animal models and humans [88].

Probiotic strains with a positive impact on T2D and/or obesity are included in the genera *Bifidobacterium* and *Lactobacillus*, which are the most widely used [88].

Within the species *L. plantarum*, several strains have successfully improved glycemic control and had anti-obesity effects in subjects with T2D [81]. This randomized, double-blind, placebo-controlled study by M. Hariri et al. aimed to investigate the effects of probiotic (*L. plantarum* A7) supplemented soy milk compared to unsupplemented soy milk on MLH1 and MSH2 promoter methylation and oxidative stress among type 2 diabetic patients. Forty patients with type 2 diabetes mellitus aged 35 to 68 years were included and assigned to two groups. Patients in the intervention group consumed 200 mL/day of soy milk containing *Lactobacillus plantarum* A7, while those in the control group consumed 200 mL/day of conventional soy milk for 8 weeks. It was found that probiotic-supplemented soy milk significantly reduced promoter methylation in the proximal and distal MLH1 promoter regions (*p* < 0.01 and *p* < 0.0001, respectively) compared to baseline, while plasma 8-hydroxy-2′-deoxyguanosine (8-OHdG) concentration was significantly decreased compared to soy milk (*p* < 0.05). In addition, a significant increase in superoxide dismutase (SOD) activity was observed in the probiotic-supplemented soy milk group compared to baseline (*p* < 0.01). There were no significant changes from baseline in MSH2 promoter methylation in either group (*p* > 0.05). Consumption of probiotic soy milk improved antioxidant status in type 2 diabetic patients and may reduce promoter methylation among these patients, indicating that probiotic soy milk is a methylation agent among these patients and thus a promising agent for diabetes management.

*L. rhamnosus* strains improved glycemic control, lipid profile, and liver function in humans. The randomized, double-blind, placebo-controlled study by M. Sanchez et al. examined the impact of *Lactobacillus rhamnosus* CGMCC1.3724 (LPR) supplementation on weight loss and maintenance of weight loss in obese men and women for 24 weeks. A total of 125 subjects were included, aged 18 to 55 years, with a BMI of 29 to 41 kg/m^2^, without associated comorbidities (arterial hypertension, obstructive sleep apnea syndrome, type 2 diabetes, cardiovascular disease, family history of dyslipidemia). Each subject took two capsules per day of a placebo or an LPR formulation (1.6 × 108 colony-forming units of LPR/capsule with oligofructose and inulin). Each group was subjected to moderate energy restriction for the first 12 weeks, followed by 12 weeks of a maintenance diet. Body weight and body composition (measured by DEXA) were measured at baseline, week 12, and week 24. Data analysis showed that after the first 12 weeks and after 24 weeks, mean weight loss was not significantly different between the LPR and placebo groups when all subjects were considered. However, a significant treatment–gender interaction was observed. The mean weight loss in women in the LPR group was significantly higher than that in women in the placebo group after the first 12 weeks, whereas it was similar in men in both groups. Women in the LPR group continued to lose body weight and fat mass during the weight maintenance period, whereas opposite changes were observed in the placebo group. Changes in body weight and fat mass during the weight maintenance period were similar in men in both groups. LPR-induced weight loss in women was associated not only with significant reductions in fat mass and circulating leptin concentrations but also with the relative abundance of Lachnospiraceae bacteria in stool. The study shows that the *Lactobacillus rhamnosus* CGMCC1.3724 formulation could help obese women achieve sustained weight loss over time. 

*L. casei* Shirota reduced the effects of a high-fat diet (HFD) in healthy subjects [83]. The aim of the study by C.J. Hulston et al. was to determine whether probiotic supplementation with *Lactobacillus casei Shirota* (LcS) could prevent diet-induced insulin resistance in healthy subjects. A total of seventeen subjects were randomized to a probiotic group (n = 8) or a control group (n = 9). The probiotic group consumed a fermented milk drink with LcS twice daily for 4 weeks, while the control group received no supplementation. Subjects maintained their normal diet for the first 3 weeks of the study, after which they consumed a high-fat (65% energy) and high-energy (50% increase in energy intake) diet for 7 days. Insulin sensitivity was assessed by an oral glucose tolerance test conducted before and after refeeding. Body mass increased by 0.6 kg in the control group (*p* < 0.05) and by 0.3 kg in the probiotic group (*p* > 0.05). Fasting plasma glucose concentrations increased after 7 days of refeeding (control group: 5.3 vs. 5.6 mmol/l before and after refeeding, respectively, (*p* < 0.05), whereas fasting serum insulin concentrations were maintained in both groups. Glucose AUC values increased by 10% and insulin sensitivity decreased by 27% in the control group, whereas normal insulin sensitivity (calculated by the Matsuda insulin sensitivity index (ISI) obtained by a formula using plasma glucose and serum insulin concentrations obtained by OGTT) was maintained in the probiotic group before and after the food binge, respectively. These results suggest that probiotic supplementation may be useful in the prevention of diet-induced metabolic diseases such as type 2 diabetes.

*L. gasseri* SBT2055 strains showed promising anti-obesity effects when used in overweight adults. The study by Y. Kadooka et al. [84], a multicenter, double-blind, randomized, placebo-controlled intervention study, included 87 subjects with body mass index (BMI) between 24.2 and 30.7 kg/m^2^ and abdominal visceral adipose tissue between 81.2 and 178.5 cm^2^ (calculated by four-slice abdominal computed tomography scans at the level of lumbar vertebra 4–5), who were randomly assigned to receive fermented milk (FM) containing LG2055 (active FM; n = 43) or FM without LG2055 (control FM; n = 44) and were invited to consume 200 g/day of FM for 12 weeks. The results showed that in the active FM group, abdominal visceral and subcutaneous fat areas decreased significantly (*p* < 0.01) from baseline by an average of 4.6% and 3.3%, respectively. Body weight and other measures (BMI, waist, and hip circumference) also decreased significantly. In the control group, in contrast, none of these parameters decreased significantly. Serum high-molecular-weight adiponectin increased significantly (*p* < 0.01) in the active and control groups by 12.7% and 13.6%, respectively.

The study concluded that the probiotic LG2055 showed effects of reducing abdominal adiposity, body weight, and other measures, suggesting its beneficial influence on metabolic disorders [85].

The study by R.Mobini et al. has in fact demonstrated that the intake of *L. reuteri* DSM 17938 for 12 weeks, while not influencing HbA1c in patients with type 2 diabetes on insulin therapy, improved insulin sensitivity in a subset of participants and generally increased the high diversity of the intestinal microbiota.

The double-blind study randomized 46 people with type 2 diabetes to placebo or a low dose (108 CFU/day) or high dose (10^10^ CFU/day) of *L. reuteri* DSM 17938 for 12 weeks. Supplementation with *L. reuteri* DSM 17938 for 12 weeks did not affect HbA1c, hepatic steatosis, or microbiota composition. Participants who received the higher dose of *L. reuteri* showed increases in insulin sensitivity index (ISI) and serum deoxycholic acid (DCA) levels from baseline, but these differences were not significant in between-group analyses. Post hoc analyses showed that participants who responded with an increased ISI after *L. reuteri* supplementation had greater microbial diversity at baseline and increased serum DCA levels after supplementation. Furthermore, increases in DCA levels correlated with improved insulin sensitivity in probiotic recipients.

*L. acidophilus* NCFM preserved insulin sensitivity in both non-diabetic and diabetic subjects [86].

This conclusion was reached in the study by A.S. Andreasen et al. This randomized, double-blind study included forty-five males with type 2 diabetes, impaired or normal glucose tolerance, who were assigned to a 4-week treatment course of *L. acidophilus* NCFM or placebo. *L. acidophilus* was detected in stool samples by denaturing gradient gel electrophoresis and real-time PCR. *L. acidophilus* NCFM was detected in 75% of stool samples after treatment with the probiotic bacterium. Insulin sensitivity was preserved among volunteers in the *L. acidophilus* NCFM group, whereas it decreased in the placebo group. Both baseline inflammatory markers and the systemic inflammatory response were, however, unaffected by the intervention.

Bifidobacteria:

Within the genus *Bifidobacterium*, at least four species have shown significant effects on diabetes. Within the species *B. animalis* ssp. lactis, the strain HN019 induced a significant reduction in BMI, total cholesterol, low-density lipoprotein (LDL), and pro-inflammatory cytokines in patients with T2D [42]. In the study by Bernini et al., a randomized controlled trial, 51 patients with metabolic syndrome were included, divided into a control group (n = 25) and a probiotic group (n = 26). The probiotic group consumed 80 mL of probiotic-fermented milk (with 2.72 × 1010 colony-forming units of *B. lactis* HN019) over 45 days. Daily ingestion of fermented milk showed a significant reduction in body mass index (*p* = 0.017), total cholesterol (*p* = 0.009), and low-density lipoprotein (*p* = 0.008) compared to baseline and control values. In addition, a significant decrease in tumor necrosis factor-α (*p* = 0.033) and pro-inflammatory cytokine interleukin-6 (*p* = 0.044) was observed.

The above studies are included in the review by Hampe, C.S. et al., 2017, which analyzed the effect of individual probiotic strains on several metabolic parameters [88].

A 2022 meta-analysis that included seven publications for a total of 460 patients with prediabetes, however, demonstrated how probiotics were able to significantly reduce HbA1c levels, quantitative insulin sensitivity check index (QUICKI), total cholesterol, triglycerides, and LDL cholesterol compared to levels in the placebo group. While the effects on fasting blood glucose, HOMA index, and HDL cholesterol were not different from those of the placebo group. The meta-analysis therefore concluded that probiotics may play an important role in the regulation of HbA1c, QUICKI, total and LDL cholesterol, and TG in patients with prediabetes. Furthermore, based on existing studies, probiotics can regulate blood glucose homeostasis in various ways [90].

Regarding the duration of probiotic treatment, a 2016 meta-analysis suggested that the effect of probiotics on glucose metabolism was potentially greater when the duration of the intake was greater than 8 weeks or if multiple strains of probiotics were consumed simultaneously [92].

In summary, current evidence suggests that probiotic supplementation can improve certain glycemic and lipid parameters in prediabetes, such as HbA1c, QUICKI, TC, TG, and LDL-C.

Regarding the duration of probiotic treatment, a 2016 meta-analysis suggested that the effect of probiotics on glucose metabolism was potentially greater when the duration of the intake was greater than 8 weeks or if multiple strains of probiotics were consumed simultaneously.

The mechanisms by which probiotics may regulate blood glucose homeostasis in prediabetes include improving insulin resistance, regulating intestinal permeability, modulating metabolic regulation, and altering the gut microbiota composition.

Considering how long it takes to see improvements in blood sugar with probiotics, the studies suggest it may take a few months of consistent probiotic use before seeing significant, lasting improvements in blood glucose levels [92].

Specifically, a meta-analysis found that taking probiotics for 8 weeks or longer may enhance the effect on glucose metabolism [2].

The key is to keep probiotic intake as regular as possible and review trends in your blood glucose data daily and over time. Continuous glucose monitoring can provide ongoing data to assess the impact of probiotics on your blood sugars. However, it is important to note that probiotics should be considered one tool as part of an overall health strategy. Relying exclusively on probiotics to manage blood sugars is not recommended. One should continue to eat balanced meals, exercise regularly, and stay hydrated in addition to taking probiotics.

However, more research is needed to fully elucidate the mechanisms of action and establish optimal probiotic strains and dosages for prediabetes management.

#### 4.3.2. Symbiotics

Subsequent reviews have looked at the effect of probiotic or prebiotic supplementation or their combination, often reaching similar conclusions.

A 2021 systematic review including randomized controlled trials reports inconsistent results among included studies, partly due to limited sources. Prebiotics failed to show clear improvement in glycemic control, but their use led to changes in gut microbiota composition. The authors highlighted that probiotics can reduce glycated hemoglobin (HbA1c) and have the potential to improve blood glucose levels after oral glucose loading. This review included eight RCTs (for a total of 391 patients) of pro-, pre-, and synbiotics for the treatment of the prediabetic population. Probiotic supplementation can suppress the increase in blood cholesterol, but the improvement cannot be verified. A combination of probiotics and prebiotics through synbiotic supplementation is more effective than probiotics alone in glycemic control. The review concluded that the benefits of gut microbiota modulation in the treatment of prediabetes have been partially demonstrated. However, there is not enough evidence to show significant benefits on glucose metabolism, lipid metabolism, and body composition [89].

A 2022 systematic review that included 15 articles for a total of 1116 patients reached similar conclusions, stating that the administration of pre- and probiotics can provide beneficial and salutary effects in the clinical management of patients with prediabetes and metabolic syndrome. Different probiotic compositions have shown beneficial and remarkable effects on glucose homeostasis, lipid profiles, BMI, and inflammatory markers in subjects with prediabetes, metabolic syndrome, and healthy individuals and could be beneficial in rebalancing the gut microbiota in the prediabetic condition [91]. In this review, sometimes the types of pre- and probiotics administered in the various studies are not reported; when mentioned, they are probiotics of the *Lactobacillus* genus (*L. acidophilus*, *L. plantarum*, *L. reuteri*, *L. rhamnosus*, *L. bulgaricus*, *L. casei* strain Shirota, *L. paracasei*, *L. delbreuckii* subspecies bulgaricus, *L. salivarius* UBL S22, *L. gasseri*), of the genus *Bifidobacterium* (*B. lactis*, *B. longum*, *B. bifidum*, *B. brevis*, *B. animalis* sbsp. Lactis, *B. infantis*), or *S. thermophilus*, *Saccharomyces boulardii* individually or in various combinations. In other cases, a combination was administered: probiotic  +  B group vitamins, maltodextrin, lactose, and magnesium stearate. The administration time ranged from a minimum of 8 weeks to a maximum of 24, and the dosage was once daily.

### 4.4. Obesity

Obesity is a health problem whose incidence has increased dramatically in recent years in both developing and industrialized countries. The prevalence of obesity has reached pandemic proportions, with 13% of the adult population being obese in 2016 and 40% of the adult population being overweight [209].

Overweight and obesity rates continue to rise in both adults and children. From 1975 to 2016, the prevalence of overweight or obese children and adolescents aged 5 to 19 increased more than four-fold, from 4% to 18% globally. The global prevalence of obesity nearly tripled between 1975 and 2016 [210].

A BMI > 30 kg/m^2^ is a major health risk because it is related to the development of numerous pathologies, including cardiovascular diseases (mainly heart disease, dyslipidemia, stroke, and arterial hypertension); diabetes and insulin resistance; musculoskeletal disorders (especially osteoarthritis); neoplasms (the most common of which are endometrial, breast, ovarian, prostate, liver, gallbladder, renal, and colon cancer); non-alcoholic steatohepatitis; reproductive system disorders (including infertility in both sexes, low serum testosterone in men, and the development of polycystic ovary syndrome in women); and psychological disorders (depression and anxiety are the most common). The risk of these complications increases with increasing BMI. Childhood obesity is also associated with a greater likelihood of obesity, premature death, and disability in adulthood. In addition to an increased risk of adult disease, obese children experience breathing difficulties, increased risk of fractures, hypertension, early indicators of cardiovascular disease, insulin resistance, and psychological effects [211].

Research on the role of the gut microbiota in body weight regulation and energy metabolism has been rapidly expanding in recent years [212].

In healthy subjects, the gut microbiota is characterized by a high microbial diversity, with a prevalence of two major bacterial phyla: Bacteroidetes and Firmicutes. Obese people, on the contrary, tend to have a reduced microbial diversity, with a relative increase in Firmicutes and a decrease in Bacteroidetes [213]. Liu et al. in their 2021 review found correlations between the presence or absence of specific bacterial strains and obesity: the Firmicutes/Bacteroidetes ratio is a biomarker for obesity, but its relationship with specific types of adipose tissue varies. For example, Firmicutes abundance is positively correlated with markers of brown adipocytes in subcutaneous fat but not in visceral fat. Probiotics have been shown to reduce visceral obesity, suggesting a differential impact on fat distribution. Christensenellaceae are inversely correlated with BMI and are therefore associated with weight loss; *Akkermansia muciniphila* improves metabolic parameters and is associated with weight loss; for *Lactobacillus*, the effects are different depending on the species involved, where *L. paracasei* is negatively correlated with obesity, while *L. reuteri* and *L. gasseri* are positively correlated; *Bifidobacterium* has strain-dependent effects on obesity, where reduced abundance is associated with obesity [214].

There are multiple mechanisms by which microbiota disruption may impact weight gain, including increased absorption, inflammation, insulin resistance, altered lipid metabolism, and altered gut–brain axis [214].

1. Increased energy absorption: The review by Liu et al. (2021) suggested that gut dysbiosis may promote increased energy absorption from foods. This may be due to changes in microbial composition that affect the digestion process and absorption of nutrients, including carbohydrates and fats [214].

2. Inflammation and insulin resistance: Systemic inflammation mediated by the gut microbiota may contribute to insulin resistance and altered lipid and carbohydrate metabolism. The review by Islam et al. (2023) discussed how systemic inflammation may be an important mechanism underlying the microbiota–obesity relationship, influencing body weight regulation and the risk of developing metabolic conditions such as type 2 diabetes [215].

3. Regulation of lipid metabolism: Shelton et al. (2023) identified a specific metabolite produced by the microbiota during childhood (PLA, phenyllactic acid, produced by lactobacilli) that regulates intestinal lipid metabolism, thereby influencing body fat accumulation. This suggests that alterations in the production of microbial metabolites may contribute to the development of obesity [216].

4. Gut–brain axis: Gut dysbiosis may influence the communication between the gut and the brain, contributing to changes in feeding behavior and energy metabolism. The review by Asadi et al. (2022) [217] explored the role of the gut–brain axis in obesity and suggested that changes in microbial composition may influence feeding behavior and energy balance. The gut–brain axis, a bidirectional connection between the central nervous system and the intestine, plays a crucial role in controlling appetite and satiety. Gut microbes produce signaling molecules and neurotransmitters that influence appetite-regulating pathways in the brain. Several anorexigenic hormones (GLP-1, CCK, PYY) and orexigenic hormones (ghrelin) are involved in this process. Changes in microbiota composition may alter the signaling of these hormones and thus influence food intake and energy homeostasis [215].

5. Influence on reward pathways and mood: The gut microbiota alters mood and reward pathways, influencing high-calorie food intake. This was highlighted in the review by Cheng et al. of 2022 that showed how probiotics participate in the pathogenesis of obesity through multiple mechanisms, including disruption of energy homeostasis, increased lipid synthesis and storage, regulation of central appetite and feeding behavior, and triggering chronic low-grade inflammation [218].

These are just some of the mechanisms through which gut dysbiosis may contribute to weight gain. Importantly, the relationship between the microbiota and obesity is complex and multifactorial, involving a wide range of biological, environmental, and behavioral factors.

Preclinical animal studies have provided compelling evidence for the role of the gut microbiota in obesity. Experiments in germ-free mice, which lack gut microbiota, have shown that transplantation of microbiota from obese mice to lean mice can induce weight gain and metabolic alterations in recipient mice. This suggests that the microbiota can transmit obesogenic characteristics.

The hypothesis that the gut microbiota may be a relevant environmental factor in obesity has led to the study of the gut microbiota of obese individuals. The first evidence of a link between the gut microbiota and obesity came from studies in germ-free mice. The 2005 study by Ley RE et al. considered genetically obese mice (with a mutation in the ob gene encoding leptin) and lean mice (ob/+ and +/+) fed the same polysaccharide-rich diet and compared the microbiota composition of these two groups. Transplantation of gut microbes from conventionally raised mice into germ-free mice increased the fat content and insulin resistance levels of the transplanted mice even with reduced food intake, which demonstrated that gut microbes can increase adipose tissue accumulation in the host. Furthermore, 16S rRNA gene sequencing has shown that obesity can be associated with two dominant bacterial phyla: Firmicutes and Bacteroidetes. The gut microbiota of obese mice showed a 50% decrease in the abundance of Bacteroidetes and a proportional increase in Firmicutes [219].

The study by Bäckhed et al. in 2004 explored the role of the gut microbiota as an environmental factor that regulates fat storage in the body. Wild-type (WT) C57BL/6J mice and Rag1^−/−^ knockout mice maintained under germ-free (GF) and conventional (CONV-R) conditions were used. GF mice were colonized with gut microbiota collected from the cecum of CONV-R mice. Colonization of GF mice with a conventional microbiota resulted in a 60% increase in body fat content and insulin resistance within 14 days, despite a reduction in food intake. The microbiota promoted the absorption of monosaccharides from the intestinal lumen, inducing de novo hepatic lipogenesis. Fiaf (Fasting-Induced Adipocyte Factor), an inhibitor of lipoprotein lipase (LPL), was selectively suppressed in the intestinal epithelium of conventional mice, facilitating triglyceride deposition in adipocytes. LPL activity was increased in epididymal fat pads and hearts of conventional mice. Hepatic lipogenesis was increased with increased expression of lipogenic enzymes such as acetyl-CoA carboxylase (Acc1) and fatty acid synthase (Fas). The gut microbiota has been shown to act as an important environmental factor that influences energy harvesting from the diet and energy storage in the host. Suppression of Fiaf by the microbiota is essential for triglyceride deposition in adipocytes [220].

Turnbaugh et al. then confirmed that the Firmicutes/Bacteroidetes ratio was significantly increased in obese mice and demonstrated that the ability of the microbiota in obese mice to obtain energy from food was higher. In this study, they involved both genetically obese mouse models and obese and lean human volunteers, analyzing the differences in gut microbial composition and their impact on energy balance. In obese mice, the proportion of Firmicutes was significantly higher than Bacteroidetes. This trend was also confirmed in obese humans. The obesity-associated microbiota showed an increased capacity to ferment dietary polysaccharides, producing short-chain fatty acids such as butyrate and acetate, which were then absorbed and converted into complex lipids in the liver. Germ-free mice colonized with the obese microbiome showed a significant increase in body fat mass compared to those colonized with the lean microbiome, with no significant differences in food consumption [221].

The 2023 study by Li Z et al. demonstrates that altering the microbiota by administering specific peptides can modulate the microbiota in mouse models, thus having effects on the obesogenic phenotype, thus representing a possible future pharmacological strategy for the treatment of obesity. In this study, they administered a 9-amino-acid peptide called D3 orally to germ-free (GF) mice and wild-type (WT) mice, rats, and macaques. The effects of D3 on body weight and other basal metabolic parameters were evaluated. The effects of D3 on the gut microbiota were assessed using 16S rRNA amplicon sequencing. To identify and confirm the mechanisms of D3, ileal transcriptome analysis and molecular approaches were performed on three animal models. A significant reduction in body weight was observed in both WT (12%) and GF (9%) mice treated with D3. D3 improved leptin resistance and increased the expression of uroguanylin (UGN), which suppresses appetite through the UGN-GUCY2C endocrine axis. Similar effects were also found in diet-induced obese rat and macaque models. There was an obvious difference in fecal microbiota composition between D3-treated and vehicle-treated mice. Linear discriminant analysis showed that *Oscillospira*, *Lawsonia*, and *Desulfovibrio* were significantly enriched in vehicle-treated mice, while *Bacteroides*, *Bilophila*, and *Akkermansia* genera were significantly enriched in the D3-treated group. In addition, the abundance of intestinal *Akkermansia muciniphila* increased approximately 100-fold through the IFNγ-Irgm1 axis after D3 treatment, which may further inhibit fat absorption by downregulating Cd36. Peptide D3 represents a novel potential therapeutic to counteract diet-induced obesity by acting as a non-toxic bioactive peptide that can be taken orally. Modulation of the UGN-GUCY2C endocrine axis and upregulation of *A. muciniphila* in the gut microbiota are key mechanisms by which D3 exerts its anti-obesity effects [222].

The study by Shelton et al. examines how the gut microbiota in early life protects against environmentally induced obesity. Using a mouse model, the authors explore the effects of early exposure to antibiotics and a high-fat (HF) diet on obesity and intestinal lipid metabolism. They focus in particular on a *Lactobacillus*-derived metabolite, phenyllactic acid (PLA), which regulates peroxisome proliferator-activated receptor gamma (PPAR-g) in the gut epithelium to limit fat accumulation. Mice exposed early in life to antibiotics and an HF diet show significant increases in weight and adiposity compared to mice fed an HF diet alone. This increase in weight is not accompanied by increased food consumption, indicating that antibiotics amplify fat accumulation rather than caloric intake. Mice treated with antibiotics and an HF diet also show elevated fasting glucose and liver fat accumulation, signs of metabolic dysfunction. Even after cessation of antibiotic treatment, mice exposed early in life to antibiotics and an HF diet continue to show increased weight and adiposity, indicating long-lasting effects on metabolic function. Analysis of the gut microbiota revealed that exposure to antibiotics and an HF diet significantly reduces the presence of *Lactobacillus* in the small intestine. Experiments in gnotobiotic models confirmed that loss of *Lactobacillus* is a key factor in the increase in adiposity. Antibiotics and HF diet alter gene expression in the intestinal epithelium, particularly by reducing the levels of PPAR-g, a crucial regulator of lipid metabolism. Untargeted metabolomic analysis identified PLA as a *Lactobacillus*-derived metabolite that protects against antibiotic- and HF-induced metabolic dysfunction. PLA increases the abundance of PPAR-g in intestinal epithelial cells, thereby regulating lipid metabolism and preventing fat accumulation. The work of Shelton et al. therefore demonstrates that early antibiotic exposure in combination with a high-fat diet promotes obesity and metabolic dysfunction through altering the gut microbiota and regulating intestinal lipid metabolism. *Lactobacillus* and its metabolite, phenyllactic acid, play a crucial protective role by regulating PPAR-g expression in the intestinal epithelium. This highlights the importance of maintaining a healthy gut microbiota in early childhood to prevent obesity and other metabolic diseases [216].

Human research has confirmed the findings observed in animal studies, highlighting an altered composition of the gut microbiota in obese people compared to lean subjects. Obese people tend to have lower microbial diversity and an altered profile of bacterial species, with a predominance of bacteria from the phylum Firmicutes compared to Bacteroidetes.

As also reported in the 2018 review by Gomes et al [103]. published in *Gut Microbes*, GLP-1 (glucagon-like peptide-1) is also modulated by the gut microbiota and is responsible for controlling food intake and insulin secretion. The concentration of this hormone is lower in obese individuals compared to healthy individuals. Butyrate produced by gut bacteria is present in lower amounts in obese individuals and has been shown to regulate energy homeostasis by stimulating adipocytes to produce leptin and inducing GLP-1 secretion by L cells. In their 2012 study, Basseri et al. demonstrated the association between methanogenic archaea and the development of obesity. Using breath tests to measure the amount of methane emitted, they verified that subjects with detectable methane by a breath test had a significantly higher body mass index (BMI) compared to methane-negative controls. This implies a higher amount of *M. smithii* in obese individuals, which has not been observed in studies evaluating gut archaeal populations. It also suggests that methane may contribute to obesity by slowing intestinal transit and increasing the load of the gut microbiome. Although methane and constipation are correlated, methane remains an independent predictor of elevated BMI, implying that methane itself may have a direct impact on body weight (Basseri et al., 2012).

Therapies targeting the modulation of the gut microbiota represent a promising area of research for the prevention and treatment of obesity. Dietary interventions, such as the ketogenic diet, probiotics, prebiotics, and fecal microbiota transplantation, are potential strategies to modulate the microbiota.

Cuevas-Sierra et al. (2019) analyzed in a narrative review how diet, genetics, and epigenetics interact with the gut microbiota to influence obesity, highlighting the importance of an integrated approach that considers dietary and genetic factors. The authors suggest that diets rich in fiber and prebiotics may promote the growth of beneficial bacteria and improve energy metabolism. In particular, diet is a key factor in modulating the gut microbiota. High-fiber diets promote the production of SCFAs, which may have beneficial effects on metabolic health. High-fat diets, on the other hand, may alter the composition of the microbiota and increase intestinal permeability, contributing to inflammation and obesity. The authors review several novel approaches to modulate the gut microbiota and manage obesity, such as prebiotics, postbiotics, probiotics, and fecal microbiota transplantation, that represent promising approaches to modulate the gut microbiota and improve metabolic health. The gut microbiota can influence gene expression and induce epigenetic modifications, which may in turn influence the predisposition to obesity. SCFAs, for example, can modulate the activity of histone deacetylases and alter gene expression, with implications for health and the development of metabolic diseases [223].

#### 4.4.1. Probiotics

Asadi et al. in their 2022 narrative review examined the connection between the gut–brain axis and obesity, discussing how changes in the gut microbiota can influence feeding behavior and metabolism through modulation of the central nervous system. The article highlights the importance of considering the bidirectional interaction between the microbiota and the brain in the management of obesity, suggesting that probiotics may play a role in improving mood and reducing appetite [217].

Regarding the use of probiotics, several studies have evaluated their efficacy for the therapy of dysbiosis in obese patients.

In a double-blind, placebo-controlled clinical trial conducted by Parnell and Reimer in 2009, 48 healthy adults with a BMI > 25 kg/m^2^ were randomized to receive either oligofructose or a placebo daily for 12 weeks. Results showed that body weight decreased by 1.03 ± 0.43 kg with oligofructose supplementation, while the control group showed a weight gain of 0.45 ± 0.31 kg over 12 weeks (*p* = 0.01). Additionally, glucose increased in the control group and decreased in the oligofructose group between baseline and final tests (*p* ≤ 0.05). Oligofructose supplementation did not affect the secretion of active plasma glucagon-like peptide 1. According to a visual analog scale designed to assess side effects, oligofructose was well tolerated. A decrease in the area under the curve (AUC) of ghrelin and an increase in the AUC of PYY were also observed in the oligofructose-treated group. The oligofructose group reported a reduction in energy intake. Glucose levels decreased in the oligofructose group and increased in the control group. Insulin levels followed a similar pattern. The authors therefore concluded that oligofructose supplementation, independent of other lifestyle modifications, can promote weight loss and improve glucose regulation in overweight adults. The observed effects on ghrelin and PYY levels suggest that these hormones may contribute to a reduction in energy intake [96].

Later, in the study conducted by Larsen et al. in 2013, 50 obese patients were randomized to receive *Ligilactobacillus salivarius* (Ls-33) or a placebo for 12 weeks. Fecal microbiota and fecal SCFA concentrations were assessed before and after the intervention, focusing on the ratio of the *Bacteroides–Prevotella–Porphyromonas* group to *Firmicutes* bacteria and the probiotic *L. salivarius*. It was therefore seen that Ls-33 can influence the composition of the gut microbiota in obese adolescents, modifying the ratio of Bacteroidetes to Firmicutes, but these changes do not appear to be related to improvements in metabolic syndrome. The study suggests that while *L. salivarius* may have an impact on the microbiota, this effect may not translate into clear clinical benefits for metabolic syndrome in obese adolescents [94].

Another study conducted by Sharafedtinov et al. in 2013 on 40 obese adults who took *Lactiplantibacillus plantarum* for three weeks observed a reduction in BMI and blood pressure values. This pilot study suggests that a hypocaloric diet supplemented with probiotic cheese may contribute to a reduction in BMI and blood pressure in obese hypertensive patients, both recognized symptoms of metabolic syndrome. These results highlight the potential of probiotics to improve specific aspects of health in conditions of obesity and hypertension, but further studies with larger samples and longer follow-up periods are needed to confirm these findings [95].

Another 2013 study by Sarafavi et al. examined the impact of *L. casei*, *L. rhamnosus*, *S. thermophilus*, *B. breve*, *L. acidophilus*, *B. longum*, and *L. bulgaricus* combined with FOS in 70 children and adolescents with high BMI for eight weeks. The results suggested a reduction in BMI and waist circumference [97].

The 2013 randomized, double-blind clinical trial by Jung et al. examined the effects of the probiotic strain *Lactobacillus gasseri* BNR17 on overweight and obese adults. The study involved 62 obese volunteers aged 19 to 60 years with a body mass index (BMI) ≥ 23 kg/m^2^ and fasting glucose levels ≥ 100 mg/dL. The participants were divided into two groups: one received the probiotic BNR17 and the other a placebo for a period of 12 weeks. The results at the end of the 12 weeks showed a slight reduction in body weight in the group that received BNR17, but without statistically significant differences compared to the placebo group. However, a more marked decrease in waist and hip circumferences was observed in the BNR17 group compared to the placebo group. The probiotic BNR17 therefore showed some beneficial effects on weight and body circumference reduction, but without significant differences compared to the placebo [98].

In 2013, the research group of the University of Pavia led by Doria investigated through a randomized, double-blind, and placebo-controlled clinical trial the efficacy of a phytotherapeutic supplement containing phloridzin, isoflavones, and probiotics (Re-Code^®^) as an adjuvant in the reduction in body weight and fat mass in overweight women. The study involved 40 women between the ages of 30 and 54. These participants were divided into two groups: one received the herbal supplement, while the other received a placebo. The study participants followed a weight loss program that included a moderate low-calorie diet and moderate daily physical activity. The study showed that women taking the herbal supplement achieved a greater reduction in body weight, fat mass, and waist, thigh, and buttock circumference than the control group. In particular, phloridzin, a phytoestrogen similar to estradiol, showed positive effects on body fat reduction, especially in postmenopausal women. Isoflavones, mainly derived from Pueraria lobata, showed significant benefits in reducing fat mass and improving lipid profile [101].

Zarrati et al. in 2014 conducted a study on 75 healthy overweight and obese individuals randomized to receive *L. acidophilus* La5, *Lacticaseibacillus casei* DN001, *B. lactis* Bb12, or a placebo for eight weeks. These strains altered gene expression in peripheral blood mononuclear cells and altered BMI, percent fat, and LEP levels [99].

In 2014, Sanchez et al. examined the impact of a combination of *L. rhamnosus* CGMCC1.3724 (LPR) with oligofructose and inulin on a sample of obese men and women for 36 weeks [82]. The study involved a total of 125 obese subjects, randomized and subjected to a reduced-calorie diet for 12 weeks, followed by a weight maintenance period of another 12 weeks. Participants received two capsules per day containing LPR or a placebo. The objectives were to evaluate the effect of LPR on weight loss and weight maintenance. Women taking LPR lost significantly more weight than those in the placebo group after the first 12 weeks and continued to lose weight during the maintenance period, while no significant difference in weight loss was observed between men in the two groups. In women, weight loss was accompanied by a reduction in fat mass and blood leptin levels, while in men no significant differences were detected. In women, supplementation with LPR resulted in a higher relative abundance of bacteria from the Lachnospiraceae family in stool, suggesting a possible role of the microbiota in weight loss. The study therefore concluded that supplementation with *Lactobacillus rhamnosus* CGMCC1.3724 may facilitate sustainable weight loss in obese women but does not appear to have the same effect in men. LPR-induced weight loss in women has been associated with changes in the gut microbiota, highlighting the potential role of these probiotics in weight management.

Probiotics, especially various *Lactobacillus* strains, may be effective in reducing body weight and fat mass in overweight subjects. However, the effects are strain-dependent and may vary based on diet and other associated factors. Various reviews have investigated this association over the years.

The 2017 systematic review by Crovsey et al. investigated which bacterial strains were associated with weight loss. They examined various *Lactobacillus* strains, including *L. gasseri*, *L. plantarum*, *L. rhamnosus*, *L. curvatus*, *L. acidophilus*, and *L. casei*, often in combination with low-calorie diets or other phenolic compounds. Randomized controlled trials in overweight or obese adults that assessed body weight and/or fat mass following *Lactobacillus* intervention were included. From the 1567 initially identified, 14 articles were selected, of which 9 showed a reduction in body weight and/or fat mass, 3 found no significant effect, and 2 reported weight gain. Studies that reported beneficial effects on obesity used various *Lactobacillus* species, including *L. gasseri*, *L. plantarum*, *L. rhamnosus*, *L. acidophilus*, and *L. amylovorus*. In particular, efficacy appears to be strain-dependent, with combinations of *Lactobacillus* with a hypocaloric diet or other components (such as phenolic compounds) showing the best results. On the other hand, some studies reported weight gain, highlighting the need for further research to better understand these effects. The review suggests that probiotics, particularly certain *Lactobacillus* species, may have beneficial effects on weight loss in overweight individuals, but these effects vary by strain and specific study conditions. Limitations were also highlighted, such as the lack of control of caloric intake in some studies, which may influence the results. *Lactobacillus* may be a potential adjunct in the treatment of obesity, but efficacy depends on the strain used and the treatment conditions. Further studies are needed to clarify the precise role of these probiotics in body weight management [104].

In 2017, with a systematic review and meta-analysis, Borgeraas et al. examined the effect of probiotic supplementation on various obesity-associated parameters: body weight, body mass index (BMI), fat mass, and body fat percentage. It was conducted to evaluate whether probiotics could be an effective strategy for reducing weight or body fat in individuals with overweight or obesity. Fifteen randomized controlled trials (RCTs) were selected, including a total of 957 participants with an average BMI of 27.6 kg/m^2^, and a majority of female participants (63%). Data sources included MEDLINE, EMBASE, and the Cochrane Central Register of Controlled Trials, covering studies published from 1946 to 2016. The analysis used a random-effect model to compare probiotic groups to control (placebo) groups. The duration of interventions ranged from 3 to 12 weeks. The findings indicated that probiotic use led to a significant reduction in certain anthropometric parameters—body weight (−0.60 kg), BMI (−0.27 kg/m^2^), and body fat percentage (−0.60%)—compared to the placebo group. However, the reduction in fat mass was not statistically significant. Results showed variability across studies, influenced by the number of probiotic strains used and treatment duration. Interventions with a single strain and durations exceeding 8 weeks tended to show greater effectiveness in weight reduction. The authors further explore the connection between gut microbiota and obesity, observing that microbes in obese individuals exhibit increased efficiency in extracting energy from food, potentially contributing to weight gain. Consequently, manipulating the microbiota through probiotics is considered a potential strategy for obesity management, though further studies are needed to clarify the specific mechanisms through which probiotics may influence weight [105].

Geng et al. in their 2022 review explored the links between gut microbiota and obesity-related diseases, such as diabetes and cardiovascular disease, highlighting the therapeutic potential of prebiotics and probiotics in reducing systemic inflammation and improving insulin sensitivity. The study suggests that the combined use of probiotics and prebiotics could represent a synergistic approach for the management of obesity and its complications. In particular, *Lactobacillus* strains can reduce body fat accumulation, improve insulin sensitivity, and reduce inflammation, while *Bifidobacterium* supplementation has been associated with improvements in lipid and glucose metabolism, as well as an increase in the production of beneficial SCFAs [102].

The 2024 article by Musazadeh et al. investigates the effects of probiotic supplementation on obesity through an umbrella meta-analysis. A total of 29 meta-analyses, including data from 14,366 participants, were evaluated to assess the influence of probiotics on three key obesity indices: body mass index (BMI), body weight (BW), and waist circumference (WC). The study found that probiotics led to significant reductions in BMI, BW, and WC. Specifically, greater effects on body weight were observed when interventions lasted over 8 weeks and in obese individuals, while BMI improvements were more pronounced in participants with metabolic syndrome or in treatments lasting 12 weeks or more.

The analysis used a random-effect model with sensitivity and subgroup analyses to account for study heterogeneity. The findings indicate that probiotic supplementation could serve as an effective intervention for managing obesity by significantly reducing body weight, BMI, and waist circumference [107].

A further meta-analysis was published in 2024 by Ning Cao et al. This study included 11 randomized controlled trials (RCTs) that evaluated the effectiveness of probiotic consumption in weight loss and improvements in glucose and lipid metabolism in overweight or obese women. The studies were sourced from databases like PubMed, EMBASE, and Cochrane Library until March 2024. The results showed that probiotic intake significantly reduced waist circumference (WC), insulin levels, and LDL cholesterol in overweight or obese women. However, there were no significant effects on body weight reduction, body mass index (BMI), or fat mass. The positive effects were more pronounced in studies with longer intervention durations and in those that included dietary or exercise interventions. The analysis also revealed moderate heterogeneity among the included studies, with results influenced by factors like the duration of the intervention. Notably, the effect on LDL cholesterol was positive in both short- and long-term studies. WC reduction was significant even with shorter interventions. Finally, sensitivity analysis confirmed that removing individual studies did not alter the overall meta-analysis results [106].

In conclusion, the role of the gut microbiota in the development and management of obesity is emerging as a crucial field of research. Scientific evidence shows that obese people tend to present a reduced microbial diversity and an increased Firmicutes/Bacteroidetes ratio, with a greater capacity to extract energy from the diet. Preclinical and clinical studies have shown that specific microbial strains, such as *Akkermansia muciniphila*, *Christensenellaceae*, and some *Lactobacillus*, can influence energy metabolism and contribute to the regulation of body weight.

Randomized clinical trials (RCTs) in humans are still limited (currently 10 studies), but have explored different probiotic and synbiotic strains. Of these, four studies have evaluated the efficacy of single strains, such as *Lactobacillus rhamnosus*, *Lactobacillus gasseri*, *Bifidobacterium breve*, and *Lactobacillus plantarum*. Another three studies have investigated combinations of strains, such as *Lactobacillus rhamnosus* with *Bifidobacterium longum* and *Lactobacillus bulgaricus* with *Streptococcus thermophilus*. Finally, three studies have considered the efficacy of synbiotics that combine prebiotics such as fructo-oligosaccharides (FOSs) with probiotics, showing promising results in improving the metabolic profile and reducing adiposity.

Despite the encouraging results, research suggests that the efficacy of probiotics in the treatment of obesity is strain-dependent and varies according to individual conditions and diet. Microbiota modulation strategies, including probiotics, prebiotics, and fecal transplantation, represent a potential therapeutic for the management of obesity and its complications. However, further studies are needed to confirm the efficacy of these strategies on a large scale and better understand the mechanisms involved.

#### 4.4.2. Symbiotics

In 2013, a study by Sarafavi et al. examined the impact of *L. casei*, *L. rhamnosus*, *S. thermophilus*, *B. breve*, *L. acidophilus*, *B. longum*, and *L. bulgaricus* combined with FOS in 70 children and adolescents with high BMI for eight weeks. The results suggested a reduction in BMI and waist circumference [97].

In a randomized controlled clinical trial on 77 obese children, Ipar et al. in 2015 suggested some promising effects of *L. acidophilus*, *L. rhamnosus*, *B. bifidum*, *B. longum*, and *E. faecium* in combination with FOS. The tested supplement had an effective impact on anthropometric measures and could reduce TC, LDL-C, and serum total oxidative stress levels [100].

### 4.5. Hyperhomocysteinemia

Homocysteine is a sulfur-containing compound obtained by eliminating a methyl residue from the amino acid methionine. Under physiological conditions, homocysteine is converted into methionine or cysteine thanks to the presence of B vitamins that act as cofactors with the enzymes methionine synthase and cystathionine synthase. Excessive consumption of methionine associated with a reduced intake of vitamins B6, B9 (folic acid), and B12 leads to plasma accumulation of homocysteine, or hyperhomocysteinemia (HHcy), which is a metabolic condition recognized as an independent risk factor for various significant medical conditions, such as cardiovascular events, kidney disease, and neuropsychiatric abnormalities [224].

Folic acid is the most important dietary determinant of homocysteine, and folic acid supplementation should be recommended to any patient who has an elevated Hcy level [108].

In the scientific literature, there are studies that demonstrate a correlation between hyperhomocysteinemia, induced by a high-methionine and low-folate diet, and the composition of the microbiota. In the study by Cheng et al. in 2016 [225], the composition of the microbiota was compared in two groups of mice: one with a standard diet, and one with a high-methionine and low-folate diet to induce hyperhomocysteinemia. A diet rich in methionine and low in folic acid (HMLF) led to glucose intolerance and insulin resistance and to an alteration of the intestinal microbiota. To compare the two groups of mice, 16S RNA sequencing of the V4 region was used. In the HMLF group, an increase in Bacteroides and a reduction in Proteobacteria were demonstrated. Among the quantitatively less represented phyla, there was an increase in *Tyzzerella*, *Odoribacter*, and *Allobaculum*, whereas there was a decrease in *Roseburia* and *Romboutsia*. The families *Porphyromonadaceae* and *Erysipelotrichaceae* were increased in the HMLF group.

Most bifidobacteria, except *B. gallicum* and biavatii, possess the gene responsible for folic acid synthesis [226], while most lactate-producing bacteria cannot synthesize folate. In fact, the only strain capable of synthesizing folic acid is *Lactobacillus plantarum* [227]. According to the study by Strozzi et al. [109], supplementation with *Bifidobacterium* adolescentis and pseudocatenulatum resulted in increased fecal folate levels. Lactobacilli and bifidobacteria, which are found in fermented dairy products, can synthesize vitamins de novo, particularly B vitamins such as folic acid and vitamin B12.

In the study by Li et al. [228], the relationship between gut microbiota and hyperhomocysteinemia induced by a methionine-rich diet was studied in a population of rats. It was investigated whether the reduction in the host microbiota could reduce plasma homocysteine. In this context, microbiota reduction means a reduction in the number of bacteria present in the gastrointestinal tract (essentially in the colon) following antibiotic therapy. Antibiotic therapy is in fact able to change the balance at the level of the intestinal microbiota ecosystem as it acts on the vital metabolism of bacteria. The results showed that the use of antibiotics capable of reducing the intestinal microbiota reduced plasma homocysteine levels and reduced glucose intolerance. The methionine-rich diet increased methionine levels in the intestinal epithelium, while the use of antibiotics reduced them. The alteration of the microbiota induced by the methionine-rich diet was mainly due to strains of *Faecalibaculum* and *Dubosiella*, producers of homocysteine, and therefore directly related to plasma homocysteine levels. In HM-treated mice, *Erysipelotrichaceae* bacteria increased and *Rikenellaceae* bacteria decreased. Homocysteine levels were detected by LC—MS (liquid chromatography–tandem mass spectrometry) from the culture supernatant of *Dubosiella newyorkensis*.

A study by Riedijk et al. [229] in 2007 highlighted the role of the gastrointestinal tract in homocysteine production in pigs. Using an isotope labeling method, it was found that 20% of the methionine introduced with the diet is metabolized by the GI, 31% is metabolized into homocysteine, 29% into tissue proteins, and 40% into CO2. However, the role of the gastrointestinal tract still requires in-depth studies. In the study by Zinno et al. [230], the impact of supplementation of dairy matrices containing folates on the microbiota and plasma levels of hyperhomocysteinemia was studied on a population of hyperhomocysteinemic mice: forty mice were divided into six groups, each group consisting of six to eight mice. The first two groups were fed a standard control diet (C) and a folate-deficient diet (FD), respectively, for 10 weeks. The other four groups were initially fed a folate-deficient diet (FD) for the first 5 weeks, then switched to a standard diet (group R: repleted), or a diet supplemented with folate-fermented milk (FFM), fermented milk (FM), or non-fermented milk (M: milk).

Preliminary analysis performed on mice fed with diets C and FD showed that homocysteine levels were significantly increased (about double, *p*  <  0.05) after 5 weeks of the FD diet compared to C. At the end of the treatments (10 weeks), supplementation with diet R as well as with the three dairy matrices (FM, FFM, and M) resulted in a significant reduction in plasma homocysteine levels, which reached values similar to those observed in group C. On the other hand, mice fed with the FD diet for 10 weeks showed a significant, much more pronounced increase (about 6.5-fold increase) in plasma homocysteine levels compared to group C (*p* <  0.05). Taxonomic assignments at the phylum level showed a homogeneous bacterial composition within the groups, although a slight interindividual variability was observed. Total DNA was extracted from 80 mg stool samples collected at the end of treatment with the QIAamp DNA Stool Mini Kit (sourced from QIAGEN, Hilden, Germany.). *Actinobacteria*, *Bacteroidetes*, *Deferribacteres*, *Firmicutes*, and *Verrucomicrobia* were detected as the predominant bacterial phyla, with different relative proportions in each supplementation group. Firmicutes was the most abundant phylum in all groups, with a relative abundance ranging from 58 to 94% across samples. On the pooled genus data by LEfSe analysis, specific metagenomic biomarkers could be detected. The control group was characterized by *Aldercreutzia* and *Staphylococcus*, while FD mice were dominated by *Eubacterium*, *Coprobacillus*, and *Clostridium*. Two additional bacterial groups were identified, namely *Erysipelotrichaceae* and *Clostridiaceae*. The R group was characterized by *Ruminococcus*, *Turicibacter*, and *Mogibacteriaceae*, while *Oscillospira*, *Coprococcus*, and *Clostridiales* characterized the M group rats. The FM and FFM fermented milk-supplemented groups of mice were dominated by *Lactobacillus*, *Holdemania*, *Ruminococcaceae*, *Parabacteroides*, *Dehalobacterium*, *Streptococcus*, and *Lachnospiraceae*, respectively. Interestingly, one of the biomarker genera associated with FFM was Streptococcus, which was exclusively present in this group, while *Lactobacillus* was one of the specific biomarkers of the FM group. Although not direct proof, these results suggest the possibility that the bacterial strains used for fermentation could be able to reach the gastrointestinal tract of treated mice. Overall, the results obtained from the study represent a starting point for the applicability of folate-enriched matrices for the management of hyperhomocysteinemia.

In conclusion, the presence of an alteration in the composition of the microbiota in animals with hyperhomocysteinemia has been demonstrated in the animal model. The diet rich in fats and methionine induces an increase in plasma homocysteine. This alteration of the microbiota can be counteracted with a correct diet and with some probiotic bacterial strains such as *Bifidobacteria* and *Lactobacilli*.

With regard to studies on humans, the study by Molnar et al. [231] from 2020 is interesting, in which a relationship was sought between diet, microbiota, hyperhomocysteinemia, and hidradenitis suppurativa in humans, a skin disease characterized by chronic inflammation and painful skin nodules that tend toward suppuration and transformation into scar tissue that also involves the hypodermic layer. A high-fat diet causes an increase in the Firmicutes/Bacteroidetes ratio, a reduction in antimicrobial peptides, and an increase in pro-inflammatory cytokines in the gastrointestinal epithelium. The increase in TNF alpha increases the gene expression of metalloproteinases, in particular isoforms 2, 8, and 9, which induce increased remodeling of the extracellular matrix, worsening the underlying disease. There is therefore a relationship between diet, microbiota, inflammation, plasma homocysteine, and severity of hidradenitis suppurativa.

#### Probiotics

Studies conducted on humans regarding the intake of probiotics have shown that the use of *B. adolescentis* and *B. pseudocatenulatum* in men increases the fecal concentration of folic acid [109]. The study was conducted on 23 healthy volunteers who took 5 × 10^9^ colony-forming units/day of these two strains. In the study, there was an initial observation period of 30 days followed by an actual treatment period with probiotics for 30 days. The fecal concentration of folic acid was measured before the administration of the probiotic and 48 h after the ingestion of the probiotic to evaluate the levels of fecal folic acid. *B. adolescentis* and *B. pseudocatenulatum* can therefore be an endogenous source of folic acid and exert a trophic function on the enterocytes of the colon mucosa, and unlike oral supplements, they can guarantee constant bioavailability.

Moreover, recent studies have explored the relationship between hyperhomocysteinemia (HHcy) and probiotics, indicating that probiotics may play a beneficial role in managing elevated homocysteine levels. A randomized double-blind placebo-controlled trial investigated the effects of a multispecies probiotic (in an amount of 2.5 × 10^9^ CFU/g: *Bifidobacterium bifidum* W23, *Bifidobacterium lactis* W51, *Bifidobacterium lactis* W52, *Lactobacillus acidophilus* W37, *Lactobacillus brevis* W63, *Lactobacillus casei* W56, *Lactobacillus salivarius* W24, *Lactococcus lactis* W19, and *Lactococcus lactis* W58) on homocysteine levels in obese women. Over a 12-week period, participants taking the probiotic showed significant reductions in homocysteine concentrations, along with improvements in oxidative stress markers and lipid profiles. Specifically, reductions in total cholesterol, low-density lipoprotein (LDL), and triglycerides were noted, suggesting that probiotics may help mitigate cardiometabolic risks associated with HHcy [110].

In conclusion, probiotics may influence homocysteine metabolism by enhancing the production of B vitamins, which are crucial for homocysteine breakdown. Certain probiotic strains have been shown to produce vitamins, such as folate and vitamin B12, which are directly involved in the metabolism of homocysteine, potentially leading to lower plasma levels of this amino acid. Moreover, probiotics help restore a balanced gut microbiota, which can be disrupted in conditions like obesity and metabolic syndrome. This dysbiosis may contribute to increased homocysteine levels. By re-establishing a healthy microbial community, probiotics can improve metabolic processes, including those involved in homocysteine metabolism. Probiotic supplementation has been associated with decreased levels of inflammatory markers and oxidative stress, both of which can exacerbate homocysteine levels. For instance, a study indicated that multispecies probiotics reduced homocysteine levels while also lowering tumor necrosis factor-alpha (TNF-α) and improving total antioxidant status in obese women. This suggests that probiotics may mitigate the inflammatory processes that can lead to elevated homocysteine.

In addition, probiotics have been shown to improve lipid profiles by reducing total cholesterol and triglyceride levels, which are often associated with cardiovascular risk. This improvement in lipid metabolism may indirectly influence homocysteine levels, as lipid abnormalities can be linked to dysregulated homocysteine metabolism.

### 4.6. Dyslipidemia

Dyslipidemia is a metabolic disease with a complex pathogenesis that can cause atherosclerosis, coronary heart disease, stroke, and other cardio-cerebrovascular diseases. A growing number of studies have shown how alterations in the microbiota are strongly associated with the development of dyslipidemia and that a high-fat diet can modify the intestinal environment on which the intestinal microbiota depends. A review by Lei L. et al. [232] including 288 studies has highlighted how, when the intestinal environment changes, the growth of normal microbiota such as *Bifidobacterium, Lactobacillus*, and butyric acid-producing bacteria is inhibited, combined with an increase in enterobacteria that become dysregulated, giving rise to dyslipidemia. In turn, dyslipidemia further aggravates the dysbiosis of the intestinal microbiota. More specifically, a large number of nutrients, such as polyunsaturated fatty acids (PUFAs), fiber, carnitine, and bile acids, are metabolized by bacterial enzymes to produce short-chain fatty acids (SCFAs), conjugated linoleic acid (CLA), TMAO, and secondary bile acids (BAs); TMAO reduces HDL cholesterol levels; SCFAs and CLA interact with peroxisome proliferator-activated receptors (PPARs) to reduce HDL, TG, and VLDL levels. The intestinal microbiota also regulates the lipid profile through the production of cholesterol oxidase in order to inhibit cholesterol synthesis and promote its degradation and transformation. In turn, it has been shown that a diet rich in lipids can cause an increase in Firmicutes and Bacteroidetes in the colon, the main phyla that influence blood levels of TG and HDL cholesterol. Some authors have used a two-sample Mendelian randomization (MR) analysis with the inverse-variance weighted (IVW) method to study the causal association between gut microbiota and different types of dyslipidemia in humans and the subsequent impact of the microbiota on lipid metabolism in order to identify evidence supporting the use of gut microbiota modulation as a strategy for dyslipidemia management. For example, by using this in-depth analysis method, Zhou et al. examined the genetic variants that had a statistically significant association with 129 distinct genera of gut microorganisms and their possible link to different types of dyslipidemia. The results highlighted a possible causal association between 22 genera of gut microbiota and dyslipidemia in humans. Furthermore, these findings indicated that the influence of gut microbiota on the regulation of dyslipidemia depends on the phylum, family, and specific genus. The phylum *Bacillota* showed the greatest diversity, with fifteen distinct genera distributed among eight families. In particular, gut microbiota derived from the families *Lachnospiraceae* and *Lactobacillaceae* showed statistically significant associations with lipid levels that contribute to overall health (*p* < 0.05); these families should therefore be recognized as probiotics that can significantly contribute to the regulation of lipid metabolism [113]. Some studies have analyzed how the composition and function of the gut microbiota are influenced by the properties of the diet, in particular the amount and composition of lipids, and, simultaneously, how dietary lipids can in turn influence the physiology of the host through the interaction with the gut microbiota. Lipids in fact act on the microbiota both as substrates for bacterial metabolic processes and by inhibiting bacterial growth through a toxic action. Furthermore, diseases related to an alteration of lipid metabolism, such as non-alcoholic liver disease and atherosclerosis, are associated with changes in the gut microbiota profile. The influence of the gut microbiota on the host lipid metabolism can be mediated, as previously mentioned, by metabolites produced by the gut microbiota itself, such as short-chain fatty acids (SCFAs), secondary bile acids, and trimethylamine, and by pro-inflammatory factors of bacterial origin, such as lipopolysaccharide [233].

For example, to assess how dietary fat sources may influence the microbiota, Caesar et al. compared mice fed a high-saturated-fat diet containing lard with mice fed an isocaloric high-fat diet containing fish oil, rich in n-3 PUFA, for 11 weeks. The composition of the gut microbiota was analyzed by pyrosequencing the 16S rRNA gene in the cecal contents of these mice. First, mice fed lard gained weight, consumed more food, and had higher feed efficiency than mice fed fish oil. Mice fed lard had a reduced respiratory quotient, indicative of increased fat utilization, both after 2 days and after 5 weeks of the high-fat diet. They also showed increased Toll-like receptor (TLR) activation and WAT inflammation and decreased insulin sensitivity compared to fish oil-fed mice. Finally, it was found that the genera *Bacteroides*, *Turicibacter*, and *Bilophila* were increased in lard-fed mice, while beneficial bacteria such as Actinobacteria (*Bifidobacterium* and *Adlercreutzia*), lactic acid bacteria (*Lactobacillus* and *Streptococcus*), *Verrucomicrobia* (*Akkermansia muciniphila*), *Alphaproteobacteria*, and *Deltaproteobacteria* were increased in fish oil-fed mice [234].

Another example can be the study by Devkota et al., who demonstrated, in a mouse model, that the consumption of a diet high in saturated fats (derived from milk) but not in polyunsaturated fats (safflower oil) modified the conditions of microbial assembly and promoted the expansion of a low-abundance and sulfite-reducing pathobiont, *Bilophila wadsworthia*, difficult to detect in healthy individuals but which emerges in pathological conditions such as appendicitis and other intestinal inflammatory disorders. In fact, milk lipids promote the taurination of bile acids, which increases the availability of organic sulfur used by sulfite-reducing microorganisms such as *B. wadsworthia*; increased levels of *B. wadsworthia* were in turn associated with a pro-inflammatory T-helper type 1 immune response and an increased incidence of colitis in subjects genetically susceptible to Il10. The high-fat diets used in this study were isocaloric and differed only in the type of dietary fat, which was held constant at 37% of total calories, closely mimicking Western consumption. Twenty-one-day exposure to the study diets resulted in significant differences in the structure of the enteric microbiota, which was analyzed by Sanger-based DNA sequencing and 454 pyrosequencing of 16S rRNA genes from cecal contents and feces. Together, these data demonstrate that dietary fat, by promoting changes in host bile acid composition, can markedly alter the conditions of the intestinal microbial assemblage, resulting in dysbiosis that can perturb immune homeostasis [235].

A study by Velagapudi et al., investigating the effect of gut microbiota on host energy and lipid metabolism, compared the serum metabolome and lipidomes of serum, adipose tissue, and liver of conventionally bred (CONV-R) and germ-free (GF) mice. Male GF mice (aged 12–14 weeks) were maintained in flexible plastic film isolators under a 12 h light cycle. Sterility was confirmed by culture and PCR analysis of feces using primers that amplify the 16S rRNA gene. Age-matched male CONV-R mice were transferred to identical isolators at weaning. Blood was collected from the vena cava under deep isoflurane anesthesia after a 4 h fast. The liver and epididymal white adipose tissues were removed and frozen in liquid nitrogen. The analysis showed that the serum metabolome of CONV-R mice was characterized by increased levels of energy metabolites, such as pyruvic acid, citric acid, fumaric acid, and malic acid, compared to GF mice, consistent with a higher energy metabolism in the presence of gut microbiota. At the same time, a reduction in serum triglyceride levels and an increase in liver triglyceride levels and adiposity were found, consistent with an increase in lipid clearance. A significant influence of the microbiota on phosphatidylcholine species was also observed, with increased levels in the serum and liver of CONV-R mice. In light of this, it was demonstrated that the gut microbiota increases energy metabolism and has systemic effects on host lipid metabolism, particularly on triglycerides and phosphatidylcholines, with a role in the development of metabolic diseases. Furthermore, conventionalization with specific bacterial strains, such as *Lactobacillus* spp. that upregulate genes associated with lipid metabolism, has been shown to increase lipid absorption. These results, correlated with other studies, demonstrate that GF mice have upregulated cholesterol biosynthesis genes, increased lipid excretion, and increased insulin sensitivity in response to high-fat-diet feeding, all of which contribute to the altered lipid metabolism observed in these mice [236].

Regarding studies in humans, the association between the composition of the microbiota and physiopathological conditions associated with dyslipidemia and ectopic fat deposition, such as atherosclerosis and hepatic steatosis, was studied in the study by Koren et al. In particular, the microbial composition of atherosclerotic plaques was analyzed to test the hypothesis that the oral or intestinal microbiota may contribute to atherosclerosis in humans; 454 pyrosequencing of 16S rRNA genes was used to examine the bacterial diversity of atherosclerotic plaques and oral and intestinal samples from 15 patients with atherosclerosis, and oral and intestinal samples from age- and sex-matched healthy controls. Shared operational taxonomic units (OTUs) were observed across all three sites (oral, intestinal, and atherosclerotic plaques), consistent with the possibility that the oral and gastrointestinal microbiota may be involved in the inflammatory processes responsible for atherosclerosis and that the atherosclerotic plaque microbiota may derive at least in part from the oral cavity and/or the intestine. Additionally, several shared OTUs were found between the atherosclerotic plaque and the intestine, suggesting that bacteria present in the atherosclerotic plaque may also derive from the distal intestine and the oral cavity. One mechanism by which bacteria might reach atherosclerotic plaque is phagocytosis by macrophages in epithelial linings; after phagocytosis, macrophages become activated and, upon reaching the activated endothelium of the atheroma, leave the bloodstream to enter the atheroma and transform into cholesterol-laden foam cells. In addition to shared OTUs between atherosclerotic plaques and oral/gut microbiota, this analysis revealed that specific components of the oral and gut microbiota correlated with disease markers: Streptococcus was strongly positively correlated with HDL cholesterol; Fusobacterium was positively correlated with LDL cholesterol and total cholesterol. Similarly, members of the Erysipelotrichaceae and Lachnospiraceae families in the gut were also positively correlated with LDL cholesterol and total cholesterol [112].

In a further study, Cortillard et al. conducted diet-induced weight loss and weight stabilization interventions in a study sample of 38 obese and 11 overweight individuals to investigate the temporal relationships between food intake, gut microbiota, and metabolic and inflammatory phenotypes. Subjects followed a hypocaloric high-protein diet for 6 weeks, followed by a maintenance diet for an additional 6 weeks. A 35% reduction in energy intake after the first 6 weeks was associated with a reduction in body fat mass, adipocyte diameter, and improvements in insulin sensitivity and markers of metabolism and inflammation. Quantitative metagenomic analysis of the gut microbiome of these subjects revealed a high percentage of individuals (23–40%) with low microbial richness: these individuals present adiposity-associated dyslipidemia, increased insulin resistance, and low-grade inflammation compared to their counterparts with higher genetic diversity. This deleterious phenotype is known to be associated with an increased risk of prediabetes, type 2 diabetes, liver and cardiovascular disease, and some forms of cancer. From this study, it is concluded that the concomitant improvement in the gene richness of the gut microbiome and bioclinical variables thanks to a dietary intervention suggests the possibility of moving from risk identification to risk reduction, starting from the assumption that less-rich microbiotas are also less healthy [111].

#### 4.6.1. Probiotics

A recent review by Flaig et al. reviewed a large part of the literature regarding human studies. The authors hypothesized beneficial effects of gut microbiota modulation in treating lipid metabolism disorders, in particular the relationship between gut microbiota and dyslipidemia, the impact of gut microbiota metabolites on the development of dyslipidemia, and research on dietary interventions, prebiotics, probiotics, synbiotics, and microbiota transplantation as therapeutic modalities in the prevention of cardiovascular disease. Among prebiotics used to support the growth of the synbiotic microbiota, beta-glucans have been shown to have modulating effects on the concentration of bacterial species (especially beneficial ones, such as *Bifidobacteria*, *Akkermansia*, and *Lactobacilli*), pro-immune effects, and improvement in plasma cholesterol levels. In particular, beta-glucan contained in oat fiber improves the lipid profile, reducing total and LDL cholesterol levels. This prebiotic also reduces pro-inflammatory cytokines, helping to prevent the progression of hypercholesterolemia to atherosclerosis, and increases the viscosity of fecal material, thus preventing the reabsorption of bile salts and pushing the liver to increase their production, with an increase in the uptake of circulating cholesterol. An average supplementation of 10.2 g per day of psyllium, a viscous dietary fiber, has been shown to significantly reduce the levels of LDL cholesterol, non-HDL cholesterol, and apolipoprotein B while increasing butyrogenic species such as *Roseburia*, *Lachnospira*, and *Faecalibacterium* and associated SCFAs. Finally, inulin also seems to improve the lipid profile: in combination with 2 g of phytosterols, 10 g of soy milk enriched with inulin would reduce total and LDL cholesterol. The contribution of inulin, moreover, would also depend on its ability to stimulate the growth of *Bifidobacteirum*, *Faecalibacterium*, and *Lactobacillus* and to reduce that of *Bilophila*, a bacterium that inhibits butyrate through the production of hydrogen sulfide. Among probiotics, in particular, the combination of *Lactobacilli* and *Bifidobacteria* has the ability to reduce serum levels of total cholesterol, LDL, and triglycerides. Probiotics containing *Lactobacillus, Bifidobacterium,* and *Streptococcus*, on the other hand, seem to increase HDL cholesterol levels. The combination of prebiotics and probiotics (synbiotics) has beneficial effects on the lipid profile, especially the association of xylo-oligosaccharides (XOSs) with *Bifidobacterium animalis* lactis and *Bacillus licheniformis* and that of inulin with *Lactobacillus acidophilus*, *Lactobacillus casei*, and *Bifidobacterium bifidum*. Folate is an essential molecule for the biosynthesis of nucleotides, and most intestinal bacterial species need it, as only a few species are able to produce it. Folate, therefore, by counteracting dysbiosis, has positive effects on lipids and in particular on cholesterol, decreasing LDL cholesterol and the thickness of the carotid intima media.

Finally, recently, fecal transplantation has also been proposed as a possible treatment for numerous pathologies, including metabolic pathologies with alteration of the lipid profile; by increasing *Bifidobacterium* and reducing sulfate-reducing bacteria, such as *Bilophila* and *Desulfovibrio*, this treatment seems to improve the lipid profile, especially with regard to total cholesterol and triglyceride levels [114].

#### 4.6.2. Probiotics

Some RCT studies on humans have evaluated how the association of probiotics with some molecules (including berberine and atorvastatin) can improve the dyslipidemia picture. For example, the study by Tian Y et al. aimed to evaluate the role of probiotics (in particular *Lactobacillus casei* Zhang, *Bifidobactetium animalis* subsp. *lactis* V9, and *Lactobacillus plantarum* P-8) in the treatment of hyperlipidemia in association with the administration of atorvastatin. A total of 33 patients with hyperlipidemia were recruited and randomly divided into a probiotic group (n = 18) and a control group (n = 15). The probiotic group was administered probiotics (2 g once daily) and atorvastatin 20 mg (once daily), while the control group was administered placebo (2 g once daily) and atorvastatin 20 mg (once daily). Serum and stool samples were collected for further analysis. The results showed that probiotics optimized the structure of the intestinal microbiota and decreased the amount of harmful bacteria in patients with hyperlipidemia. In fact, a significant effect was observed on the levels of total cholesterol, triglycerides, and LDL cholesterol in the probiotic and control groups (*p* < 0.05). The intestinal microbial abundance in the probiotic group was significantly higher than that in the control group after 3 months of probiotic treatment (*p* < 0.05). At the phylum level, probiotics had no notable effects on the relative abundance of *Firmicutes*, *Bacteroidetes*, and *Actinobacteria*, but increased that of *Tenericutes* and decreased that of Proteobacteria. At the genus level, probiotics increased the relative abundance of *Bifidobacterium*, *Lactobacillus*, and *Akkermansia* and decreased that of *Escherichia*, *Eggerthella*, and *Sutterella* compared with the control group at months 1, 2, and 3 (*p* < 0.05) [115].

Another study by Wang S. et al. investigated whether the combination therapy of probiotics (Prob) and berberine (BBR) associated with an antidiabetic and lipid-lowering regimen could effectively reduce postprandial lipidemia (PL) in T2D. Non-fasting lipidemia (nFL), mainly caused by postprandial lipidemia, has recently been recognized as an important risk factor for cardiovascular disease as fasting lipidemia (FL). PL is a common feature of dyslipidemia in type 2 diabetes. Blood PL (120 min after ingestion of 100 g of standard carbohydrate) was examined in 365 participants with T2D from the Probiotics and BBR on the Efficacy and Change of Gut Microbiota in Patients with Newly Diagnosed Type 2 Diabetes (PREMOTE study), a placebo-controlled RCT. Participants were randomly assigned to one of four groups in a 1:1:1:1 ratio as follows: BBR (0.6 g per 6 pills, twice daily before a meal) + probiotics (4 g per 2 powder strips, once daily at bedtime) (Prob + BBR), probiotics + placebo (Prob), BBR + placebo (BBR), or placebo + placebo (Plac). Treatments were administered for 12 weeks. The probiotic products contained nine proprietary strains of probiotics (*Bifidobacterium longum*, *Bifidobacterium breve*, Lactococcus gasseri, *Lactobacillus rhamnosus*, *Lactobacillus salivarius*, *Lactobacillus crispatus*, *Lactobacillus plantarum*, *Lactobacillus fermentum*, and *Lactobacillus casei*), and each sachet contained ≥50 billion CFU of freeze-dried live bacteria. Prob + BBR was superior to BBR or Prob alone in improving postprandial total cholesterol and low-density lipoprotein cholesterol levels, with a decrease in multiple postprandial lipidomic metabolite species after 3 months of follow-up. This effect was linked to changes in fecal *Bifidobacterium breve* levels in response to treatment with BBR alone or Prob + BBR. Four fadD genes encoding long-chain acyl-CoA synthetase have been identified in the genome of this *B. breve* strain and were transcriptionally activated by BBR. In vitro treatment with BBR further reduced the concentration of FFA in the culture medium of *B. breve*. Therefore, fadD activation by BBR might enhance the import and mobilization of FFA into *B. breve* and dilute intraluminal lipids for absorption, mediating the effect of Prob + BBR on PL. This study confirmed that BBR and Prob can exert a synergistic lipid-lowering effect on PL, acting as a reservoir of intestinal lipids to achieve better control of lipidemia and cardiovascular risk in T2D [116].

A further randomized, double-blind, placebo-controlled study by Trotter RE et al. evaluated whether supplementation with Bacillus subtilis DE 111 could be useful to improve dyslipidemia and consequently improve risk factors associated with cardiovascular disease. This 4-week study was conducted on 94 men and women aged 18 to 65 years with a body mass index of 20 to 34.9. Supplementation with *B. subtilis* 15 mg daily resulted in a significant reduction in total cholesterol compared to baseline measures, as well as in non-high-density lipoprotein cholesterol. In addition, modest improvements in endothelial function and significant changes in several plasma lipids were observed [117].

Several meta-analyses have shown that probiotic supplementation can effectively improve lipid profiles in subjects with dyslipidemia.

A meta-analysis by Ettinger G et al. aimed to examine the role of the microbiome in the prevention and treatment of cardiovascular disease (CVD). One of the most studied applications of probiotic therapy for CVD is the reduction in serum cholesterol. Accumulation of LDL-C in the blood is a precursor to hypertension and hyperlipidemia and causes the formation and accumulation of atherosclerotic plaques in the arteries. Meta-analyses of randomized controlled clinical trials were performed to evaluate the effect of probiotic consumption on serum LDL-C and total cholesterol levels. Pooled data from a total of 485 participants with “high”, “borderline high”, and “normal” serum cholesterol levels found that probiotic consumption significantly reduced LDL-C and total cholesterol levels in all categories, compared to control. The metabolism of cholesterol, a precursor of bile acids, is mediated by gut microbes that express the enzyme bile salt hydrolase (BSH). Probiotics with high BSH activity (*Lactobacillus* and *Bifidobacterium*) promote the deconjugation of bile acids in the intestine into secondary amino acid conjugates. When these secondary conjugates are excreted, cholesterol is broken down to replace the converted bile salts. Overall, this process promotes cholesterol catabolism, resulting in lower serum levels. Several specific probiotic strains have been identified as effective for the management of hypercholesterolemia. The most effective probiotic strain, clinically proven to reduce LDL-C levels by approximately 11.6% in hypercholesterolemic adults, is *Lactobacillus reuteri* NCIMB 30242 [122].

A meta-analysis of interventional studies conducted by Shimizu M et al. demonstrated that probiotic supplementation could be useful in the primary prevention of hypercholesterolemia, leading to a reduction in cardiovascular disease risk factors. Thirty-three randomized controlled trials comparing probiotic supplementation with placebo or a control group (i.e., no treatment) were included. Eleven studies describing data on pre- and post-intervention differences in serum lipids, including total cholesterol, LDL, HDL, and TG, were then selected. These eleven studies consisted of eight using fermented milk products and four using probiotic capsules as test drugs, including one that included both a probiotic and a prebiotic, i.e., a fructo-oligosaccharide in a rice starch base. These interventions produced changes in total cholesterol (mean difference −0.17 mmol/L) and low-density lipoprotein cholesterol (mean difference −0.22 mmol/L). High-density lipoprotein cholesterol and triglyceride levels did not differ significantly between the probiotic and control groups. Reductions in total cholesterol and LDL levels with the probiotic intervention were greater in mildly hypercholesterolemic subjects than in normocholesterolemic subjects, such that the reduction in LDL levels after the probiotic intervention in hypercholesterolemic patients would lead to an approximately 8% reduction in major cardiovascular events. A subanalysis evaluated the long-term probiotic intervention (>4 weeks) as statistically more effective in reducing TC and LDL-C than the short-term intervention (≤4 weeks), and high-dose probiotics reduced LDL-C levels more effectively than low-dose probiotics. Furthermore, reductions in TC and LDL-C in older adults were greater than those in younger individuals, likely due to higher baseline values in older adults. In detail, it was then seen that *Lactobacillus acidophilus* and Gaio, a yogurt product fermented with Causido composed of one strain of *Enterococcus faecium* and two strains of *Streptococcus thermophilus*, reduced TC and LDL levels to a greater extent than the other bacterial strains [123].

A review by Sivamaruthi et al. analyzed the ability of probiotics to reduce cholesterol levels in hypercholesterolemic subjects (total COL, LDL, and triglyceride reduction, and improvement in HDL values). The effect of probiotics (*Enterococcus faecium* CRL 183 and *Lactobacillus helveticus* 416) fermented with soy products containing isoflavones on the lipid profile of moderately hypercholesterolemic male subjects was studied. Supplementation of the soy product (equivalent to 10 CFU of probiotic strain) with 50 mg of isoflavone per day for 42 days significantly improved total cholesterol (TC) and LDL cholesterol in the subjects, while HDL level remained unchanged. Furthermore, a 12-week supplementation with a mixture of *L. plantarum* strains (10 CFU/day) significantly increased HDL levels and reduced LDL levels, total cholesterol, triglycerides, the LDL/HDL ratio, and LDL levels in hypercholesterolemic subjects.

In dyslipidemic children, the effect of supplementation with a mixture of *Bifidobacterium* strains (*B. animalis* lactis MB 2409, *B. longum* BL04, and *B. bifidum* MB 109B) for three months was analyzed: the concentration of serum levels of total cholesterol, HDL, TG, and LDL was significantly improved after three months of probiotic supplementation compared to baseline values.

Consumption of a single probiotic strain (*E. faecium* M-74; 2 × 10^9^ CFU/day) and selenium (50 μg) for one year did not alter the level of HDL-C and TG while significantly reducing TC and LDL-C in elderly subjects compared to baseline and placebo.

Intervention of probiotic capsules containing *L. acidophilus* and *B. bifidum* (109 CFU/capsule; three capsules/day) for six weeks significantly reduced the level of TC and LDL-C in hypercholesterolemic patients. There were no changes in the levels of TG and blood sugar of the patients, and the level of HDL-C was significantly reduced during the study period.

Supplementation of 30 g of probiotic cheese containing *L. acidophilus* LA5 and *B. lactis* BB12 (each 5 × 10^6^ CFU) with or without 30 g of chicory root extract significantly reduced the content of TC, triglycerides, and LDL-C and increased that of HDL-C in healthy volunteers in seven weeks of the experimental period. Consumption of probiotic yogurt (300 g daily) containing *L. acidophilus* La5 (~4.14 × 10^6^ CFU/g) and *B. lactis* Bb12 (~3.61 × 10^6^ CFU/g) for six weeks significantly improved the lipid profile of patients with type 2 diabetes mellitus. In particular, the levels of TC and LDL-C and the ratios of LDL:HDL-C and TC:HDL were significantly reduced compared to baseline and placebo control [119].

A further systematic review by Sivamaruthi analyzed dietary interventions with probiotics in humans and their effects on cardiovascular risk factors and hypercholesterolemia. Seventeen studies were considered. *Lactobacillus* (in nine studies), *Bifidobacterium* (in eight studies), *and Enterococcus* (in two studies) were the most studied genera in these reports. Both single and synbiotic strains were analyzed. One study tested *Bifidobacterium longum* BB536 and red yeast rice in 33 patients with low CVD risk and no CVD risk and found a reduction in LDL-c levels. In another study, *L. plantarum* ECGC 13110402 was administered, together with the regular diet, to 23 people with normal to mild hypercholesterolemia. *L. plantarum* ECGC 13110402 has a high bile salt hydrolase activity, with evidence of a reduction in the level of LDL-c and consequently of the risk of CVD. The soy product supplemented with isoflavones and fermented with *Enterococcus faecium* CRL 183 and *L. helveticus* 416 was administered to 17 patients; at the end of 42 days, it was observed that the probiotic intervention significantly reduced the level of LDL-c up to 14.8%.

The probiotic mixture composed of *B. lactis* MB 2409, *B. bifidum* MB 109B, and *B. longum* BL04 showed a significant reduction in TC, LDL-C, and TG levels and an increase in HDL-c levels in dyslipidemic children. A probiotic mixture supplementation containing three strains of *L. plantarum* significantly reduced TC (13.6%), LDL-C, and oxidized LDL-C levels in hypercholesterolemic adults. Finally, the supplementation of a synbiotic mixture containing *L. acidophilus*, *B. bifidum*, and oligofructose effectively increased HDL-C and reduced fasting blood glucose in elderly people with type 2 diabetes mellitus [120].

Finally, a systematic review by Gadelha et al. examined the effects of probiotic supplementation on the prevention and treatment of dyslipidemia. Considering that the role of the microbiota in the development of hypercholesterolemia has already been studied in the past, since people with hypercholesterolemia have a lower bacterial diversity and a different profile of microorganisms, this study has shown that probiotic supplementation should be indicated as an additional treatment for lipid profile alterations. This review included 14 clinical studies involving subjects over 18 years of age, mostly with dyslipidemia. The genera most often administered to the groups treated with probiotics were *Lactobacillus* and *Bifidobacterium*. Other genera used were *Saccharomyces, Streptococcus, and Enterococcus,* to a lesser extent. Probiotic supplementation significantly reduced total cholesterol, LDL, and triglycerides and increased HDL values, especially when associated with other treatments (statins). The group with total cholesterol > 200 mg/dL had the best response to probiotic treatment. Some benefits have also been observed in anthropometric variables (waist circumference), glycemic control, oxidative stress, inflammatory markers, and the immune system [121].

#### 4.6.3. Symbiotic

The double-blind randomized controlled trial by Salamat S. et al. investigated the effects of multispecies synbiotic supplementation on serum interleukin 10 and fecal short-chain fatty acids (SCFAs) in patients with dyslipidemia. Fifty-six adult men aged 60 years or younger with dyslipidemia with TG of 200–400 mg/dL and LDL of 130–160 mg/dL were recruited and randomly assigned to intervention and control groups. Subjects received synbiotic powder or placebo twice daily for 12 weeks. Each sachet of synbiotic supplement contained 3 × 10^10^ CFU of six freeze-dried probiotic strains, including *Lactobacillus* (L.) *acidophilus* ATCC4356, *L. fermentum* DSM14241, *L. plantarum* ATCC14917, *Bifidobacterium* (B.) *longum* BAA-999, *B. lactis* ATCC27536, and *Saccharomyces (S.) boulardii* CNCM I-745, and 5 g inulin and FOS as prebiotics. Blood and stool samples were collected at baseline and at the end of the study. Food intake, physical activity, anthropometric measures, serum IL-10, and fecal SCFAs were assessed before and after the intervention. Synbiotic supplementation increased fecal SCFAs and improved inflammation in adult men with dyslipidemia. Indeed, serum IL-10 increased in the synbiotic group, and, in addition, synbiotic supplementation increased fecal concentrations of acetate, butyrate, propionate, and valerate. In evaluating the effects of synbiotic consumption on serum lipid profile, after 12 weeks of supplementation, a significant increase in fecal abundance of *Lactobacillus* and *Bifidobacterium* and serum HDL was observed in the synbiotic group. These results suggest that this synbiotic mixture can be considered a promising adjunctive therapy to improve GM metabolites and metabolic status [118].

In conclusion, alterations in the gut microbiota induced by a high-fat diet can negatively affect the composition of the microbiota, reducing beneficial bacteria such as *Bifidobacterium* and *Lactobacillus* and favoring the proliferation of pathogenic genera such as *Bilophila wadsworthia* and Enterobacteria. These alterations are significantly related to the development of dyslipidemia and contribute to a vicious cycle that aggravates metabolic and cardiovascular conditions. Several studies have explored the potential of probiotics in the treatment of dyslipidemia, with significant improvements in LDL cholesterol, HDL cholesterol, and triglyceride levels, in addition to the reduction in systemic inflammation. Recent meta-analyses [122,123] have confirmed that probiotic supplementation, particularly the *Lactobacillus* and *Bifidobacterium* genera, reduces total and LDL cholesterol levels, with more marked effects in hypercholesterolemic subjects than in normocholesterolemic subjects. Among the most effective probiotics, *Lactobacillus reuteri* NCIMB 30242 has been shown to reduce LDL-C levels by approximately 11.6% in hypercholesterolemic adults [122].

To date, 33 randomized clinical trials (RCTs) have evaluated the efficacy of various probiotic strains. Among these, 11 studies have observed significant improvements in lipid profiles with the use of single and combined strains of *Lactobacillus and Bifidobacterium* [123]. Furthermore, more recent studies have investigated the efficacy of synbiotics containing prebiotics such as inulin (5–10 g/day) and fructo-oligosaccharides (5 g/day) in combination with probiotics, demonstrating an improvement in cholesterol levels and an increase in the abundance of beneficial bacteria such as *Lactobacillus* and *Bifidobacterium* [118,119]. Regarding factors influencing efficacy, long-term (>4-week) probiotic intervention was statistically more effective in decreasing TC and LDL-C than short-term (≤4-week) intervention, and high-dose probiotics more effectively reduced LDL-C levels than low-dose probiotics. Moreover, reductions in TC and LDL-C in elderly subjects were greater than those in younger individuals, likely due to higher baseline values in the elderly population [123].

Several specific probiotic strains have been identified as effective for managing hypercholesterolemia. Research indicates that certain strains can significantly lower total cholesterol (TC) and low-density lipoprotein cholesterol (LDL-C) levels in hypercholesterolemic patients. One effective probiotic strains is *Lactobacillus reuteri* NCIMB 30242 (clinically proven to reduce LDL-C levels by approximately 11.6% in hypercholesterolemic adults) [122]. 

*Lactobacillus acidophilus* was shown to effectively lower serum cholesterol levels due to its ability to assimilate cholesterol in vitro [119]; *Lactobacillus plantarum* was associated with reductions in TC and LDL-C in clinical studies [119]; *Bifidobacterium lactis* demonstrated significant cholesterol-lowering effects, particularly in combination with other probiotic strains; *Lactococcus lactis* exhibits hypocholesterolemic effects and can be part of probiotic mixtures aimed at reducing cholesterol levels [119].

Probiotic strains such as *Lactobacillus reuteri*, *Lactobacillus acidophilus*, and *Bifidobacterium lactis* are particularly effective in lowering cholesterol levels in hypercholesterolemic patients. When selecting probiotic products, is it important consider those that contain these specific strains for optimal results in managing dyslipidemia.

Finally, probiotics have been shown to impact cholesterol levels differently in elderly individuals compared to younger adults, primarily due to variations in baseline cholesterol levels and the physiological changes associated with aging. Probiotic interventions tend to produce more significant reductions in total cholesterol (TC) and low-density lipoprotein cholesterol (LDL-C) in elderly individuals than in younger adults. This is attributed to the generally higher baseline cholesterol levels found in older adults, making them more responsive to probiotic supplementation [123].

In conclusion, probiotics appear to be more effective in reducing cholesterol levels in elderly individuals compared to younger adults, primarily due to higher baseline cholesterol levels and age-related physiological changes. However, further research is necessary to establish clear guidelines for their use in this demographic.

Despite the promising results, there is a call for more robust clinical trials specifically targeting the elderly population to better understand the optimal strains, dosages, and long-term effects of probiotics on cholesterol management.

### 4.7. Sarcopenia

According to EWGSOP2, sarcopenia is characterized by low muscle mass, measured by DEXA, and low muscle strength, measured by handgrip. In addition to being a condition related to old age, sarcopenia is common in several chronic diseases, including tumors, dementia, chronic renal failure, liver cirrhosis, and heart failure [237].

The prevalence of sarcopenia is estimated to be around 10–16% worldwide, ranging from 18% in subjects affected by diabetes up to 66% in patients with esophageal cancer [238].

In geriatric hospital departments, the prevalence is around 35% [239].

The scientific community has shown a growing interest in defining the relationship between the intestinal microbiota and sarcopenia, hypothesizing the presence of a real “gut–muscle” axis. This concept takes on even more importance considering how elderly individuals experience alterations in both their muscles and their intestinal microbiota. A healthy gut microbiota may contribute to maintaining skeletal muscle mass and function by promoting protein synthesis, mitochondrial function, and the balance between anti- and pro-inflammatory factors [240].

The development of sarcopenia in the elderly is the result of several pathophysiological processes related to malnutrition, physical inactivity, immunosenescence, inflammaging, mitochondrial alterations, oxidative stress, and anabolic resistance. In particular, immunological and inflammatory mechanisms are strongly influenced by the intestinal microbiota. A prospective study by the group of Kang et al. used sequencing techniques on fecal samples from 27 individuals affected by sarcopenia (identified by BIA, handgrip, and chair test) and 60 healthy controls. The alpha-diversity of the microbiota was found to be strongly decreased in sarcopenic individuals compared to healthy controls (*p* < 0.05), suggesting that the aging process could be a potential cause of “disturbance” of the gut–muscle axis. This study also found a reduction in beta-diversity (*p* = 0.0001) and in particular in butyrate producers, which are essential for the production of SCFAs capable of modulating inflammation. Firmicutes were found to be significantly decreased (40.4% in the sarcopenic group vs. 54.4% in controls, *p* = 0.005). The Porphyromonadaceae family was instead found to be increased in the microbiota of subjects affected by sarcopenia (*p* < 0.05), placing this element as a possible taxonomic biomarker of sarcopenia or possible sarcopenia [130]. The main representative of the Porphyromonadaceae family is *Porphyromonas gingivalis*, a commensal bacterial species of the oral cavity related to the onset of atherosclerosis, Alzheimer’s disease, rheumatoid arthritis, and diabetes [241].

An observational, cross-sectional study on a total of 1417 individuals aged over 50 years from different rural Chinese villages, using a shotgun sequencing technique, demonstrated that in subjects with sarcopenia (identified by short physical performance battery (SPPB) and handgrip), there is an alteration in the function and composition of the intestinal microbiota. The 141 subjects identified as sarcopenic and the 1276 controls were compared with each other. Although no significant change in alpha-diversity of the microbiota was found, beta-diversity differed significantly between the two groups (*p* = 0.02). Sarcopenic subjects also had higher abundances of at least six bacterial species, especially Clostridia (*p* = 0.002) [129].

An Italian study by the Ticinesi group, conducted on 5 elderly sarcopenic subjects and 12 healthy controls, using shotgun sequencing techniques, detected a substantial reduction in butyrate-producing species, *Faecalibacterium prausnitzii* (*p* = 0.019), *Roseburia inulinivorans* (*p* = 0.006), and *Alistipes shahii* (*p* = 0.019). From a genetic point of view, a deficiency in several genes involved in the synthesis of short-chain fatty acids, carotenoids, and biotransformations of isoflavones and amino acids was also found [128].

In an observational study conducted by Maslennikov, 46 patients with liver cirrhosis and sarcopenia were enrolled; an increase in potential pathogens such as Eggerthella was found (*p* = 0.01), which would act negatively by creating a pro-inflammatory environment. An excess of these bacteria in the microbiota would in fact promote bacterial translocation into tissues and an increase in ammonia levels, which in turn increases protein catabolism and levels of myostatin, a protein that inhibits muscle growth [242].

Another observational study that found similar results was conducted at the Gemelli University Institute in Rome by the group of Ponziani et al. This study evaluated, 50 patients with cirrhosis and 50 controls with an average age of 69 years by collecting fecal samples. Among the cirrhotic patients, 19 were found to be affected by sarcopenia. In the microbiota of patients with cirrhosis and sarcopenia, a lower abundance of Akkermansia and Prevotella was found (*p* = 0.4), with a greater presence of Eggerthella (*p* = 0.001) [243].

A 2023 study by Liu et al. found, in a group of 141 sarcopenic subjects (identified by handgrip, bioimpedance, and walking test) aged 50 years or older, a deficiency in *Prevotella copri* (*p* = 0.01) and branched chain amino acid metabolism when compared with 142 healthy controls. Sarcopenic subjects also showed an abundance of Bifidobacteria, producers of SCFAs (*p* = 0.04) [127].

Prevotella (*p* = 0.02) and *Prevotella copri* (*p* = 0.017) deficiency in sarcopenic subjects has been reported in other previous studies, including one conducted in Korea on 60 individuals aged over 60 years, including 27 with sarcopenia and 33 healthy controls [244]. In this study, subjects with sarcopenia also had an abundance of the genus Anaerotruncus (*p* = 0.005).

*Prevotella copri* is the major species of the genus Prevotella and is usually abundant in the human microbiota, especially in diets rich in fiber and plant foods. The abundance of this species in the microbiota has been associated with both positive and negative factors. An abundance of *P. copri* has been associated with the onset of rheumatoid arthritis, diabetes mellitus type 1 and 2, myasthenia gravis, and some types of cancer; at the same time, a deficiency in this species has been associated with dermatological disorders, Parkinson’s disease, sarcopenia, and autism spectrum disorder. These controversial results probably derive from the presence of different strains of *P. copri* [245].

An Italian study by Ponziani’s group in 2020 also found, by sequencing the genomic material obtained from fecal samples via Illumina MiSeqTM, an increased presence of Bifidobacteriaceae (*p* = 0.013) in sarcopenic subjects (n = 18) aged over 70 years compared to healthy controls (n = 17). In this study, subjects affected by sarcopenia also presented an abundance of Peptostreptococcaceae (*p* = 0.008) [246]. Peptostreptococcaceae are anaerobic Gram-positive bacteria commensal of the vaginal and gastrointestinal flora, responsible, however, for endocarditis, and, according to more recent studies, it may be related to the development of colorectal cancer [247].

A Japanese study by Wang et al. hypothesized that *Bifidobacterium longum* deficiency may be an additional predictive factor for the development of sarcopenia. This study enrolled 50 sarcopenic subjects and 50 healthy controls and performed an assessment of the microbiota composition starting from fecal samples. The material was sequenced using the Illumina Novaseg 6000 instrument. From the statistical analysis performed, *Bifidobacterium longum* was able to predict the presence of sarcopenia with a sensitivity of 53% and a specificity of 74%. *Prevotella coprii* was found to have a sensitivity of 4.1% and a specificity of 98% [125].

In conclusion, several studies have evaluated how, in sarcopenic patients, there are various alterations in the intestinal microbiota, reinforcing the idea that there is a “gut–muscle” axis capable of positively or negatively influencing the musculoskeletal system. Patients with sarcopenia would especially be deficient in bacteria producing short-chain fatty acids, while in their microbiota there is a greater abundance of potentially pathogenic bacteria that could therefore contribute to building a pro-inflammatory picture, favoring bacterial translocation through the intestinal walls.

#### Probiotics

Acting by modifying the microbiota seems to show great potential in the treatment of sarcopenia.

Several studies on animal models affected by catabolic conditions have investigated the use of probiotic strains (mainly Lactobacilli) in the treatment of sarcopenia, with positive effects on muscle mass, strength, endurance, and reductions in mortality. This effect is probably linked to the anti-inflammatory effect of the supplementation, which counteracts dysbiosis and conditions such as leaky gut and upregulation of the NF-kb pathway. The targets at the muscular level are numerous and probably different between the different probiotic strains: among these there are the stimulation of anabolic factors such as Insulin Growth Factor-1, the mTOR pathway, the regulation of the energy state of myocells via AMPK, inhibition of proteolysis, and inhibition of the oxidative state [118,237,238].

The use of probiotics may be effective in improving body composition. This has been demonstrated, for example, in healthy elderly subjects in the double-blind randomized controlled trial of Chaiyasut’s group in 2022. By administering a supplement containing probiotics (2.0 × 10^10^ CFU of *L. paracasei* HII01; 2.0 × 10^10^ CFU of *B. breve*; 1.0 × 10^10^ CFU of *B. longum*) for 12 weeks to 24 non-sarcopenic subjects aged over 55 years, an improvement in several anthropometric values was obtained compared to controls (n = 24), including a decrease in % visceral and subcutaneous fat and an increase in % muscle mass, measured with a bioimpedance scale [239].

Focusing on subjects affected by sarcopenia, a recent randomized double-blind study by the group of Lee et al. hypothesized that a supplementation with *Lactobacillus plantarum* TWK10 could improve muscle mass and function in elderly people considered frail. In this study, a sample of 55 subjects aged between 55 and 85 years were randomized into three groups: placebo, low-dose probiotic treatment (2 × 10^10^ CFU per day), and high-dose probiotics (6 × 10^10^ CFU per day). For 18 weeks, the subjects took two capsules per day of probiotic or placebo. After 18 weeks, the group that had taken high-dose probiotics had obtained a clear improvement in handgrip strength (*p* = 0.0187) and chair test (*p* = 0.002). The group that had taken high-dose supplementation also showed an increase in lean mass measured with DXA (*p* = 0.002), unlike the placebo group where an increase in fat mass was found [126].

A 2022 randomized, double-blind controlled trial conducted by the group of Karim et al. lasting 12 weeks evaluated how a probiotic supplementation could influence secondary sarcopenia due to heart failure in male patients aged 58–73 years with heart failure. Forty-four patients were then administered a probiotic containing 112 billion colony-forming units (CFU) of *B. longum* DSM 24736, *B. breve* DSM 24732, and DSM 24737; lactobacilli (DSM 24735, DSM 24730, DSM 24733, *L. delbrueckii* subsp. bulgaricus DSM 24734); and *Streptococcus thermophilus* (DSM 24731), while 48 subjects took the placebo. The group that received the probiotic obtained an improvement in walking speed and handgrip (*p* < 0.05). Potential biomarkers for the diagnosis of sarcopenia concerning the Wnt pathway and in particular the presence of Dkk-3 and SREBP1 at the plasma level were also analyzed [133].

The same group in 2022 published another randomized double-blind study, conducted on 104 male patients aged 63–73 years, affected by sarcopenia secondary to Chronic Obstructive Pulmonary Disease (COPD), using the same type of probiotic; 53 patients took placebo, while 47 took the treatment, for a total duration of 16 weeks. The group that took probiotics obtained an improvement in handgrip and walking speed and a functional improvement assessed with the SPPB (*p* < 0.05) [134].

An additional controlled trial in 2022 confirmed the potential of using probiotics in the treatment of sarcopenia. In this study, the subjects were randomized to the placebo group (n = 28) or to the group (n = 22) that received a mixture containing 500 mg of omega-3, 2.5 g of leucine, and 30 billion colony-forming units of *Lactobacillus paracasei* PS23. After two months of treatment, the group receiving the probiotic achieved a significant increase in plasma amino acid concentration (*p* = 0.001) and appendicular lean mass (ALM) (*p* < 0.05) as well as a reduction in visceral fat mass (*p* = 0.001), measured by DXA [135].

A study by the group of Ford et al. administered a probiotic (composition: *Bifidobacterium bifidum* HA-132 (1.54 billion), *Bifidobacterium breve* HA-129 (4.62 billion), *Bifidobacterium longum* HA-135 (4.62 billion), *Lactobacillus acidophilus* HA-122 (4.62 billion), and *Lactobacillus plantarum* HA-119 (4.62 billion)), associated with a high-protein diet (1.5 g per kg of body weight) in 26 female subjects with an average age of 73 years. In these subjects, a significant increase in lean mass (*p* = 0.03) assessed by bioimpedance analysis was found [136].

A recent study published in 2024 by Qaisar’s group randomized 134 male subjects (age > 65 years) affected by sarcopenia into a placebo group (n = 63) and a group administered a probiotic (n = 60) for 16 weeks. The probiotic, with a total of 112 billion colony-forming units, contained bifidobacteria (*B. longum* DSM 24736, *B. breve* DSM 24732, DSM 24737), *Streptococcus thermophilus* DSM 24731, and lactobacilli (DSM 24735, DSM 24730, DSM 24733, *L. delbrueckii* subsp. bulgaricus DSM 24734). The different aspects related to frailty and sarcopenia were evaluated through different parameters, defined by the acronym SarQoL, which includes different scores (D1: physical and mental health; D2: locomotion; D3: body composition; D4: functionality; D5: activity of daily living; D6: leisure activities; D7: concern for one’s condition). At the end of the 16 weeks, the group that received the probiotic showed an improvement in hand grip strength in percentage and gait speed (*p* < 0.05) and in several SarQoL scores (D1; D2; D4; D5; D6), as well as a reduction in fecal zonulin values (*p* < 0.05) [137].

Finally, a study by Tominaga et al. evaluated the use of a prebiotic, 1-kestose, which can promote the proliferation of Bifidobacteria, in the treatment of sarcopenia patients. After 8 weeks of administration of 20 g per day of 1-kestose, a reduction in the percentage of fat mass measured by bioimpedance and an improvement in parameters regarding strength (measured with handgrip) and muscle mass were observed, as well as an increase in the population of *Bifidobacterium longum* in the microbiota of patients sequenced with Illumina Miseq software (*p* < 0.01) [132].

In conclusion, a condition of dysbiosis, or in general, of alteration of the intestinal microbiota due to age or external factors, could be implicated in the development of sarcopenia through different mechanisms, mainly linked to the release of pro-inflammatory factors. Several studies have already demonstrated the potential of using probiotics in the treatment of this condition, detecting significant improvements in maintaining both lean mass and muscle strength. These findings reinforce the hypothesis that a condition of dysbiosis can lead to a generalized inflammatory picture with negative consequences on different organs and systems, including the musculoskeletal one.

RCT studies in humans are still few (currently six studies) but have evaluated numerous strains: two studies evaluated a single strain of *Lactobacillus plantarum* TWK10 (2 × 10^10^ CFU per day), and *Lactobacillus paracasei* PS23 (30 billion colony-forming units); three studies considered an association consisting of 112 billion colony-forming units of *B. longum* DSM 24736, *B. breve* DSM 24732, and DSM 24737, *Streptococcus thermophilus* DSM 24731, and lactobacilli (DSM 24735, DSM 24730, DSM 24733, *L. delbrueckii* subsp. bulgaricus DSM 24734). All studies have shown that the intake of probiotics has proven effective in improving muscle mass and strength.

Table 7 summarizes the evidence on studies investigating the composition of the microbiota in sarcopenic patients, highlighting reductions in microbial diversity, deficiencies in key species such as *Bifidobacterium longum* andK *Prevotella copri*, and increases in pro-inflammatory bacteria. Table 3 focuses on studies evaluating the effects of probiotic or prebiotic interventions on improving muscle strength and body mass in subjects with sarcopenia.

### 4.8. Non-Alcoholic Fatty Liver Disease (NAFLD)

Non-alcoholic fatty liver disease (NAFLD) comprises a spectrum of liver diseases ranging from steatosis to non-alcoholic steatohepatitis (NASH) to cirrhosis in the absence of excessive alcohol consumption (typically less than 20 g per day for women and 30 g for men). “Simple” steatosis, i.e., without the presence of inflammation or necroinflammation-related disease, has not been associated with liver disease-related morbidity but represents the antechamber of NASH, and it is known that the latter can lead to progressive liver fibrosis, cirrhosis, and hepatocellular carcinoma, in addition to increasing cardiovascular risk. NAFLD is strongly associated with obesity, insulin resistance, type 2 diabetes mellitus, and dyslipidemia and can be considered the hepatic manifestation of metabolic syndrome [248].

Globally, NAFLD is a leading cause of liver disease, with an estimated incidence of 47 cases per 1000 population, and is higher in males than females. The estimated global prevalence of NAFLD among adults is 32%, and again is higher in males (40%) than females (26%). The global prevalence of NAFLD has increased over time, from 26% in studies from 2005 or earlier to 38% in studies from 2016 onward. The prevalence of NAFLD varies substantially by region of the world, due to different rates of obesity and genetic and socioeconomic factors, with values exceeding 40% in the Americas and Southeast Asia. Prevalence is expected to increase significantly in several regions of the world by 2030 if current trends are not changed [249].

The hypothesis underlying the development of NAFLD involves multiple parallel factors that simultaneously generate and maintain inflammation, promoting liver damage and fibrosis accumulation. The main protagonists in this scenario seem to be a bad lifestyle (high-fat diet, HFD), lipotoxicity, intestinal barrier dysfunction, and dysbiosis [147].

Since lipid and carbohydrate metabolism involves different types of bacteria and is associated with obesity-related energy metabolism, energy metabolism-related disorders, including hyperlipidemia, atherosclerosis, diabetes, and inflammation, could be regulated by the gut microbiota and its metabolites [140,250,251].

In recent years, there has been a rapid increase in scientific articles exploring the contribution of the gut microbiota in the physiopathogenesis of NAFLD, with a growing body of evidence in this regard [148,149,150].

Studies on animal models, particularly germ-free animals and microbiota transplantation, have demonstrated a potential causal role of gut microbiota alterations in NAFLD [252].

The first evidence on the role of the intestinal microbiota in host adiposity comes from studies on germ-free (GF) animals, that is, animals devoid of bacteria and raised in sterile isolators. In 1983, Wostmann and colleagues observed that GF rodents require 30% more calories to maintain body mass than conventional rodents (which have their own microbiota) [253]. The potential mechanisms explaining this observation remained unclear until recent studies, which are based on experiments conducted mainly in Jeffrey Gordon’s laboratory at Washington University in Saint Louis (MO, USA) that pioneered the study of the gut microbiota as a factor influencing adipose tissue accumulation and obesity. They first found that conventionally raised mice had 42% more total body fat and 47% more gonadal fat than GF mice, although GF mice consumed more food [220]. They subsequently demonstrated that colonization of GF mice with cecal-derived microbiota from conventional mice produced a 60% increase in body fat mass within 2 weeks. The increase in body fat was accompanied by insulin resistance, adipocyte hypertrophy, and increased circulating levels of leptin and glucose [220]. This was partly explained by the ability of the gut microbiota to degrade non-digestible polysaccharides into monosaccharides that could be absorbed, leading to increased hepatic lipogenesis in the host. Furthermore, inoculation of the gut microbiota suppresses the intestinal expression of angiopoietin 4 (ANGPTL4), a circulating inhibitor of lipoprotein lipase (LPL). Conventionalization thus leads to increased adipocyte LPL activity and thus increased cellular fatty acid uptake and triglyceride accumulation in adipocytes [220]. The physiological importance of ANGPTL4 was further established by the demonstration that GF ANGPTL4^−/−^ mice have the same degree of adiposity as their conventional counterparts. Finally, unlike conventional mice, GF mice fed a high-fat, high-sugar diet are protected from diet-induced obesity (DIO) [254]. This particular phenotype was associated with increased levels of AMP-activated protein kinase (AMPK) in skeletal muscle and liver [254]. AMPK is an enzyme and functions as a fuel indicator that monitors cellular energy status and stimulates fatty acid oxidation in peripheral tissue. Therefore, the gut microbiota can suppress fatty acid oxidation in skeletal muscle through a metabolic pathway involving AMPK phosphorylation. It was also observed that GF mice receiving a high-fat diet showed increased insulin sensitivity with improved glucose tolerance and reduced insulinemia compared to conventional mice. This was associated with reduced hypercholesterolemia, moderate hepatic cholesterol accumulation, and increased fecal cholesterol excretion, suggesting altered cholesterol metabolism in GF mice [255]. However, this resistance to diet-induced obesity may depend on the genetic background of the mice and the composition of the diet [256].

The obesity resistance phenotype in GF mice was later found to be strongly dependent on the sugar composition of the diet and not so much on the amount of lipids [254].

Furthermore, similar body weights and adiposity were observed in conventional GF and Fischer 344 rats [257]. In these rats, GF status was associated with increased intestinal ANGPTL4 and reduced hepatic lipogenesis, as well as increased adipocyte size, suggesting that the impact of the gut microbiota on fat accumulation may be more complex than proposed by early pioneering work.

Subsequently, Turnbaugh et al. demonstrated that this characteristic is transferable through fecal microbiota transplantation (FMT), in line with what was previously indicated, namely that the “obese microbiome” helps to accumulate more energy from the diet [258].

Furthermore, Samuel et al. observed that the short-chain fatty acid (SCFA)-binding G protein-coupled receptor Gpr41 can modulate the effects of the gut microbiota on host adiposity by comparing wild-type Gpr41-deficient and GF mice with and without a fermentative microbial community model [259].

The comparison between GF and conventional (CV) mice was also used for the correlation between microbiota and liver diseases. A difference between GF and CV mice was found in the expression of several important liver genes, including CAR (the constitutive androstane receptor), a member of the nuclear hormone receptor family encoded by the NR1I3 gene [260]. CAR, together with other receptors, functions as a sensor of endobiotic and xenobiotic substances. The absence of intestinal microbiota in GF mice results in elevated amounts and accumulation of CAR ligands, including bilirubin, bile acids, and steroid hormones, leading to an alteration of xenobiotic metabolism in the liver, which could favor the development of NAFLD [261].

Cohousing experiments with mice prone to developing NASH due to genetic modifications in the inflammasome pathway and healthy wild-type mice demonstrate that sharing microbiota through coprophagy leads to the development of hepatic steatosis and inflammation in wild-type mice [262]. Furthermore, a direct FMT (from weight-matched obese mice with or without steatosis to germ-free recipients) replicates some alterations in NAFLD. These liver alterations include increased hepatic triglyceride content and increased expression of hepatic genes involved in lipid uptake, lipogenesis, fatty acid catabolism, and VLDL export [263].

Although animal models may seem like a good solution to explore the connection between the microbiota and liver disease, they have many limitations, especially related to the extrapolation of information to humans [252]. In fact, mouse models do not develop the full histological spectrum of lesions observed in human NAFLD, and in those where this is possible, such as choline-deficient mice, the pathophysiology is completely different between mice and humans [264].

The immune system seems crucial in the microbiota–NAFLD relationship. Interestingly, in fact, the intestinal microbiota exerts a continuous influence on the immune system, especially when intestinal permeability and bacterial translocation are increased, as occurs in NAFLD [151,265].

Pathogen-associated molecular patterns (PAMPs), such as lipopolysaccharide (LPS) from Gram-negative bacteria, bind to Toll-like receptors (TLRs) expressed on epithelial cells and cells belonging to the innate immune system, modulating the inflammatory response against exogenous antigens [266,267].

Some preclinical studies have highlighted a marked involvement of TLR4 and TLR9 in the development of steatosis, inflammation, and fibrosis. Indeed, TLR4- or TLR9-deficient mice treated with HFD or a choline-deficient diet were protected from steatosis-related liver failure and inflammation [268].

Therefore, dysbiosis associated with many chronic metabolic diseases, producing continuous immunological stimulation, may promote a condition of chronic low-grade inflammation called meta-inflammation at the basis of many metabolic pathologies [269].

Another pathogenic mechanism seems to be linked to peroxisome proliferator-activated receptors (PPARs), members of the nuclear receptor superfamily, which are able to regulate multiple pathways involved in metabolism and serve as effective targets for the treatment of some metabolic diseases, including NAFLD [152].

In addition to these mechanisms, the gut microbiota interacts with the liver through the so-called “liver–gut axis”, with the involvement of some specific metabolites, including bile acids (BAs), lipopolysaccharides (LPSs) and short-chain fatty acids (SCFAs) [270].

The role of bile acids in the pathogenesis and potential treatment of NAFLD is complex and has been discussed in several reviews [271,272].

Another interesting situation that has been investigated in the animal model concerns the production of SCFAs. Acetic, propionic, and butyric acids are the main products of carbohydrate fermentation by intestinal microorganisms and are produced in terms of about 50–100 mmol/l per day [273]. These SCFAs have effects on energy metabolism, immunity, and adipose tissue expansion [153].

Many of these effects are mediated through binding to G-protein-coupled receptors expressed in the immune system and on gut endocrine cells and adipocytes. The types and amounts of SCFAs synthesized in the gut are modified by carbohydrate intake and dysbiosis, and there are multiple mechanisms by which they may contribute to the progression of NAFLD.

In rats, a diet containing acetic acid, propionic acid, and butyric acid, which mimics cecal fermentation produced from sugar beet fiber (SCFA group), resulted in reduced hepatic cholesterol synthesis and fat content compared to a diet containing whole sugar beet (SBF group) and a control diet without fiber. One explanation for this result may be that cholesterol synthesis in the proximal small intestine was lower in the SCFA group compared to the no-fiber and SBF groups. These studies show that SCFAs derived from fiber fermentation, rather than fiber itself, are responsible for these beneficial effects [274].

SCFA derivatives from the intestine, such as acetate and propionate, provide an energy source for the liver, where they play important roles in hepatic lipogenesis and gluconeogenesis, respectively [275]. Acetate, in particular, can potentially be used as a precursor to cholesterol or fatty acids [276].

Thus, SCFAs account for approximately 30% of the energy delivered to the liver [253].

Thus, changes in the microbiota that favor SCFA production may increase energy delivery to the liver and reduce fecal energy loss. For example, in mice with fatty liver disease, microbial genes responsible for carbohydrate metabolism were overexpressed, resulting in increased SCFA concentrations in the cecum and lower energy content in the feces [221].

In human studies, the role of bacteria in altering energy harvesting is less clear.

In a recent study of 12 lean and 9 obese individuals, no statistically significant differences in fecal energy excretion were found between the two groups, who consumed a 2400 kcal/day and a 3400 kcal/day diet, respectively [138]. However, a large interindividual range in the percentage of calories lost in feces was observed. Unlike other studies, no differences were found in terms of bacterial abundance between lean and obese individuals; bacterial abundance refers to species belonging to the three bacterial phyla Firmicutes, Bacteroides, and Actinobacteria.

A study on adults with NAFLD showed a statistically significant association between the presence of steatohepatitis and a higher percentage of Clostridium coccoides (phylum Firmicutes) and a reduced percentage of Bacteroidetes after adjustment for BMI and dietary fat intake [139]. The importance of Bacteroidetes may lie in their significant contribution to SCFA production and the metabolic potential of the microbiome.

This prospective, cross-sectional study aimed to identify differences in the gut microbiota between adults with biopsy-proven NAFLD (simple steatosis [SS] or non-alcoholic steatohepatitis [NASH]) and living liver donors as healthy controls (HCs). In total, 50 subjects were included: 11 SS, 22 NASH, and 17 HC. A stool sample was collected from each participant. Total bacterial counts and those of Bacteroides/Prevotella (hereafter referred to as *Bacteroidetes*), *Clostridium leptum*, *C. coccoides*, *bifidobacteria*, *Escherichia coli*, and *Archaea* were quantified in stool using PCR. Clinical and laboratory data and dietary data were collected. NASH patients had a lower percentage of Bacteroidetes than both SS and HC (*p* = 0.006) and a higher percentage of fecal *C. coccoides* than SS patients (*p* = 0.04). No differences were found in the remaining microorganisms. Because body mass index (BMI) and dietary fat intake differed between groups (*p* < 0.05), a linear regression adjustment was performed for these variables. The difference in *C. coccoides* was no longer significant after adjustment for BMI and fat intake. However, there was still a significant association between the presence of NASH and a lower percentage of Bacteroidetes even after adjusting for these variables (*p* = 0.002; 95% confidence interval = −0.06 to −0.02). The study concluded that there is an inverse and independent association between diet/BMI, the presence of NASH, and the percentage of Bacteroidetes in stool, suggesting that the gut microbiota may play a role in the development of NAFLD.

For example, a 20% decrease in fecal Bacteroidetes is associated with a corresponding increase in Firmicutes with a 150 kcal increase in energy balance from the diet [138].

The cohort study by Jumpertz R. et al. [139] analyzed the dynamic changes in gut microbiota during the intake of diets varying in caloric content (2400 vs. 3400 kcal/day) by pyrosequencing bacterial 16S ribosomal RNA (rRNA) genes in the feces of 12 lean and 9 obese individuals and measuring calories ingested and excreted in feces using a bomb calorimeter. Altering nutrient load induced rapid changes in the gut microbiota. These changes were directly correlated with fecal energy loss in lean individuals, such that a 20% increase in Firmicutes and a corresponding decrease in Bacteroidetes were associated with an increase in energy harvest of ≈150 kcal. A high degree of overfeeding in lean individuals was accompanied by a greater fraction of fecal energy loss.

These results show that nutrient load is a key variable that can influence gut bacterial community structure on short time scales. Furthermore, the observed associations between gut microbes and nutrient absorption indicate a possible role of the human gut microbiota in regulating nutrient absorption.

Such a change can occur in as little as 3 days of hypercaloric diet, suggesting a very dynamic response to caloric intake in the microbiota composition [277].

Animal studies have shown that high-fat-diet-induced hepatic steatosis is associated with dysbiosis and increased intestinal permeability, with translocation of LPS from Gram-negative bacilli [278]. The relationship between intestinal permeability and NAFLD was further highlighted by the finding in a high-fat dietary model that TNBS (2,4,6-trinitrobenzenesulfonic acid)-induced colitis increased circulating LPS levels and worsened steatohepatitis [277]. LPS has effects not only directly on the liver and intestine but also on the entire metabolism: low doses of LPS chronically administered subcutaneously compromise fasting glycemia and insulin, as well as hepatic insulin sensitivity, cause an increase in visceral, subcutaneous, and hepatic fat, and increase the number of adipose tissue macrophages [278]. From here, we can deduce the mechanisms through which translocated microbial products could contribute to the pathogenesis of NAFLD. At the hepatic level, Toll-like receptors (TLRs) are capable of recognizing intestinal bacterial products (LPS) on Kupffer liver cells, and stellate cells are more stimulated in cases of dysbiosis, and therefore, with increased translocations of these substances, there can be greater stimulation of pro-inflammatory cells and pro-fibrotic pathways through a series of cytokines, including IL-1, IL-6, and TNF71 [279].

In a 2009 study, intestinal permeability was compared between patients with biopsy-proven NAFLD, healthy volunteers, and patients with untreated celiac disease (as a model of change in intestinal permeability). NAFLD patients had significantly increased intestinal permeability (measured by urinary excretion of chromium-51-radiolabeled EDTA) compared to healthy volunteers, although it was lower than in patients with untreated celiac disease. Furthermore, in NAFLD patients, both intestinal permeability and the prevalence of small intestinal bacterial overgrowth correlated with the severity of steatosis but, interestingly, not with steatohepatitis [141].

In another human study, plasma IgG levels against endotoxins were increased histologically in human NASH and increased progressively with the degree of NASH. This finding suggests a relationship between chronic exposure to endotoxins and the severity of human NASH in which increased permeability leads to endotoxemia, which in turn triggers the inflammatory cytokine response and insulin resistance [280].

Regarding the composition of the microbiota, some studies correlate the severity of the disease with a specific microbial signature: proteobacteria are consistently increased in steatosis and non-alcoholic steatohepatitis [252]. Enterobacteriaceae, including *Escherichia coli* and *Shigella*, *Bacteroides*, and *Ruminococcus* are indeed increased in patients with moderate–severe fibrosis, while *Faecalibacterium prausnitzii* and Prevotella are reduced [281].

In fact, invasion of oral bacteria (such as Prevotella or Veillonella) into the distal intestine has been observed in cirrhosis [252]. A recent study by Oh et al. identified some bacteria and bacterial metabolic signatures that independently predict NAFLD-related cirrhosis. *Veillonella* spp., *Enterobacteriaceae*, and *Acidaminococcus* were positively correlated with the severity of liver fibrosis, while Eubacterium spp. and *Faecalibacterium prausnitzii* showed opposite trends; furthermore, tryptophan and related metabolites such as indole and kynurenic acid were altered in NAFLD-related cirrhosis, with an overall increase in stool tryptophan levels [282].

*Faecalibacterium prausnitzii* is reduced not only in cirrhosis but also in other diseases, including diabetes, obesity, and irritable bowel syndrome.

Bacterial signatures (*Clostridium* and *Lactobacillus*) overlap between NAFLD and metabolic disorders (type 2 diabetes mellitus). However, there are discrepancies between different bacterial signatures in different studies. This may be related to heterogeneity related to different geographic regions, ethnicity, population characteristics, microbiome sequencing tools, NAFLD diagnostic tools, disease spectrum, drug use, and circadian rhythm [252].

In conclusion, it has been shown that increased intestinal barrier permeability can increase the concentration of pro-inflammatory substances, such as LPS, which may contribute to the pathogenesis of NAFLD, especially through immune-mediated mechanisms. These mechanisms seem to be enhanced by the predominance of Proteobacteria, especially Enterobacteriaceae.

From these findings, modulation of the intestinal microbiota appears crucial in the future treatment of NAFLD/NASH.

As there is currently no approved pharmacotherapy in NAFLD, existing recommendations for patients focus on lifestyle modification strategies. Such interventions involve weight reduction and/or increased physical activity, which can reduce hepatic lipids and improve glycemic control and insulin sensitivity [283]. The primary goal of diet and exercise involves weight loss of 7–10%, which is correlated with improved liver histology [284].

A recent review (2023) highlights how, among the concomitant factors that promote systemic and hepatic inflammation involved in the pathogenesis of NAFLD, the gut–liver axis seems to have a strong impact in promoting NAFLD and in the progression of the wide spectrum of its manifestations. Among the effective strategies for modulating the intestinal microbiota nutrition is among the most powerful tools: the Western diet negatively affects intestinal permeability and the composition and functionality of the intestinal microbiota, leading to pathobionts, while a Mediterranean-type diet favors the proliferation of beneficial bacteria for health, with a favorable impact on glycolipid metabolism and hepatic inflammation [147].

Studies recommend avoiding simple sugars (especially fructose) and processed red meat and increasing the intake of dietary fiber and unsaturated fatty acids. The daily diet should also include products that improve insulin sensitivity and anti-inflammatory bioactive substances, such as PUFA, MUFA, ginger extracts, flaxseed, and green tea, as demonstrated in recent years [285].

The efficacy of omega-3 PUFA in NAFLD has been studied by He et al. [286]. In their meta-analysis of seven randomized controlled trials (RCTs) involving 442 patients, omega-3 PUFA supplementation (2 to 6.8 g/day for 6 to 12 months depending on the study) positively affected ALT, TC, TG, and HDL and led to reductions in AST, GGT, and LDL.

Similar findings were reported in the meta-analysis by Musa-Veloso et al. [287], which demonstrated that omega-3 PUFAs (EPA: minimum daily dose: 178 mg; maximum: 4626 mg; DHA: minimum daily dose: 200 mg; maximum 2520 mg) were useful in the dietary management of patients with NAFLD: omega-3 PUFA supplementation improved 6 of 13 metabolic outcomes included (TC, LDL, HDL, TG, HOMA-IR, and BMI) as well as ALT and GGT (but not AST), liver lipid content (assessed by MRI), and steatosis.

The same positive result of omega-3 PUFA administration (0.8–13.7 g/day; for 8–12 months) on the decrease in hepatic fat and aminotransferase levels was published by Parker et al. [146].

A randomized, double-blind, placebo-controlled trial included 60 patients with NAFLD and metabolic syndrome who were treated for 12 months with n-3-PUFA (3.6 g/day of n-3-PUFA) or with a placebo. During the 1-year follow-up, patients underwent periodic clinical and laboratory examinations, measurements of liver stiffness, and liver magnetic resonance imaging. After 12 months of n-3-PUFA administration, a significant decrease in serum GGT activity was recorded compared to the placebo group (2.03 ± 2.8 vs. 1.43 ± 1.6; *p* < 0.05). Although no significant changes were recorded in anthropometric parameters, a significant correlation was observed between the reduction in liver fat after 12 months of treatment and weight reduction; furthermore, this effect was clearly enhanced by treatment with n-3-PUFA (*p* < 0.005). Furthermore, n-3-PUFA treatment led to beneficial changes in plasma lipid profile [146].

However, the results of the studies considered are not always consistent with each other. This depends on the type of supplement provided, the placebo (for example, in some cases olive oil was used), the total treatment time, and the characteristics of the subjects treated (some were overweight).

A diet enriched with MUFA and PUFA is able to increase the Bacteroidetes/Firmicutes ratio and the amount of *Bifidobacteria* and *Akkermansia muciniphila*. On the contrary, saturated fatty acids stimulated the growth of *Bilophila* and *Faecalibacterium prausnitzii* and caused a reduction in the number of *Bifidobacterium*, *Bacteroidetes*, *Bacteroides*, *Prevotella*, and *Lactobacillus* ssp. [142,288].

The data available to date suggest that modifying the type of fat consumed with the diet and increasing the percentage of non-digestible carbohydrates may be effective in modulating the intestinal microbiota, reducing hepatic lipids, and improving risk factors associated with NAFLD. However, further studies are needed to specifically and thoroughly evaluate the impact of diet on the gut microbiota, particularly in patients with NAFLD [154].

Again, with regard to nutrition, several studies have demonstrated a relationship between a high protein intake, especially of animal origin, and the risk of NAFLD [143,144].

Higher consumption of animal protein compared to plant protein may also influence bacterial enterotypes in the gut. In the past, some studies have shown that a high intake of animal protein was associated with an increase in bile-tolerant anaerobes, such as *Bacteroides*, *Alistipes,* and *Bilophila* [111,289].

A small number of studies have comprehensively assessed the impact of dietary protein, in terms of quantity and quality, on NAFLD and correlations with the intestinal microbiota, not always leading to homogeneous results. Therefore, it is not yet possible to draw solid conclusions in this regard, and the need for further research in this area is recognized.

#### Probiotics

Scientific research has shown that probiotic supplementation can reverse intestinal dysbiosis and positively influence liver function parameters and lipid and carbohydrate metabolism and reduce inflammation [156], as highlighted in the review by Perumpail, B.J. et al. [156] This review examines 12 studies on a total of 502 patients who were administered probiotics in different combinations (*Lactobacillus bulgaricus* + *Streptococcus thermophilus*; *Lactobacillus acidophilus* + *Lactobacillus casei* + *Lactobacillus rhamnosus* + *Lactobacillus bulgaricus* + *Bifidobacterium brevis* + *Bifidobacterium longum* + *Streptococcus thermophilus*; *Lactobacillus plantarum* + *Lactobacillus bulgaricus* + *Lacto bacillus acidophilus* + *Lactobacillus rhamnosus* + *Bifidobacterium bifidum*; *Lactobacillus acidophilus* + *Bifidobacterium lactis*; *Lactobacilli*, *Bifidobacteria* + *Streptococcus thermophilus*; *Bifidobacterium* + *Lactobacillus* + *Lactococcus* + *Propionibacterium* + *Acetobacter*) or individually (*Lactobacillus acidophilus*; VSL#3; *Bifidobacterium animalis*) daily for a minimum period of 1 month to a maximum of 6.

Numerous experimental studies have demonstrated the therapeutic effects of probiotics in animal models of NAFLD [290]. It was observed that probiotic intake (a 0.6 g/kg/day compound containing six *Lactobacillus* strains and three *Bifidobacterium* strains combined with 15 g/100 g of the prebiotic galacto-oligosaccharide, GOS) reduced body weight and visceral and total fat, modulated the gut microbiota (increased TM7 phylum and decreased *Verrucomicrobia phylum*), modified SCFA level, and inhibited lipid deposition and chronic metabolic inflammation in HFD-fed rats.

The positive influence of probiotics (*Lactobacillus johnsonii* BS15, 2 × 10^7^ CFU/0.2 mL or 2 × 10^8^ CFU/0.2 mL) on gut microbiota, endotoxemia, intestinal permeability, inflammation, and oxidative stress in HFD-treated mice was also observed by Xin et al. [291].

Similarly, the positive influence of multispecies probiotic therapy using VSL#3 (*Lactobacillus plantarum*, *Lactobacillus delbrueckii*, *Lactobacillus casei*, *Lactobacillus acidophilus*, *Bifidobacterium breve*, *Bifidobacterium longum*, *Bifidobacterium infantis*, and *Streptococcus thermophilus*) on the progression of NAFLD has been demonstrated in several animal studies [292,293].

Positive results from experimental studies in the animal model have led to the use of probiotics in patients with NAFLD/NASH. Studies have shown that probiotic supplementation could improve hepatic steatosis and positively modulate metabolic parameters typical of NAFLD [157]. Interventions to date have predominantly involved the use of multispecies compounds with/without the addition of prebiotic substances (in particular combinations of *Lactobacilli*, *Bifidobacteria*, and *Streptococci*; VSL# 3) taken for 8–24 weeks [156].

Studies indicate that probiotics had a beneficial effect in patients with NAFLD and its subset NASH. Results varied between studies, but there was evidence demonstrating improvement in liver enzymes, liver inflammation, hepatic steatosis, and fibrosis. No major adverse effects were noted.

In conclusion, although probiotics have been used for decades, they have only recently been explored as a treatment in the context of NAFLD and NASH. Preliminary data, as seen in several studies, have been encouraging, as they suggest that either probiotics would delay and reverse dysbiosis or they could improve liver inflammation, histology, and liver function as measured by biochemical markers, as well as in liver biopsy specimens. Theoretically, probiotics can be used alone or in combination with other therapies targeting NAFLD. However, potential interactions with other agents need to be investigated. Despite these promising emerging data, sufficient evidence is lacking to recommend their clinical use. Further studies are needed to clarify the role of probiotics.

Prebiotics and synbiotics may also affect steatosis in NAFLD/NASH. The number of studies regarding prebiotic interventions in humans with NAFLD is still limited compared to probiotics; however, their results are promising.

Non-digestible carbohydrates are effective in modulating the gut microbiota and maintaining a healthy gastrointestinal system. However, the impact of a prebiotic-rich diet on the gut microbiota in NAFLD is not fully understood [139].

Arabinoxylan and chitin-glucan have been shown to be effective in modulating the gut microbiota by increasing bifidobacteria and restoring the abundance of Bacteroides—*Prevotella* spp., *Roseburia* spp., and Clostridium cluster XIVa—that are reduced following a high-fat diet. These changes in the gut microbiota were also supported by reductions in body fat, liver lipids, serum and liver cholesterol, and insulin resistance, independent of calories consumed [154].

There is also evidence in animal models that, in the presence of high-fat diets, chitosan and arabinoxylan are able to increase fat, bile acids, and cholesterol in feces. These results suggest that non-digestible carbohydrates are able to modulate the gut microbiota, even in the presence of a high-fat diet, potentially by binding to fat/cholesterol or by inhibiting pancreatic lipases, as reported in the review by Neyrinck [294].

A human study demonstrated that beta-glucan intake (1.5 g) for 12 weeks reduced BMI, AST, ALT, TC, and TG and improved liver function [155]. Oat beta-glucans (1.5 mg/kg m^3^/day) also prevented metabolic disorders, hepatic steatosis, and inflammation in LPS-induced NASH in animal models [295,296]. Another study demonstrated that the administration of 10 g of psyllium (Plantago ovata) was associated with a reduction in ALT and AST, waist circumference, and caloric intake in obese patients with NAFLD.

Subsequent studies have shown that the positive influence of prebiotics on the course and progression of NAFLD derives not only from the outcome of the improvement of metabolism but also from the modulation of the composition of the intestinal microbiota also through their capacity to increase SCFAs, in particular butyrate [297].

This was highlighted in an animal model study using gnotobiotic C3H/HeOuJ mice colonized with a simplified human microbiota. Mice were fed a high-fat diet supplemented with 10% cellulose (non-fermentable) or inulin (fermentable) for 6 weeks. Feeding the inulin diet resulted in increased diet digestibility and reduced fecal energy compared to the cellulose diet with no difference in food intake, suggesting increased intestinal energy extraction from inulin. However, no increase in body fat/weight was observed. The additional energy provided by the inulin diet resulted in increased bacterial overgrowth in this group. Inulin supplementation resulted in significantly elevated total SCFA concentrations in the cecum and portal system, with a reduced cecal acetate/propionate ratio. Hepatic expression of genes involved in lipogenesis (Fasn, Gpam) and fatty acid elongation/desaturation (Scd1, Elovl3, Elovl6, Elovl5, Fads1, and Fads2) was decreased in inulin-fed animals. Accordingly, the composition of phospholipids in plasma and liver changed between the different feeding groups. Concentrations of omega-3 and odd-chain fatty acids increased in inulin-fed mice, while omega-6 fatty acids decreased.

Overall, these data indicate that during this short-term feeding, inulin has mainly positive effects on lipid metabolism, which could lead to beneficial effects during the development of obesity in long-term studies.

Several meta-analyses on the efficacy of synbiotic therapy (combination of prebiotics and probiotics) in NAFLD have been published in recent years. Loman et al. [158] in 2018 analyzed 25 RTCs (9 used prebiotics, 11 used probiotics, and 7 used synbiotics; n = 1309 patients with NAFLD) and concluded that microbial therapies significantly reduced AST and ALT, but not CRP. They noted that serum TC and LDL levels were different in studies using prebiotics, probiotics, and synbiotics.

In this meta-analysis, the dose and characteristics of treatments were variable within the three classes of prebiotics, probiotics, and synbiotics, and especially within the prebiotic class. For prebiotics, treatments included beta-glucan-enriched cereals, psyllium exocarp, fructo-oligosaccharides (FOSs), xylooligosaccharides (XOSs), chicory inulin, and fiber extracts (e.g., *Chlorella vulgaris*). For the synbiotic study group, the primary prebiotic source was FOS (n¼5 of 7 studies); the other two studies used inulin. Similar to prebiotic studies, probiotic studies were highly divergent in the species of microorganisms used (*Lactobacillus reuteri*, *Lactobacillus bulgaricus*, *Lactobacillus acidophilus*, *Lactobacillus rhamnosus*, Lactobacillus lactis, *Lactobacillus casei*, *Lactobacillus plantarum*, *Lactobacillus sporogenes*, *Lactobacillus delbrueckii*, *Bifidobacterium bifidum*, *Bifidobacterium longum*, *Bifidobacterium infantis*, *Bifidobacterium breve*, and *Streptococcus thermophilus*), and most studies integrated multiple organisms. *Lactobacillus acidophilus* was the most commonly used species in both probiotic and synbiotic treatments.

Regarding effects on lipid profile, individual prebiotic and probiotic treatments did not reduce CT, while synbiotics did. All three treatment types combined reduced LDL-C by 4.52 mg/dL (95% CI, 8.87 to 0.17; *p* < 0.001). Analysis by treatment type showed that prebiotics reduced LDL-C (6.67 mg/dL; 95% CI, 12.03 to 1.30; *p* < 0.001), but probiotics and synbiotics did not.

Similarly, Khan et al. [159], based on 12 RCTs of probiotics/synbiotics (n = 748 patients with NAFLD), stated that probiotics/synbiotics were associated with significant improvements in ALT, AST, and liver fibrosis (assessed by elastography). Positive changes in CRP, LDL, TG, and TC were observed only for synbiotics. Improvements in ALT and AST associated with reduced steatosis and liver stiffness after probiotic/synbiotic supplementation was observed in the meta-analysis published by Sharpton et al. and Liu et al. [160].

Treatments ranged from a minimum of 4 to a maximum of 28 weeks and included monospecies probiotics (*Lactobacillus acidophilus* and *Lactobacillus rhamnosus*), multispecies probiotic compounds (*Lactobacillus bulgaricus* + *Streptococcus thermophilus*, Prokid, Symbiter, Lactocare, Probiotic Yogurt, VSL#3, and Webber Naturals), mono/bi-species probiotic + FOS (*Bifidobacterium animalis* + inulin, *Lactobacillus reuteri* + inulin, *Bifidobacterium longum* + FOS, *Bifidobacterium longum* + *Lactobacillus acidophilus* + inulin), and a mix of unspecified probiotics and FOS.

In conclusion, supplementation with prebiotics and synbiotics could improve hepatic steatosis, liver function, some parameters of metabolic syndrome, inflammatory markers, and liver stiffness. However, there is a lack of high-quality multicenter RCTs involving populations of various origins. Therefore, further studies are needed to draw a definitive conclusion on the effect of supplementation of these compounds in patients with NAFLD.

Several meta-analyses have found positive effects on ALT and AST through the use of synbiotics and prebiotics, mainly represented by fructo-oligosaccharides [159,160].

In a meta-analysis, four studies with 235 participants using probiotics and synbiotics demonstrated a reduction in liver stiffness (an index of inflammation and fibrosis) measured by elastography. In the same meta-analysis, six studies with 384 participants using probiotics or synbiotics were more likely to improve steatosis in patients with moderate-to-severe fatty liver disease [160].

In patients with biopsy-proven NASH, 24-week treatment with synbiotics (*Bifidobacterium longum* and fructo-oligosaccharides) combined with lifestyle modification resulted in reduction in serum AST and histological improvement in NASH compared to lifestyle modification alone [298].

In relation to the European Society for Clinical Nutrition and Metabolism (ESPEN) guidelines on gastrointestinal diseases in obese patients, this document systematically analyzed randomized clinical trials (RCTs) evaluating the use of prebiotics, probiotics, or synbiotics in the treatment of NAFLD in obese adults in several recent meta-analyses. Most of the analyzed RCTs were based on the administration of probiotics and reported consistent positive effects on liver enzymes [299].

In conclusion, the gut microbiota plays a crucial role in the development of liver steatosis, particularly in conditions such as non-alcoholic fatty liver disease (NAFLD). This relationship is influenced by dietary factors, microbial composition, and metabolic interactions. Considering dietary fat composition, the type of dietary fat consumed significantly affects gut microbiota composition. Diets high in saturated fatty acids (SFAs) can lead to dysbiosis, characterized by a decrease in beneficial bacteria and an increase in pathogenic species. This shift promotes increased intestinal permeability, allowing bacterial components like lipopolysaccharides (LPSs) to enter the bloodstream and reach the liver, where they can trigger inflammation and steatosis. As regards microbial metabolites, gut bacteria produce short-chain fatty acids (SCFAs) through the fermentation of dietary fibers. SCFAs have anti-inflammatory properties and play a role in regulating metabolism. A healthy gut microbiome can enhance SCFA production, which may protect against liver fat accumulation. Conversely, dysbiosis can lead to reduced SCFA levels and increased inflammation, contributing to steatosis. As far as immune system modulation is concerned, the gut microbiota influences immune responses that affect liver health. Dysbiosis can alter T-cell populations and promote systemic inflammation, which is linked to the progression of liver disease. Finally, considering bile acid metabolism, the gut microbiota also regulates bile acid metabolism, which is vital for lipid digestion and absorption. Changes in bile acid composition due to altered microbiota can impact liver function and contribute to steatosis.

Recent studies have explored the potential of probiotics as a therapeutic option for managing NAFLD, focusing on their effects on liver function, metabolic profiles, and inflammation.

The mechanisms of action of prebiotics involved the gut–liver axis and regulation of lipid metabolism. Probiotics interact with the gut microbiota, which plays a crucial role in liver health. They can enhance the production of short-chain fatty acids (SCFAs) like butyrate, which have anti-inflammatory properties and promote fatty acid oxidation. Moreover, certain probiotics have been shown to influence lipid metabolism by regulating gene expression related to fat storage and oxidation, thereby reducing hepatic steatosis. Specifically, for the gut–liver axis and microbiota dysbiosis, there is bidirectional communication: the gut–liver axis involves a complex interplay where metabolites and signals from the gut microbiota affect liver function.

This communication occurs via the portal vein, biliary tract, and systemic circulation, establishing a reciprocal relationship that can impact liver health and disease progression. Dysbiosis (an imbalance in gut microbiota composition) has been linked to the development and progression of NAFLD. Alterations in microbial populations can lead to increased intestinal permeability, allowing harmful substances to enter the bloodstream and trigger liver inflammation and metabolic dysfunction.

Considering efficacy of probiotics in NAFLD management, there is various clinical evidence, such as liver enzyme reduction, metabolic improvements, and inflammation. As far as liver enzyme reduction is concerned, probiotic supplementation has shown promising results in reducing liver enzyme levels, specifically alanine aminotransferase (ALT) and aspartate aminotransferase (AST), in patients with NAFLD. Various meta-analyses indicated significant reductions in these enzymes, suggesting improved liver function. Regarding metabolic improvements, probiotics appear to positively influence various metabolic parameters, including insulin sensitivity and lipid profiles. For instance, a systematic review highlighted that probiotic interventions could lower fasting blood glucose and triglyceride levels. Finally, as far as inflammation is concerned, probiotics may help mitigate inflammation associated with NAFLD by modulating gut microbiota and enhancing intestinal barrier integrity. This modulation can potentially reduce the translocation of harmful bacteria and endotoxins that exacerbate liver inflammation. Combining probiotics with lifestyle interventions, such as exercise, has been found to further enhance their benefits. Studies suggest that this combination can lead to greater improvements in liver enzyme levels and overall metabolic health compared to exercise alone.

The mechanisms of probiotic action are restoration of microbiota balance and metabolite production. Regarding the restoration of microbiota balance, probiotics can help restore a healthy balance of gut microbiota by increasing beneficial bacteria such as *Lactobacillus* and *Bifidobacterium* while reducing pathogenic species. This restoration can improve intestinal barrier function and reduce inflammation, both of which are critical in managing NAFLD. Considering metabolite production, probiotics produce beneficial metabolites, including short-chain fatty acids (SCFAs), which have anti-inflammatory effects and promote fatty acid oxidation. These metabolites can alleviate hepatic steatosis and improve metabolic profiles.

Specific strains have shown promise in clinical trials, with *Lactobacillus* and *Bifidobacterium* being the most commonly studied. These strains have demonstrated potential in improving hepatic steatosis and metabolic parameters linked to NAFLD.

Specifically, considering *Lactobacillus* species, strains such as *Lactobacillus rhamnosus* GG and *Lactobacillus plantarum* have been widely studied. Regarding *Lactobacillus rhamnosus* GG, this strain has demonstrated effectiveness in reducing liver inflammation and improving liver function markers in clinical settings. Regarding *Lactobacillus plantarum*, this strain has been shown to help alleviate symptoms of NAFLD and improve metabolic profiles. Regarding *Bifidobacterium* species, strains such as *Bifidobacterium bifidum* and *Bifidobacterium longum* have been associated with improvements in liver enzyme levels and reductions in liver fat content. Other notable strains are *Lactococcus lactis*, *Streptococcus thermophilus*, and *Bacillus coagulans*, which have also been mentioned in studies, although less frequently than *Lactobacillus* and *Bifidobacterium*.

Regarding recommendations for use, probiotics are often recommended at doses of once or twice daily, with longer durations (over 12 weeks) yielding better results. Moreover, combining probiotics with lifestyle changes, such as dietary modifications and exercise, can enhance their effectiveness in managing NAFLD.

## 5. Conclusions: Management of Chronic Metabolic Disorders with Probiotics

In conclusion, a condition of dysbiosis, or in general, alteration of the intestinal microbiota, could be implicated in the development of metabolic disorders through different mechanisms, mainly linked to the release of pro-inflammatory factors. Several studies have already demonstrated the potential of using probiotics and prebiotics in the treatment of this condition, detecting significant improvements in the specific symptoms of metabolic diseases. These findings reinforce the hypothesis according to which a condition of dysbiosis can lead to a generalized inflammatory picture with negative consequences on different organs and systems. Moreover, this review confirms that the beneficial effects of probiotics on metabolic diseases are promising; however, their use is not without limitations and risks, particularly in specific populations and conditions. Probiotics are not standardized, leading to significant variability in their efficacy across different products and strains. This inconsistency makes it challenging to determine which probiotics are most effective for metabolic control, particularly in conditions like T2D and obesity [27].

The use of probiotics can pose safety risks, especially in immunocompromised individuals or those with severe intestinal barrier dysfunction. For instance, patients with active diseases that involve mucosal ulceration may experience increased risks of bacterial translocation and sepsis when probiotics are administered [300].

Clinical trials have indicated higher mortality rates and complications such as bowel ischemia among critically ill patients treated with probiotics compared to those receiving placebo [300].

Thus, probiotics are contraindicated for critically ill patients or those with significant comorbidities.

There is a pressing need for more standardized research to clarify how different probiotic strains affect metabolic health across diverse demographics. The current literature suggests that while probiotics can positively impact obesity-related parameters, their effects may vary significantly based on age, gender, and individual health profiles.

In conclusion, while probiotics show promise in managing metabolic pathologies, their use is limited by variability in efficacy, safety concerns in vulnerable populations, unclear mechanisms of action, strict exclusion criteria in research studies, and a need for more standardized investigations. These factors must be carefully considered when evaluating the role of probiotics in metabolic health management.

### 5.1. Metabolic Syndrome

*Lactobacillus acidophilus*, *Lactobacillus casei*, *Bifidobacterium animalis* subsp. *lactis*, and *Akkermansia muciniphila* show promise in managing metabolic syndrome, even if further research is needed to fully understand their effects and optimal usage in clinical settings. Future studies should focus on the efficacy of these strains in diverse populations and their integration into dietary strategies for MetS management.

### 5.2. Diabetes

Research indicates that certain probiotic strains may be particularly beneficial for managing type 2 diabetes mellitus (T2DM). The strains that have shown promise based on clinical studies and meta-analyses are *Lactobacillus acidophilus*, *Lactobacillus plantarum*, *Bifidobacterium lactis*, *Lactobacillus rhamnosus* GG, and *Saccharomyces boulardii*.

Considering *lactobacillus acidophilus*, this strain can improve epithelial barrier function and reduce inflammation, which may help regulate glucose and lipid metabolism. Studies have indicated that yogurt containing this strain can enhance the lipid profile in T2DM patients.

Regarding *Lactobacillus plantarum*, this strain is involved in glucose metabolism regulation and has shown potential in reducing low-grade inflammation associated with insulin resistance. While human trials are limited, animal studies suggest that *Lactobacillus plantarum* can mitigate hyperglycemia and improve insulin sensitivity.

Regarding *Bifidobacterium lactis*, this strain has been linked to improvements in metabolic parameters, including reductions in fasting blood glucose (FBG) and glycated hemoglobin (HbA1c) levels. It is often included in probiotic multistrain formulations aimed at enhancing glycemic control.

Regarding *Lactobacillus rhamnosus* GG, this strain has been associated with improved glycemic control and may aid in reducing inflammation related to diabetes. It has been highlighted in studies as a beneficial adjunct therapy for T2DM management.

Considering *Saccharomyces boulardii*, although primarily studied in animal models, it shows promise in improving metabolic health and gut microbiota composition. Research suggests that it may help regulate glucose levels, although more human studies are needed to confirm these effects.

The use of probiotics has been linked to improvements in metabolic profiles, including reductions in HbA1c, fasting blood glucose, and insulin resistance (HOMA-IR). However, efficacy varies depending on strains, dosage, and individual factors. Further research is needed to establish standardized guidelines, but incorporating probiotics into the diet may support glycemic control alongside conventional diabetes treatments.

### 5.3. Prediabetes

The mechanisms by which probiotics may regulate blood glucose homeostasis in prediabetes include improving insulin resistance, regulating intestinal permeability, modulating metabolic regulation, and altering the gut microbiota composition. Considering how long it takes to see improvements in blood sugar with probiotics, the studies suggest that it may take a few months of consistent probiotic use before seeing significant, lasting improvements in blood glucose levels. Specifically, a meta-analysis found that taking probiotics for 8 weeks or longer may enhance the effect on glucose metabolism. The key is to keep probiotic intake as regular as possible and review trends in your blood glucose data daily and over time. Continuous glucose monitoring can provide ongoing data to assess the impact of probiotics on your blood sugars. However, it is important to note that probiotics should be considered one tool as part of an overall health strategy. Relying exclusively on probiotics to manage blood sugars is not recommended. One should continue to eat balanced meals, exercise regularly, and stay hydrated in addition to taking probiotics.

### 5.4. Obesity

The role of the gut microbiota in the development and management of obesity is emerging as a crucial field of research. Scientific evidence shows that obese people tend to present a reduced microbial diversity and an increased Firmicutes/Bacteroidetes ratio, with a greater capacity to extract energy from the diet. Preclinical and clinical studies have shown that specific microbial strains, such as *Akkermansia muciniphila, Christensenellaceae* and some *Lactobacillus*, can influence energy metabolism and contribute to the regulation of body weight.

Randomized clinical trials (RCTs) in humans are still limited (currently 10 studies), but have explored different probiotic and synbiotic strains. Of these, four studies have evaluated the efficacy of single strains, such as *Lactobacillus rhamnosus*, *Lactobacillus gasseri*, *Bifidobacterium breve* and *Lactobacillus plantarum*. Another three studies have investigated combinations of strains, such as *Lactobacillus rhamnosus* with *Bifidobacterium longum* and *Lactobacillus bulgaricus* with *Streptococcus thermophilus*. Finally, three studies have considered the efficacy of synbiotics that combine prebiotics such as fructo-oligosaccharides (FOSs) with probiotics, showing promising results in improving the metabolic profile and reducing adiposity.

Despite the encouraging results, research suggests that the efficacy of probiotics in the treatment of obesity is strain-dependent and varies according to individual conditions and diet. Microbiota modulation strategies, including probiotics, prebiotics, and fecal transplantation, represent a potential therapeutic for the management of obesity and its complications. However, further studies are needed to confirm the efficacy of these strategies on a large scale and to better understand the mechanisms involved.

### 5.5. Hyperhomocysteinemia

Probiotics may influence homocysteine metabolism by enhancing the production of B vitamins, which are crucial for homocysteine breakdown. Certain probiotic strains have been shown to produce vitamins, such as folate and vitamin B12, which are directly involved in the metabolism of homocysteine, potentially leading to lower plasma levels of this amino acid. Moreover, probiotics help restore a balanced gut microbiota, which can be disrupted in conditions like obesity and metabolic syndrome. This dysbiosis may contribute to increased homocysteine levels. By re-establishing a healthy microbial community, probiotics can improve metabolic processes, including those involved in homocysteine metabolism. Probiotic supplementation has been associated with decreased levels of inflammatory markers and oxidative stress, both of which can exacerbate homocysteine levels. For instance, a study indicated that multispecies probiotics reduced homocysteine levels while also lowering tumor necrosis factor-alpha (TNF-α) and improving total antioxidant status in obese women. This suggests that probiotics may mitigate the inflammatory processes that can lead to elevated homocysteine.

Finally, probiotics have been shown to improve lipid profiles by reducing total cholesterol and triglyceride levels, which are often associated with cardiovascular risk. This improvement in lipid metabolism may indirectly influence homocysteine levels, as lipid abnormalities can be linked to dysregulated homocysteine metabolism.

### 5.6. Dyslipidemia

Alterations in the gut microbiota induced by a high-fat diet can negatively affect the composition of the microbiota, reducing beneficial bacteria such as *Bifidobacterium* and *Lactobacillus* and favoring the proliferation of pathogenic genera such as *Bilophila wadsworthia* and *Enterobacteria*. These alterations are significantly related to the development of dyslipidemia and contribute to a vicious cycle that aggravates metabolic and cardiovascular conditions. Several studies have explored the potential of probiotics in the treatment of dyslipidemia, with significant improvements in LDL cholesterol, HDL cholesterol, and triglyceride levels, in addition to the reduction in systemic inflammation. Recent meta-analyses [122,123] have confirmed that probiotic supplementation, particularly the *Lactobacillus* and *Bifidobacterium* genera, reduces total and LDL cholesterol levels, with more marked effects in hypercholesterolemic subjects than in normocholesterolemic subjects. Among the most effective probiotics, *Lactobacillus reuteri* NCIMB 30242 has been shown to reduce LDL-C levels by approximately 11.6% in hypercholesterolemic adults [122].

Probiotic strains such as *Lactobacillus reuteri*, *Lactobacillus acidophilus*, and *Bifidobacterium lactis* are particularly effective in lowering cholesterol levels in hypercholesterolemic patients. When selecting probiotic products, it is important to consider those that contain these specific strains for optimal results in managing dyslipidemia.

Finally, probiotics have been shown to impact cholesterol levels differently in elderly individuals compared to younger adults, primarily due to variations in baseline cholesterol levels and the physiological changes associated with aging. Probiotic interventions tend to produce more significant reductions in total cholesterol (TC) and low-density lipoprotein cholesterol (LDL-C) in elderly individuals than in younger adults. This is attributed to the generally higher baseline cholesterol levels found in older adults, making them more responsive to probiotic supplementation.

Probiotics seem more effective in lowering cholesterol in the elderly than in younger adults, likely due to higher baseline levels and age-related changes. However, more research and clinical trials are needed to define optimal strains, dosages, and long-term effects for this demographic.

### 5.7. Sarcopenia

A condition of dysbiosis or, in general, of alteration of the intestinal microbiota, due to age or external factors, could be implicated in the development of sarcopenia through different mechanisms, mainly linked to the release of pro-inflammatory factors. Several studies have already demonstrated the potential of using probiotics in the treatment of this condition, detecting significant improvements both in maintaining lean mass and muscle strength. These findings reinforce the hypothesis according to which a condition of dysbiosis can lead to a generalized inflammatory picture, with negative consequences on different organs and systems, including the musculoskeletal one.

RCT studies in humans are still few (currently six studies) but have evaluated numerous strains: two studies evaluated a single strain of *Lactobacillus plantarum* TWK10 (2 × 10^10^ CFU per day) and *Lactobacillus paracasei* PS23 (30 billion colony-forming units); three studies considered an association consisting of 112 billion colony-forming units of *B. longum* DSM 24736, *B. breve* DSM 24732, DSM 24737; *Streptococcus thermophilus* DSM 24731; and lactobacilli (DSM 24735, DSM 24730, DSM 24733, *L. delbrueckii* subsp. bulgaricus DSM 24734). All studies have shown that the intake of probiotics has proven effective in improving muscle mass and strength.

### 5.8. NAFLD

The gut microbiota plays a crucial role in the development of liver steatosis, particularly in conditions such as non-alcoholic fatty liver disease (NAFLD). This relationship is influenced by dietary factors, microbial composition, and metabolic interactions.

Recent studies have explored the potential of probiotics as a therapeutic option for managing NAFLD, focusing on their effects on liver function, metabolic profiles, and inflammation.

Specific strains have shown promise in clinical trials, with *Lactobacillus* and *Bifidobacterium* being the most commonly studied. These strains have demonstrated potential in improving hepatic steatosis and metabolic parameters linked to NAFLD.

Specifically, considering *Lactobacillus* species, strains such as *Lactobacillus rhamnosus* GG and *Lactobacillus plantarum* have been widely studied. Regarding *Lactobacillus rhamnosus* GG, this strain has demonstrated effectiveness in reducing liver inflammation and improving liver function markers in clinical settings. Regarding *Lactobacillus plantarum*, this strain was shown to help alleviate symptoms of NAFLD and improve metabolic profiles. Regarding *Bifidobacterium* species, strains such as *Bifidobacterium bifidum* and *Bifidobacterium longum* have been associated with improvements in liver enzyme levels and reductions in liver fat content. *Lactococcus lactis*, *Streptococcus thermophilus*, and *Bacillus coagulans* have also been mentioned in studies, although less frequently than *Lactobacillus* and *Bifidobacterium*.

Probiotic supplementation has shown promise in managing NAFLD by improving liver function, metabolic profiles, and reducing inflammation. Typically recommended once or twice daily, probiotics yield better results when used for over 12 weeks. Their efficacy is enhanced when combined with lifestyle modifications like dietary changes and regular exercise. However, outcomes can vary based on the probiotic strains, dosages, and individual patient factors. While preliminary studies are encouraging, more robust clinical trials are needed to establish standardized guidelines for probiotic use in NAFLD treatment.

## Figures and Tables

**Table 1:** (**A**) Metabolic syndrome and microbiota: systematic reviews and meta-analyses. (**B**) Probiotics and metabolic syndrome: randomized and non-randomized clinical trials. (**C**) Probiotics and metabolic syndrome: systematic reviews and meta-analyses.

**(A)**								
**Authors**	**Type of Studies**	**Number of Studies and Type of Study**		**Subjects**	**End Point**	**Results**	**Conclusion**	**Strength of** **Evidence**
Portela-Cidade et al. (2015) [32]	Systematic review	35 of a total of 230 articles were included		-	Access the most recent data about the relevance of intestinal microbiota and Toll-like receptor (TLR) expression in the development of hepatic lesions and metabolic syndrome	Early activation of TLRs and its interactions with a dysbiotic intestinal microbiota play a key role in metabolic syndrome	There is evidence in the literature that suggests that innate immunity and intestinal microbiota may be the hidden link in the metabolic syndrome development mechanisms	High
Cheng et al. (2023) [33]	Systematic review	11 RCTs		608	Effects of supplementation with synbiotics and probiotics on MetS parameters	Lowered BMI, LDL-c levels, and fasting blood glucose was achieved, no effect on blood pressure	The literature supports the possibility of using probiotics to help ameliorate Met-S parameters	High
He M, Shi B (2017) [34]	Narrative review	-		-	Review recent studies concerning the role of the gut microbiota in MS modulation	-	-	Medium
Mallappa RH et al. (2012) [35]	Narrative review	-		-	Analize the effects of prebiotics and probiotics on metabolic syndrome in a “pharmaco-nutritional” approach	-	-	Medium
Horvath (2024) [36]	Scoping review	13 studies on diet and the gut microbiome in patients with MetS (total of 961 patients; 26 studies testing probiotics as microbiome modulators in obesity, MetS, and diabetes (total of 4403 patients); 15 studies on prebiotics and the gut microbiome in patients with metabolic syndrome (total of 913 patients); 10 studies on fecal microbiome transplantation in patients with metabolic syndrome (total of 483 patients)		-	Metabolic syndrome parameters, weight-related parameters	No significant effects on metabolic parameters	The results do not favor treatment	High
Hadi A et al. (2021) [37]	Meta-analysis	Ten eligible publications (nine RCTs, n = 344 participants) were included		-	Evaluating the effects of pro-/synbiotic consumption and supplementation in adults (≥18 years) with MetS	Supplementation with pro-/synbiotics reduced total cholesterol (TC) in adults with MetS versus placebo (MD: −6.66 mg/dL, 95% CI: −13.25 to −0.07, *p* = 0.04, I^2^ = 28.8%, n = 7), without affecting weight, body mass index, waist circumference, fasting blood sugar, homeostasis model assessment for insulin resistance, insulin, triglycerides, low-density lipoprotein cholesterol, or high-density lipoprotein cholesterol (*p* > 0.05)	Pro-/synbiotic consumption may be beneficial in reducing TC levels in adults with MetS	High
Chen T et al. (2024) [33]	Meta-analysis	11 RCTs were identified		608 participants in total included	Analyze the effects of probiotics or synbiotics on cardiovascular factors in adults with MetS	The supplementation with probiotics or synbiotics reduced body mass index (*p* < 0.0001), low-density lipoprotein (LDL-c) (*p* = 0.004), and fasting blood glucose (FBG) (*p* = 0.03), but had no beneficial effect on systolic blood pressure (SBP) (WMD = 1.24, 95% CI = [−2.06, 4.54], *p* = 0.46, n = 8) in MetS patients	Supplementation with probiotics or synbiotics can reduce BMI, LDL-c, FBG in patients with MetS	High
**(B)**								
**Authors**	**Type of Studies**	**Population** **Characteristics**	**Type of** **Intervention**	**Duration**	**End Point**	**Results**	**Conclusion**	**Strength of** **Evidence**
Leber B et al. (2012) [38]	Randomized controlled trial	Twenty-eight patients and ten healthy controls were included	Supplementation of 3 × 6.5 × 10⁹ CFU *L. casei* Shirota (probiotic group) per day	3 months	Investigate the effect of *Lactobacillus casei* Shirota on gut permeability, presence of endotoxin and neutrophil function in MetS	PCR and LBP levels slightly but significantly increased after 3 months within the probiotic group. Neutrophil function, TLR expression, and LBP and sCD14 levels were not significantly different between the groups.	*L. casei* Shirota administration in the MetS patients did not have any influence on any parameter tested, possibly due to too-short study duration or underdosing of *L. casei* Shirota.	High
Tripolt NJ et al. (2012) [39]	Randomized controlled trial	30 subjects with metabolic syndrome	Supplementation with a milk drink containing LcS (3 bottles a day, 65 mL, containing LcS at a concentration of 10^8^/mL)	12 weeks	Determine the effects of supplementation with *Lactobacillus casei* Shirota on insulin sensitivity, β-cell function, inflammation, and endothelial dysfunction parameters in subjects with metabolic syndrome	-	No insulin sensitivity index improvements were found, nor in β-cell function.	High
Stadlbauer V et al. (2015) [40]	Randomized controlled trial	28 subjects with metabolic syndrome (13 in treatment group and 15 controls)	Supplementation with a milk drink containing LcS (3 bottles a day, 65 mL, containing LcS at a concentration of 10^8^/mL)	12 weeks	Investigate the effect of *Lactobacillus casei* Shirota (LcS) on gut microbiota composition, gut barrier integrity, intestinal inflammation, and serum bile acid profile in metabolic syndrome	Zonulin and calprotectin were increased in metabolic syndrome stool samples but not influenced by LcS supplementation. Serum bile acids were similar to controls and not influenced by LcS supplementation. Metabolic syndrome is associated with a higher Bacteroidetes/Firmicutes ratio and gut barrier dysfunction but LcS was not able to change this.	No effect of LcS on gut barrier integrity markers	High
Barreto FM et al. (2013) [41]	Randomized controlled trial	24 postmenopausal women with METs divided into treatment and placebo group	80 mL/day of fermented milk with 1.25 × 10^7^ UFC/g of lactobacillus plantarum	3 months	Evaluate the influence of fermented milk with *L. plantarum* in the classical parameters related to MetS, as well as in other parameters related to cardiovascular risk in postmenopausal women	A significant reduction in glucose and homocysteine levels was observed in the treatment group compared with the placebo group (*p* = 0.037 and *p* = 0.019, respectively) but no significative difference was found for total cholesterol, blood lipids, and inflammatory biomarkers.	*L. plantarum* supplementation may have a role in the treatment of metabolic syndrome in postmenopausal women.	High
Bernini LJ et al. (2016) [42]	Randomized controlled trial	51 patients with Mets, 25 receiving placebo, the others (n = 26) receiving *Bifidobacterium Lactis* HN019 supplementation	A daily serving of fermented milk with *Bifidobacterium Lactis* HN019 (80 mL, each dose with 2.72 × 10^10^ colony-forming units)	45 days	Evaluate the effect of consumption of milk containing the probiotic *B. lactis* HN019 on the classical parameters of MetS and other related cardiovascular risk factors	Treatment group showed significant decrement in body mass index (*p* = 0.017), total cholesterol (*p* = 0.009), and low-density lipoprotein (*p* = 0.008) compared with baseline and control group and a significant reduction in pro-inflammatory cytokines.	*B. lactis* HN019 may have a role in treating obesity and reducing blood lipids and some inflammatory markers in patients with MetS.	High
Wastyk HC et al. [43]	Randomized controlled trial	39 adults with elevated Met-S parameters, 23 assigned to the intervention arm, 16 to placebo	Probiotic made of a blend of *Limosilactobacillus reuteri* NCIMB 30242, *Lactiplantibacillus plantarum* UALp-05™, and *Bifidobacterium animalis* subsp. *lactis* B420™	18 weeks	Evaluate the effects of the use of probiotics by themselves to lower Met-S parameters	No significant effects on any of the parameters considered. Data possibly explained by there being responders and non-responders.	Hypothesis of some individuals responding to treatments with probiotics while others being resistant to it could be considered.	High
Kassaian N et al. (2018) [44]	Randomized controlled trial	120 adults with impaired glucose tolerance divided into three groups: treatment with probiotics, treatment with probiotics + prebiotics and placebo	6 g/day of probiotic containing *Lactobacillus acidophilus*, *Bifidobacterium lactis*, *Bifidobacterium bifidum*, and Bifidobacterium longum (1 × 10^9^ for each), or synbiotic comprising the mentioned probiotics with an inulin-based prebiotic, or placebo	24 weeks	Determine the effects of 6 months of ingestion of probiotic or synbiotic on metabolic syndrome indices and gut micro- biota composition	Compared with the placebo, synbiotic supplementation resulted in a more significant reduction in FPG (−6.5 ± 1.6 vs. −0.82 ± 1.7 mg/dL, *p* = 0.01), FIL (−2.6 ± 0.9 vs. −0.8 ± 0.8 µIU/mL, *p* = 0.028), and HOMA-IR (−0.86 ± 0.3 vs. −0.16 ± 0.25, *p* = 0.007), and a significant elevation in the QUICKI (+0.01 ± 0.003 vs. +0.003 ± 0.002, *p* = 0.006). In addition, significant decreases in HbA1C were seen following the supplementation of probiotics and synbiotics compared with the placebo (−0.12 ± 0.06 and −0.14 ± 0.05 vs. +0.07 ± 0.06%, *p* = 0.005 and 0.008, respectively). HOMA-B was not found to be different between or within the three groups.	Glycemic improvement by probiotics and particularly synbiotic supplements in prediabetic individuals has been supported by the current study. However, further studies are required for optimal recommendations in this important area of patient treatment.	High
Yu (2020) [45]	Randomized controlled trial	Obese and insulin resistance patients 25 to 60 y.o	FMT (fecal matter transplant)	12 weeks	Insulin sensitivity, glycemic and lipidic profile	No statistically significant difference	The results do not favor treatment.	High
De Moura (2023) [46]	Randomized controlled trial	Obese (BMI 30–40) women with metabolic syndrome from 18 to 70 y.o	FMT	12 weeks	Insulin sensitivity, glycemic and lipidic profile	No statistically significant difference	The results do not favor treatment.	High
Smits (2018) [47]	Randomized controlled trial	Male metabolic syndrome patients from 21 to 69 y.o	FMT	2 weeks	Vascular injury, glycemic and lipidic profile	No statistically significant difference	The results favor treatment only for glycemic profile at 6 weeks.	High
Kootte (2017) [48]	Randomized controlled trial	Male metabolic syndrome patients from 21 to 69 y.o	FMT	18 weeks	Insulin sensitivity, glycemic and lipidic profile	Insulin sensitivity at 6 weeks: from 25.8 [19.3–34.7] to 28.8 [21.4–36.9] μmol kg^−1^ min^−1^, *p* < 0.05. HbA1c at 6 weeks: 39.5 [36.0–41.0] to 38.0 [34.0–41.0] mmol/mol, *p* < 0.01. No statistically significant difference at 6 weeks for other parameters or at 18 weeks for any parameter.	The results favor treatment only for glycemic profile at 6 weeks.	High
Vrieze (2012) [49]	Randomized controlled trial	Male metabolic syndrome patients	FMT	6 weeks	Insulin sensitivity, glycemic and lipidic profile, weight	Median rate of glucose disappearance changed from 26.2 to 45.3 μmol/kg/min; *p* < 0.05.	The results mildly favor treatment for glycemic parameters.	High
Hartstra (2020) [50]	Randomized controlled trial	Metabolic syndrome patients, 50–70 y.o	FMT	4 weeks	Effect on brain dopamine transporter (DAT) and serotonin transporter (SERT) and insulin sensitivity	Increase in brain DAT. No effect on body weight and insulin sensitivity was demonstrated.	The results do not favor treatment.	High
**(C)**								
**Authors**	**Type of Studies**	**Number of Studies and Type of Study**		**Subject**	**End Point**	**Results**	**Conclusion**	**Strength of** **Evidence**
Zecheng (2023) [51]	Meta-analysis	10 randomized controlled trials (RCTs)		Obese and metabolic syndrome patients	Metabolic syndrome parameters, weight-related parameters	Significant reduction in cholesterol levels, blood pressure, and triglycerides; no statistically significant difference in other metabolic syndrome parameters or anthropometric parameters.	The results favor treatment only for some parameters of metabolic syndrome.	High
Pakhmer et al. [52]	Meta-analysis	11 studies: − 12 RCTs. − 4 controlled trials. − 1 open-label randomized controlled trial. − 1 clinical trial.		Obese and metabolic syndrome patients	Metabolic syndrome parameters, weight-related parameters	No significant long-term effects on metabolic parameters	The results do not favor treatment.	High

**Table 2:** (**A**) Diabetes and microbiota: randomized and non-randomized controlled trials. (**B**) Diabetes and microbiota: systematic reviews and meta-analyses. (**C**) Probiotics and diabetes: randomized and non-randomized clinical trials. (**D**) Probiotics and diabetes: systematic reviews and meta-analyses.

**(A)**									
**Authors**	**Type of Studies**	**Population** **Characteristics**	**Type of** **Laboratory Evaluation**	**End Point**		**Results**		**Conclusion**	**Strength of** **Evidence**
Que et al., 2021 [53]	Original Research	Patients with type 2 diabetes mellitus (T2DM) and healthy controls	16S rRNA gene sequencing, microbiota composition analysis	Gut bacterial characteristics of T2DM patients and potential therapeutic applications		Significant differences in gut microbiota composition between T2DM patients and healthy controls. Reduced diversity and altered relative abundances of specific bacterial taxa were observed.		Understanding gut bacterial characteristics in T2DM can inform potential therapeutic applications targeting the gut microbiota.	Medium
Pellegrini et al., 2017 [54]	Case–control study	19 patients with T1D compared with 16 healthy controls (CTRLs) subjects and 19 patients with celiac disease (CD) as gut inflammatory disease controls	The inflammatory status and microbiome composition were evaluated in biopsies of the duodenal mucosa	Evaluate the gut inflammatory profile and microbiota in patients with T1D compared with healthy control (CTRL) subjects and patients with celiac disease (CD) as gut inflammatory disease controls		An increased expression of CCL13, CCL19, CCL22, CCR2, COX2, IL4R, CD68, PTX3, TNFα, and VEGFA was observed in patients with T1D compared with CTRL subjects and patients with CD. Immunohistochemical analysis confirmed T1D-specific inflammatory status compared with healthy and CD control tissues, mainly characterized by the increase in the monocyte/macrophage lineage infiltration. The T1D duodenal mucosal microbiome results were different from the other groups, with an increase in Firmicutes and Firmicutes/Bacteroidetes ratio and a reduction in Proteobacteria and Bacteroidetes. The expression of genes specific for T1D inflammation was associated with the abundance of specific bacteria in the duodenum.		This study shows that duodenal mucosa in T1D presents disease-specific abnormalities in the inflammatory profile and microbiota	Medium
Wu et al., 2017 [55]	Clinical study	Individuals with treatment-naive type 2 diabetes	16S rRNA gene sequencing, metagenomics, blood biochemistry, glucose tolerance test	Impact of metformin on gut microbiota composition and metabolic markers		Metformin treatment significantly altered gut microbiota composition, increasing the abundance of beneficial bacteria like Akkermansia and reducing pathogenic bacteria. Metformin also improved glucose metabolism and insulin sensitivity.		Metformin’s therapeutic effects in type 2 diabetes may be partly mediated by its impact on gut microbiota. Understanding these mechanisms can help optimize diabetes treatment strategies.	Medium
Alkanani et al., 2015 [56]	Original Research	35 subjects with islet autoimmunity living in the U.S.	High-throughput sequencing of bacterial 16S rRNA genes	Correlation of intestinal microbiota alterations with susceptibility to type 1 diabetes (T1D)		Significant differences in gut microbiomes of seropositive subjects compared to autoantibody-free relatives, including different levels of Firmicutes and Bacteroidetes genera. No differences in biodiversity between seropositive and seronegative relatives.		Altered intestinal microbiota may be associated with disease susceptibility. Specific bacterial taxa differ in abundance between individuals with and without autoantibodies, suggesting a potential role in T1D progression.	Medium
David et al., 2014 [57]	Controlled clinical trial	six male and four female American volunteers between the ages of 21 and 33, BMI from 19 to 32 kg/m^2^	Each day, subjects logged their food intake and non-invasively sampled their gut microbiota. DNA was extracted from all fecal samples as previously described, sequenced using 16S rRNA- and ITS-specific primers, and analyzed with the Quantitative Insights Into Microbial Ecology (QIIME) software package and custom Python Scripts. SCFA analysis was performed by gas chromatography, and bile-acid analysis used enzymatic assays and mass spectrometry.	Whether dietary interventions in humans can alter gut microbial communities in a rapid, diet-specific manner		The animal-based diet had a greater impact on the gut microbiota than the plant-based diet: there were 22 clusters whose abundance significantly changed while on the animal-based diet, whereas only 3 clusters showed significant abundance changes while on the plant-based diet; the genus Prevotella, one of the leading sources of inter-individual gut microbiota variation and hypothesized to be sensitive to long-term fiber intake, was reduced in our vegetarian subject during consumption of the animal-based diet. We also observed a significant positive correlation between subjects’ fiber intake over the past year and baseline gut Prevotella levels.		Our findings that the human gut microbiome can rapidly switch between herbivorous and carnivorous functional profiles may reflect past selective pressures during human evolution. The microbiota changes on the animal-based diet could be linked to altered fecal bile acid profiles and the potential for human enteric disease.	High
Murri et al., 2013 [58]	Original Research	Children with type 1 diabetes mellitus (T1DM) and healthy controls	16S rRNA sequencing, microbiota composition analysis	Comparison of gut microbiota between children with T1DM and healthy controls		Children with T1DM had a different gut microbiota composition compared to healthy controls, with lower diversity and different relative abundances of specific bacterial taxa.		Gut microbiota dysbiosis is associated with T1DM in children. Modulating gut microbiota could be a potential strategy for managing T1DM.	Medium
**(B)**									
**Authors**	**Type of Studies**	**Number of Studies and Type of Study**	**Subject**	**End Point**		**Results**		**Conclusion**	**Strength of** **Evidence**
Bajinka et al., 2023 [59]	Review	Various populations with focus on gut microbiota and diabetes	Various laboratory evaluations related to gut microbiota and metabolic markers	Mechanisms and pathways of type 2 diabetes mellitus (T2DM)		Highlights the role of gut microbiota in the development and management of T2DM; discusses inconsistencies in findings		Emphasizes the need for more consistent and robust study designs to better understand the relationship between gut microbiota and diabetes and to develop effective treatments	Medium–high
Crudele et al., 2023 [60]	Narrative review	Studies include both animal models (mice) and human trials focusing on obesity, metabolic syndrome, and type 2 diabetes	Various trials involve gut microbiota profiling, glucose metabolism, lipid analysis, SCFA concentration analysis, and inflammatory markers	Evaluating the role of gut microbiota in the pathogenesis and potential therapeutic interventions for diabetes and metabolic diseases		Gut microbiota plays a significant role in obesity and diabetes; reduced butyrate-producing species are linked with insulin resistance. Certain probiotics like *Lactobacillus* show potential in improving insulin sensitivity and decreasing inflammation.		Dysbiosis in gut microbiota contributes to metabolic endotoxemia and chronic inflammation, which can drive the development of insulin resistance and T2D. Probiotic supplementation shows promise as a potential therapy to improve insulin resistance and inflammation in diabetic patients.	Medium
Ye et al., 2022 [61]	Review	Various studies on human and animal models	Metagenomics, functional studies, microbiota composition analysis	Role of gut microbiota in the pathogenesis and treatment of diabetes mellitus		Gut microbiota dysbiosis is linked to the development of type 1 and type 2 diabetes mellitus. Dysbiosis may cause gut leakiness and immune responses that damage pancreatic β cells or cause metabolic disorders.		Understanding gut microbiota’s role in diabetes pathogenesis can lead to new therapeutic strategies, including microbiological therapies, to improve diabetes management.	Medium–high
Mokhtari et al., 2021 [62]	Review	Children and adolescents with type 1 diabetes (T1D) and healthy controls	Metagenomics, 16S rRNA sequencing, functional analyses	Impact of T1D on the composition and functional potential of gut microbiome in children and adolescents		Significant alterations in gut microbiota diversity, taxonomic profiles, and functional potential in children with T1D compared to healthy controls. Dysbiosis is associated with increased intestinal permeability, altered immune responses, and chronic inflammation.		Gut microbiota dysbiosis plays a significant role in the pathogenesis of T1D. Understanding these changes can lead to the development of microbiome-based therapeutic strategies for T1D prevention and treatment.	Medium–high
Zhu and Goodarzi, 2020 [63]	Review	Various studies on human subjects	Metabolomics, gut microbiome analysis, bioinformatics	Linking gut microbiome metabolites with risk for type 2 diabetes		Metabolites produced by gut microbiota, such as short-chain fatty acids (SCFAs), bile acids, and branched-chain amino acids, are associated with insulin resistance and type 2 diabetes. Altered gut microbiota composition leads to increased production of harmful metabolites and decreased production of beneficial metabolites.		Gut microbiota-derived metabolites play a crucial role in the pathogenesis of type 2 diabetes. Targeting these metabolites through dietary interventions, probiotics, and other therapies may offer new strategies for diabetes prevention and treatment.	Medium–high
**(C)**									
**Authors**	**Type of Studies**	**Population** **Characteristics**	**Type of Laboratory Evaluation**	**Type of** **Intervention**	**Duration**	**End Point**	**Results**	**Conclusion**	**Strength of** **Evidence**
David et al., 2014 [57]	Controlled clinical trial	six male and four female American volunteers between the ages of 21 and 33, BMI from 19 to 32 kg/m^2^	Each day, subjects logged their food intake and non-invasively sampled their gut microbiota. DNA was extracted from all fecal samples as previously described, sequenced using 16S rRNA- and ITS-specific primers, and analyzed with the Quantitative Insights Into Microbial Ecology (QIIME) software package and custom Python Scripts. SCFA analysis was performed by gas chromatography, and bile-acid analysis used enzymatic assays and mass spectrometry.	Two diets: a ‘plant-based diet’, which was rich in grains, legumes, fruits and vegetables, and an ‘animal-based diet’, which was composed of meats, eggs and cheeses.	10 days	Whether dietary interventions in humans can alter gut microbial communities in a rapid, diet-specific manner	The animal-based diet had a greater impact on the gut microbiota than the plant-based diet: there were 22 clusters whose abundance significantly changed while on the animal-based diet, whereas only 3 clusters showed significant abundance changes while on the plant-based diet; the genus Prevotella, one of the leading sources of inter-individual gut microbiota variation and hypothesized to be sensitive to long-term fiber intake, was reduced in our vegetarian subject during consumption of the animal-based diet. We also observed a significant positive correlation between subjects’ fiber intake over the past year and baseline gut Prevotella levels.	Our findings that the human gut microbiome can rapidly switch between herbivorous and carnivorous functional profiles may reflect past selective pressures during human evolution. The microbiota changes on the animal-based diet could be linked to altered fecal bile acid profiles and the potential for human enteric disease.	High
**(D)**									
**Authors**	**Type of Studies**	**Number of Studies and Type of Study**	**Population Characteristics**	**Type of Laboratory Evaluation**	**End Point**		**Results**	**Conclusion**	**Strength of** **Evidence**
Wang et al., 2024 [64]	Meta-analysis	32 RCTs	1676 patients with DM2	HbA1c, insulin, HOMA-IR, FBG, BMI	Glycemic control (HbA1c, insulin, HOMA-IR, FBG, BMI)		Probiotics significantly reduced HbA1c (−0.33), insulin (−0.48), and HOMA-IR (−1.36). No effect on BMI.	Probiotics improve glycemic control (HbA1c, insulin, HOMA-IR) in T2DM, especially at 6–8 and 12–24 weeks.	High
Li et al., 2023 [65]	Meta-analysis	30 RCTs	1872 patients with DM2	HbA1c, insulin, HOMA-IR, FBG, BMI	Glycemic control (HbA1c, insulin, HOMA-IR, FBG, BMI)		Probiotics significantly reduced FBG, HbA1c, insulin, and HOMA-IR. Better results in BMI > 30 at baseline and probiotics with *Bifidobacterium*, while there were no differences with duration and dose of probiotics.	Probiotics improve glycemic control (HbA1c, insulin, HOMA-IR) in T2DM, especially in patients with obesity and probiotics including both *Lactobacillus* and *Bifidobacterium*.	High
Moravejolahkami et al., 2023 [66]	Meta-analysis	5 RCTs	356 patients with DM2	FGB, HbA1c, C-peptide, insulin therapy	Glycemic control (FBG, HbA1c) and doses of insulin therapies		Probiotics significantly reduced FBG, while there were no effects on HbA1c, C-peptide, or insulin doses.	Probiotics improve glycemic control in T2DM	High
Ayesha et al., 2023 [67]	Meta-analysis	22 RCTs	2218 patients with DM2	Fasting blood glucose (FBG), HbA1c, HOMA-IR	Glycemic control (FBG, HbA1c, HOMA-IR)		Probiotics significantly reduced HbA1c, FBG, and HOMA-IR compared to placebo.	Probiotics improve glycemic control in T2DM, particularly in long-term interventions (12+ weeks).	High
Zhang et al., 2022 [68]	Meta-analysis	33 CTRs	1927 patients with DM2	HbA1c, FBG, fasting insulin, HOMA-IR	Glycemic control (HbA1c, FBG, fasting insulin, HOMA-IR)		Probiotics significantly reduced HbA1c (−0.19%), FBG (−1.00 mmol/L), and HOMA-IR (−1.00), but not fasting insulin (−5.73 pmol/L).	Probiotics improved glycemic control in T2DM, but reductions in HbA1c were not clinically significant.	High
Naseri et al., 2022 [69]	Meta-analysis	46 RCTs	3067 patients with DM2 and prediabetes	FPG, fasting insulin, HbA1c, HOMA-IR, QUICKI	Glycemic control (FPG, HbA1c, fasting insulin, HOMA-IR)		Probiotics and synbiotics significantly reduced FPG (−11.18 mg/dL), HbA1c (−0.35%), insulin (−1.23 µIU/mL), and HOMA-IR (−0.87). No change in OGTT.	Probiotics and synbiotics improve glycemic control in individuals with prediabetes and T2DM.	High
Kocsis et al., 2020 [70]	Meta-analysis	32 RCTs	1676 patients with DM2	HbA1c, FPG, fasting insulin, CRP, blood pressure	Glycemic control, metabolic parameters (cholesterol, triglycerides, BP)		Probiotics significantly reduced HbA1c (−0.33%), FPG (−16.52 mg/dL), fasting insulin (−1.40 µIU/mL), CRP (−0.43 mg/dL), and blood pressure. HDL increased, but no effect on BMI.	Probiotics improve glycemic control and metabolic parameters in T2DM patients, especially HbA1c and FPG.	High

**Table 3:** (**A**) Prediabetes and microbiota: randomized and non-randomized controlled trials. (**B**) Prediabetes and microbiota: systematic reviews and meta-analyses. (**C**) Probiotics and prediabetes: randomized and non-randomized clinical trials. (**D**) Probiotics and prediabetes: systematic reviews and meta-analyses.

**(A)**									
**Authors**	**Type of Studies**	**Population** **Characteristics**	**Type of Laboratory Evaluation**	**Type of ** **Intervention** **(If Applicable)**	**Period of** **Intervention**	**End Point**	**Results**	**Conclusion**	**Strength of ** **Evidence**
Larsen et al., 2010 [71]	Observational Study	36 male adults with a broad range of age and body mass indices (BMIs), among which 18 subjects were diagnosed with DM2	The fecal bacterial composition was investigated by real-time quantitative PCR (qPCR) and in a subgroup of subjects (N = 20) by tag-encoded amplicon pyrosequencing of the V4 region of the 16S rRNA gene.	-	-	To assess the differences between the composition of the intestinal microbiota in humans with DM2 and non-diabetic persons as control	The proportions of phylum Firmicutes and class Clostridia were significantly reduced in the diabetic group compared to the control group. The ratios of Bacteroidetes to Firmicutes as well as the ratios of Bacteroides–Prevotella group to *C. coccoides*–E. rectale group correlated positively and significantly with plasma glucose concentration but not with BMI. Similarly, class Betaproteobacteria was highly enriched in diabetic compared to non-diabetic persons and positively correlated with plasma glucose.	DM2 in humans is associated with compositional changes in intestinal microbiota. The level of glucose tolerance should be considered when linking microbiota with metabolic diseases such as obesity and developing strategies to control metabolic diseases by modifying the gut microbiota.	Low
Qin J. et al., 2012 [2]	Observational Study	123 patients with metabolic syndrome (MetS) and 304 controls	DNA was extracted from a total of 1770 stool samples using the MagPure Stool DNA KF kit. DNA library construction based on DNA nanospheres (DNB) and shotgun metagenomic sequencing based on combined probe anchoring synthesis (CPAS) were performed on all samples	-	-	To determine if the gut microbiome plays a role in MetS development and progression	MetS patients possessed significantly lower gut microbiome diversity; 28 bacterial species were negatively correlated with waist circumstance, with *Alistipes onderdonkii* showing the strongest correlation, followed by *Bacteroides thetaiotaomicron*, *Clostridium asparagiforme*, *C. citroniae*, *C. scindens*, and *Roseburia intestinalis*. These species were also enriched in controls relative to MetS patients. Pathways involved in the biosynthesis of carbohydrates, fatty acids, and lipids were enriched in the MetS group, indicating that microbial functions related to fermentation may play a role in MetS. Microbiome changes in MetS patients may aggravate inflammation and contribute to MetS diseases by inhibiting the production of short-chain fatty acids (SCFAs).	Results indicate potential utility of beneficial gut microbiota as a potential therapeutic to alleviate MetS.	Low
Li Y. et al., 2015 [72]	Observational Study	203 and 308 men and women from the NHS II and the HPFS, excluding those with diabetes, cardiovascular disease, cancer, or implausible dietary data at baseline	Dietary phosphatidylcholine was estimated by a valid food frequency questionnaire, with approximately 130 food items administered every 2 or 4 years combined with the phosphatidylcholine contents from the U.S. Department of Agriculture database and from values published by Zeisel et al.	-	-	To study the association between dietary phosphatidylcholine and risk of type 2 diabetes (T2D).	Study associated dietary intakes of phosphatidylcholine with incident T2D risk in multiple prospective cohorts with a large sample size, high rates of long-term follow-up, and detailed and repeated assessments of diet and lifestyle	The findings lend support to dietary intervention strategies targeting dietary sources of gut microbiota metabolites in prevention of T2D.	Low
Tang WH et al., 2017 [73]	Observational Study	1216 stable patients with T2DM who underwent elective diagnostic coronary angiography	Quantification of fasting plasma TMAO concentrations was performed utilizing stable isotope dilution liquid chromatography with online tandem mass spectrometry (LC/MS/MS) on an AB Sciex API 5500 triple quadrupole mass spectrometer (SCIEX, Toronto, Canada).	-	-	To study the relation between fasting TMAO and two of its nutrient precursors, choline and betaine, and T2DM and glycemic control	TMAO and choline concentrations were higher in individuals with T2DM vs. healthy controls. Within T2DM patients, higher plasma TMAO was associated with a significant 3.0-fold increased 3-year major adverse cardiac event risk and a 3.6-fold increased 5-year mortality risk. Increased TMAO concentrations remained predictive of both major adverse cardiac events and mortality risks in T2DM patients.	Fasting plasma concentrations of the proatherogenic gut microbe-generated metabolite TMAO are higher in diabetic patients and portend higher major adverse cardiac events and mortality risks independent of traditional risk factors, renal function, and relationship to glycemic control.	Low
Roy s. et al., 2020 [74]	Observational Study	Three-hundred diabetes-free men and women (77%) aged 20–55 years (mean = 34 ± 10) were enrolled at baseline and re-examined at 2 years.	Plasma TMAO was measured using Ultra-. Chromatography–Mass Spectrometry. After an overnight fast, FPG was measured longitudinally; HbA1C and insulin were measured only at baseline. Insulin resistance was defined using HOMA-IR.	-	-	To investigate the role of TMAO as an early biomarker of longitudinal glucose increase or prevalent impaired glucose regulation	Multivariable relative risk regressions modeled prevalent prediabetes across TMAO tertiles. Mean values of 2-year longitudinal FPG ± SE across tertiles of TMAO were 86.6 ± 0.9, 86.7 ± 0.9, and 86.4 ± 0.9 (*p* = 0.98). Trends were null for FPG, HbA1c, and HOMA-IR, cross-sectionally. The prevalence ratios of prediabetes among participants in 2nd and 3rd TMAO tertiles (vs. the 1st) were 1.94 [95%CI 1.09–3.48] and 1.41 [95%CI: 0.76–2.61].	TMAO levels are associated with increased prevalence of prediabetes in a nonlinear fashion but not with insulin resistance or longitudinal FPG change.	Low
Allin KH et. al., 2018 [75]	Case–control Study	134 Danish adults with prediabetes, overweight, insulin resistance, dyslipidemia and low-grade inflammation and 134 age- and sex-matched individuals with normal glucose regulation	Biochemical analyses were performed on fasting blood samples. Fecal microbiota composition was profiled by sequencing the V4 region of the 16S rRNA gene on an Illumina MiSeq instrument.	-	-	To study if specific gut microbiota profiles are associated with prediabetes and a range of clinical biomarkers of poor metabolic health	Here, 5 bacterial genera and 36 operational taxonomic units (OTUs) were differentially abundant between individuals with prediabetes and those with normal glucose regulation. At the genus level, the abundance of Clostridium was decreased, whereas the abundances of Dorea, [Ruminococcus], Sutterella and Streptococcus were increased. The two OTUs that differed the most were a member of the order Clostridiales (OTU 146564) and *Akkermansia muciniphila*, which both displayed lower abundance among individuals with prediabetes. Fecal transfer from donors with prediabetes or screen-detected, drug-naive type 2 diabetes to germfree Swiss Webster or conventional C57BL/6 J mice did not induce impaired glucose regulation in recipient mice.	Data show that individuals with prediabetes have aberrant intestinal microbiota characterized by a decreased abundance of the genus Clostridium and the mucin-degrading bacterium *A. muciniphila*. Our findings are comparable to observations in overt chronic diseases characterized by low-grade inflammation	Low
Zhong H. et al., 2019 [76]	Observational Study	Fecal samples from treatment-naïve type 2 diabetic (TN-T2D, n = 77), prediabetic (Pre-DM, n = 80), and normal glucose-tolerant (NGT, n = 97) individuals	A combination of in-depth metagenomics and metaproteomics analyses of fecal samples	-	-	To study differences in the gut microbiome of T2D and prediabetic individuals compared to healthy individuals, without confounding factors such as antidiabetic medication, and identify gut microbial changes in disease development	The study observed distinct differences characterizing the gut microbiota of these three groups and validated several key features in an independent TN-T2D cohort. It also demonstrated that the content of several human antimicrobial peptides and pancreatic enzymes differed in fecal samples between three groups.	These findings suggest a complex, disease stage-dependent interplay between the gut microbiota and the host and point to the value of metaproteomics to gain further insight into interplays between the gut microbiota and the host.	Low
**(B)**									
**Authors**	**Type of Studies**	**Number of Studies and Type of Study**	**Subject**	**End Point**		**Results**	**Conclusion**		**Strength of** **Evidence**
Letchumanan G. et al., 2022 [77]	Systematic Review of Observational Studies	18 observational studies	5489 subjects	To summarize the existing evidence related to microbiota composition and diversity in individuals with prediabetes (preDM) and individuals newly diagnosed with T2DM (newDM) in comparison to individuals with normal glucose tolerance (nonDM)		Low gut microbial diversity was generally observed in preDM and newDM when compared to nonDM. Differences in gut microbiota composition between the disease groups and nonDM were inconsistent across the included studies. Four out of the eighteen studies found increased abundance of phylum Firmicutes along with decreased abundance of Bacteroidetes in newDM. At the genus/species levels, decreased abundance of *Faecalibacterium prausnitzii*, *Roseburia*, *Dialister*, *Flavonifractor*, *Alistipes*, *Haemophilus* and *Akkermansia muciniphila* and increased abundance of *Lactobacillus*, *Streptococcus*, *Escherichia*, *Veillonella* and *Collinsella* were observed in the disease groups in at least two studies. *Lactobacillus* was also found to positively correlate with fasting plasma glucose (FPG), HbA1c and/or homeostatic assessment of insulin resistance (HOMA-IR) in four studies.	There is a need for further investigations on the species/strain-specific role of endogenously present *Lactobacillus* in glucose regulation mechanism and T2DM disease progression. Differences in dietary intake caused significant variation in specific bacterial abundances. More studies are needed to establish more consistent associations between clinical biomarkers or dietary intake and specific gut bacterial composition in prediabetes and early T2DM.		High
Gurung M. et al., 2019 [78]	Narrative review	42 studies (preclinical studies or clinical trials) using treatments with probiotics	-	To summarize the potential role of different bacterial taxa affecting diabetes		The genera of *Bifidobacterium*, *Bacteroides*, *Faecalibacterium*, *Akkermansia* and *Roseburia* were negatively associated with T2D, while the genera of *Ruminococcus*, *Fusobacterium*, and *Blautia* were positively associated with T2D.	Despite multiple studies supporting the importance of gut microbiota in the pathophysiology of T2D, the field is in an early stage. Some microbial taxa and related molecular mechanisms may be involved in glucose metabolism related to T2D. However, the heterogeneity of T2D and redundancy of gut microbiota do not promise simple interpretations (e.g., low diversity) and easy solutions (such as fecal transplant from non-diabetic/non-obese donor).		Medium
Aw W. et al., 2018 [79]	Narrative review	-	-	To review the labyrinth encompassing the gut microbiota and gut microbiota-derived metabolites in type 1 diabetes and type 2 diabetes pathogenesis		The studies included in the present review emphasize that diabetes pathogenesis could be a result of specific pathogens, but metabolites produced by gut microbiota, such as bile acids, also play an important part. However so, the exact impacts of gut microbes and their metabolites on the incidence and pathogenesis of diabetes have yet to be clearly elucidated.	As diabetes is multifactorial and can progress to other related metabolic diseases, it is of utmost importance that the delicate interrelationships between gut microbiota and host metabolism are well understood in order to suggest appropriate lifestyle and nutritional interventions by engineering an optimal gut environment towards the prevention and maintenance/remission of diabetes.		Medium
**(C)**									
**Authors**	**Type of Studies**	**Population** **Characteristics**	**Type of Laboratory Evaluation**	**Type of** **Intervention**	**Duration**	**End Point**	**Results**	**Conclusion**	**Strength of** **Evidence**
Simon MC. et al., 2015 [80]	Prospective, double-blind, randomized trial	21 glucose-tolerant humans (11 lean: age 49 ± 7 years, BMI 23.6 ± 1.7 kg/m^2^; 10 obese: age 51 ± 7 years, BMI 35.5 ± 4.9 kg/m^2^)	Oral glucose tolerance and isoglycemic glucose infusion tests were used to assess incretin effect and GLP-1 and GLP-2 secretion, and euglycemic–hyperinsulinemic clamps with [6,6-(2)H2] glucose were used to measure peripheral insulin sensitivity and endogenous glucose production. Muscle and hepatic lipid contents were assessed by (1)H-magnetic resonance spectroscopy, and immune status, cytokines, and endotoxin were measured with specific assays.	Participants ingested 10(10) b.i.d. *L. reuteri* SD5865 or placebo over 4 weeks.	4 weeks	The study hypothesized that daily intake of *L. reuteri* increases insulin sensitivity by changing cytokine release and insulin secretion via modulation of the release of GLP-1 and -2.	In glucose-tolerant volunteers, daily administration of *L. reuteri* SD5865 increased glucose-stimulated GLP-1 and GLP-2 release by 76% (*p* < 0.01) and 43% (*p* < 0.01), respectively, compared with placebo, along with 49% higher insulin (*p* < 0.05) and 55% higher C-peptide secretion (*p* < 0.05). However, the intervention did not alter peripheral and hepatic insulin sensitivity, body mass, ectopic fat content, or circulating cytokines.	Enrichment of gut microbiota with *L. reuteri* increases insulin secretion, possibly due to augmented incretin release, but does not directly affect insulin sensitivity or body fat distribution. This suggests that oral ingestion of one specific strain may serve as a novel therapeutic approach to improve glucose-dependent insulin release.	High
M. Hariri et al., 2015 T2D [81]	Randomized, double-blind, placebo-controlled trial	Forty patients with type 2 diabetes mellitus aged 35–68 years were assigned to two groups.	Genomic DNA was extracted from EDTA anticoagulated whole blood and was quantified by a Nano Drop spectrophotometer. Than, the promoter methylation analysis was performed using methylation-specific digestion enzyme and real-time polymerase chain reaction (PCR).	Pts in the intervention group consumed 200 mL/day of probiotic soy milk containing *L. plantarum* A7, while those in the control group consumed 200 mL/d of conventional soy milk for 8 weeks.	8 weeks	To discover the effects of probiotic soy milk and soy milk on MLH1 and MSH2 promoter methylation, and oxidative stress among type 2 diabetic patients	Probiotic soy milk significantly decreased promoter methylation in proximal and distal MLH1 promoter region (*p* < 0.01 and *p* < 0.0001, respectively) compared with the baseline values, while plasma concentration of 8-hydroxy-2′-deoxyguanosine (8-OHdG) decreased significantly compared with soy milk (*p* < 0.05). In addition, a significant increase in superoxide dismutase (SOD) activity was observed in probiotic soy milk group compared with baseline value (*p* < 0.01). There were no significant changes from baseline in the promoter methylation of MSH2 within either group (*p* > 0.05).	The consumption of probiotic soy milk improved antioxidant status in type 2 diabetic patients and may decrease promoter methylation among these patients, indicating that probiotic soy milk is a promising agent for diabetes management.	High
M. Sanchez et al., 2014 [82]	Randomized, double-blind, placebo-controlled trial	153 obese men and women	Anthropometric parameters (body weight, height and waist circumference) and body composition measured by dual-energy X-ray absorptiometry. Biochemical analyses: plasma concentrations of glucose, insulin, leptin, lipids, lipoproteins and inflammatory indicators. Sequence-based microbiota analysis from fecal samples using PCR.	Each subject consumed 2 capsules/d of either a placebo or an LPR formulation (1.6 × 10^8^ colony-forming units of LPR/capsule with oligofructose and inulin). Each group was submitted to moderate energy restriction for the first 12 weeks followed by 12 weeks of weight maintenance.	24 weeks.	To investigate the impact of a *Lactobacillus rhamnosus* CGMCC1.3724 (LPR) supplementation on weight loss and maintenance in obese men and women over 24 weeks	After the first 12 weeks and after 24 weeks, mean weight loss was not significantly different between the LPR and placebo groups when all the subjects were considered. However, a significant treatment × sex interaction was observed. The mean weight loss in women in the LPR group was significantly higher than that in women in the placebo group (*p* = 0.02) after the first 12 weeks, whereas it was similar in men in the two groups (*p* = 0.53). Women in the LPR group continued to lose body weight and fat mass during the weight maintenance period, whereas opposite changes were observed in the placebo group. Changes in body weight and fat mass during the weight maintenance period were similar in men in both the groups. LPR-induced weight loss in women was associated not only with significant reductions in fat mass and circulating leptin concentrations but also with the relative abundance of bacteria of the Lachnospiraceae family in feces.	The study shows that the *Lactobacillus rhamnosus* CGMCC1.3724 formulation helps obese women to achieve sustainable weight loss.	High
C.J. Hulston et al., 2015 [83]	Randomized controlled trial	Seventeen healthy subjects were randomized to either a probiotic (n = 8) or a control (n = 9) group.	Anthropometric parameters: body weight, height and BMI. Standardized forms and digital kitchen scales to record weighed food intake on 3 d each week during the pre-experimental period (days 1–21). Insulin sensitivity was determined from plasma glucose and serum insulin concentrations during an OGTT.	The probiotic group consumed an LcS-fermented milk drink twice daily for 4 weeks; the control group received no supplementation. Subjects maintained their normal diet for the first 3 weeks of the study, after which they consumed a high-fat (65% of energy), high-energy (50% increase in energy intake) diet for 7 d.	4 weeks	To determine whether probiotic supplementation (*Lactobacillus casei* Shirota (LcS)) prevents diet-induced insulin resistance in human subjects	Body mass increased by 0.6 (SE 0.2) kg in the control group (*p* < 0.05) and by 0.3 (SE 0.2) kg in the probiotic group (*p* > 0.05). Fasting plasma glucose concentrations increased following 7 d of overeating (control group: 5.3 (SE 0.1) vs. 5.6 (SE 0.2) mmol/L before and after overfeeding, respectively, *p* < 0.05), whereas fasting serum insulin concentrations were maintained in both groups. Glucose AUC values increased by 10% (from 817 (SE 45) to 899 (SE 39) mmol/L per 120 min, (*p* < 0.05) and whole-body insulin sensitivity decreased by 27% (from 5.3 (SE 1.4) to 3.9 (SE 0.9), (*p* < 0.05) in the control group, whereas normal insulin sensitivity was maintained in the probiotic group (4.4 (SE 0.8) and 4.5 (SE 0.9) before and after overeating, respectively (*p* > 0.05)).	These results suggest that probiotic supplementation may be useful in the prevention of diet-induced metabolic diseases such as type 2 diabetes.	High
Y. Kadooka et al., 2010 [84]	Multicenter, double-blind, randomized, placebo-controlled intervention trial	87 overweight/obese subjects	Measurement of body weight and other body parameters as well as blood pressure, pulse rate, and common blood and urinary tests were performed at each time point in weeks. Abdominal computed tomography scans for the measurement of abdominal fat area were carried out at W0 and W12. Each subject made a daily record of taking the test FM, habitual diet and exercise. A detailed dietary record was also made and analyzed to determine the intake of energy, protein, carbohydrate, fat and calcium.	Subjects were randomly assigned to receive either fermented milk (FM) containing LG2055 (active FM; n = 43) or FM without LG2055 (control FM; n = 44), and were asked to consume 200 g/day of FM for 12 weeks.	12 weeks	To evaluate the effects of the probiotic *Lactobacillus gasseri* SBT2055 (LG2055) on abdominal adiposity, body weight and other body measures in adults with obese tendencies	In the active FM group, abdominal visceral and subcutaneous fat areas significantly (*p* < 0.01) decreased from baseline by an average of 4.6% (mean (confidence interval)) (−5.8 (−10.0, −1.7) cm^2^) and 3.3% (−7.4 (−11.6, −3.1) cm^2^), respectively. Body weight and other measures also decreased significantly (*p* < 0.001) as follows: body weight, 1.4% (−1.1 (−1.5, −0.7) kg); BMI, 1.5% (−0.4 (−0.5, −0.2) kg/m^2^); waist, 1.8% (−1.7 (−2.1, −1.4) cm); hip, 1.5% (−1.5 (−1.8, −1.1) cm). In the control group, by contrast, none of these parameters decreased significantly. High-molecular weight adiponectin in serum increased significantly (*p* < 0.01) in the active and control groups by 12.7% (0.17 (0.07, 0.26) microg/mL) and 13.6% (0.23 (0.07, 0.38) microg/mL), respectively.	The probiotic LG2055 showed lowering effects on abdominal adiposity, body weight and other measures, suggesting its beneficial influence on metabolic disorders.	High
R. Mobini et al., 2016 [85]	Randomized, double-blind, placebo-controlled trial	46 people with type 2 diabetes	Biochemical analyses: glycated hemoglobin (HbA1c), serum bile acids and insulin sensitivity (assessed by glucose clamp). Other variables evaluated: liver fat content, body composition, body fat distribution (using MRI and MRS) and fecal microbiota composition (by 16S rRNA-based Illumina MiSeq sequencing). Questionnaires and diaries were used to determine total caloric intake.	46 people with type 2 diabetes to placebo or a low (10^8^ CFU/d) or high dose (10^10^ CFU/d) of *L. reuteri* DSM 17938 for 12 weeks	12 weeks.	To investigate the metabolic effects of 12-week oral supplementation with *Lactobacillus reuteri* DSM 17938 in patients with type 2 diabetes on insulin therapy	Supplementation with *L. reuteri* DSM 17938 for 12 weeks did not affect HbA1c, liver steatosis, adiposity or microbiota composition. Participants who received the highest dose of *L. reuteri* exhibited increases in insulin sensitivity index (ISI) and serum levels of the secondary bile acid deoxycholic acid (DCA) compared with baseline, but these differences were not significant in the between-group analyses. Post hoc analysis showed that participants who responded with increased ISI after *L. reuteri* supplementation had higher microbial diversity at baseline and increased serum levels of DCA after supplementation. In addition, increases in DCA levels correlated with improvement in insulin sensitivity in the probiotic recipients.	Intake of *L. reuteri* DSM 17938 for 12 weeks did not affect HbA1c in people with type 2 diabetes on insulin therapy; however, *L. reuteri* improved insulin sensitivity in a subset of participants and we propose that high diversity of the gut microbiota at baseline may be important.	High
A.S. Andreasen et al., 2010 [86]	Randomized, double-blind, placebo-controlled trial	Forty-five males with type 2 diabetes, impaired or normal glucose tolerance	OGTT. Plasma levels of TNF-α, IL-6 and IL-1 receptor antagonist (IL-1ra), C-reactive protein. Detection of *Lactobacillus acidophilus* in stool samples.	Treatment course with either *L. acidophilus* NCFM, one capsule/d (1 g; about 10^10^ colony-forming units), or placebo	4 weeks	To evaluate the effects of oral supplementation with the probiotic bacterium *Lactobacillus acidophilus* NCFM on insulin sensitivity and the inflammatory response	Insulin sensitivity was preserved among volunteers in the *L. acidophilus* NCFM group, whereas it decreased in the placebo group. Both baseline inflammatory markers and the systemic inflammatory response were, however, unaffected by the intervention	Intake of *L. acidophilus* NCFM for 4 weeks preserved insulin sensitivity compared with placebo, but did not affect the systemic inflammatory response.	High
L.J. Bernini et al., 2016 [42]	Randomized controlled trial	Fifty-one patients with MetS were selected and divided into a control group (n = 25) and a probiotic group (n = 26).	Anthropometric measurements: waist circumference (WC), body weight, and height and BMI. Biochemical analyses: glucose, total cholesterol, HDL-C, LDL-C), TGs. Insulin, TNF-α, IL-6.	The probiotic group consumed 80 mL of fermented milk with probiotics (with 2.72 × 10^10^ colony-forming units of *B. lactis* HN019) daily over the course of 45 d.	45 days	To evaluate the effect of consumption of milk containing the probiotic *B. lactis* HN019 on the classical parameters of MetS and other related cardiovascular risk factors	Daily ingestion of 80 mL fermented milk with 2.72 × 10^10^ colony-forming units of *B. lactis* HN019 showed significant reduction in body mass index (*p* = 0.017), total cholesterol (*p* = 0.009), and low-density lipoprotein (*p* = 0.008) compared with baseline and control group values. Furthermore, a significant decrease in tumor necrosis factor-α (*p* = 0.033) and interleukin-6 (*p* = 0.044) pro-inflammatory cytokines was observed.	These data showed potential effects of *B. lactis* HN019 in reducing obesity, blood lipids, and some inflammatory markers, which may reduce cardiovascular risk in patients with MetS.	High
**(D)**									
**Authors**	**Type of Studies**	**Number of Studies and Type of Study**	**Subjects**	**End Point**		**Results**	**Conclusion**		**Strength of** **Evidence**
Barengolts E. et al., 2016 [87]	Review of randomized controlled trials	Randomized controlled trials	-	To review the data from randomized controlled trials (RCTs) for the roles of microbiota and pre-, pro-, and synbiotics in metabolic conditions (obesity, prediabetes, and diabetes mellitus type 2 [DM2])		Microbiota could increase harvesting of energy from food and cause subclinical inflammation seen in metabolic disorders. Diet-related interventions including prebiotics, probiotics, and synbiotics may benefit metabolic conditions. Results of RCTs of prebiotics suggested a neutral effect on body weight, decreased fasting and postprandial glucose, and improved insulin sensitivity and lipid profile. Some inflammation markers were reduced, sometimes substantially (20–30%). RCTs for probiotics demonstrated significant but small effects on body weight (<3%) and metabolic parameters. The effect was seen mostly with fermented milk or yogurt compared to capsule form, consumption for at least 8 weeks, and use of multiple rather than a single bacterial strain. Changes in microbiota were seen at times with both pre- and probiotics. Pickled and fermented foods, particularly vegetables and beans, could serve as a dietary source of pre-, pro-, and synbiotics. These foods showed possible benefits for morbidity and mortality in prospective cohort studies.	Pre-, pro-, and synbiotics could prove useful, but further research is needed to clarify their clinical relevance for the prevention and management of metabolic disease.		High
Hampe, C.S. et al., 2017 [88]	Narrative review	-	-	To discuss the mechanisms employed by specific probiotic strains of *Lactobacillus* and *Bifidobacterium genuses*, which showed efficacy in the treatment of obesity and T2D		Some probiotic strains employ recurring beneficial effects, including the production of antimicrobial lactic acid, while other strains display highly unique features, such as hydrolysis of tannins.	A major obstacle in the evaluation of probiotic strains lays in the great number of strains, differences in detection methodology and measured outcome parameters. The understanding of further research should be directed towards the development of standardized evaluation methods to facilitate the comparison of different studies.		Medium
Wang X. et al., 2021 [89].	Systematic review of randomized controlled trials	8 RCTs	391 subjects	To review the data from randomized controlled trials (RCTs) and identify evidence for microbiota’s role and the use of probiotics, prebiotics, or synbiotics in prediabetes		The gut microbiota influences host metabolic disorders via the modulation of metabolites, including short-chain fatty acids (SCFAs), the endotoxin lipopolysaccharide (LPS), bile acids (BAs) and trimethylamine N-oxide (TMAO), as well as mediating the interaction between the gastrointestinal system and other organs. Due to the limited sources of studies, there are inconsistent outcomes between included studies. Probiotics can decrease glycated hemoglobin (HbA1c) and have the potential to improve post-load glucose levels. The supplementation of probiotics can suppress the rise in blood cholesterol, but the improvement cannot be verified. Prebiotics are failed to show an evident improvement in glycemic control, but their use caused changes in the composition of gut microbiota. A combination of probiotics and prebiotics in synbiotic supplementation is more effective than probiotics alone in glycemic control.	Using probiotics, prebiotics or synbiotics for the treatment of prediabetes, the benefits of modulating the abundance of gut microbiota were partially demonstrated. However, there is insufficient evidence to show significant benefits on glucose metabolism, lipid metabolism and body composition.		High
Li Y. et al., 2022 [90]	Meta-analysis and systematic review	7 randomized controlled trials	460 patients	To examine the effects of probiotics on eight factors in the prediabetic population by meta-analysis, namely, fasting blood glucose (FBG), HbA1c, homeostatic model assessment of insulin resistance (HOMA-IR), quantitative insulin sensitivity check index (QUICKI), total cholesterol (TC), triglyceride (TG), high-density lipoprotein cholesterol (HDL-C) and low-density lipoprotein cholesterol (LDL-C), and the mechanisms of action are summarized from the existing studies.		Probiotics were able to significantly decrease the levels of HbA1c (WMD, −0.07; 95% CI −0.11, −0.03; *p* = 0.001), QUICKI (WMD, 0.01; 95% CI 0.00, 0.02; *p* = 0.04), TC (SMD, −0.28; 95% CI −0.53, −0.22; *p* = 0.03), TG (SMD, −0.26; 95% CI −0.52, −0.01; *p* = 0.04), and LDL-C (WMD, −8.94; 95% CI −14.91, −2.97; *p* = 0.003) compared to levels in the placebo group. The effects on FBG (WMD, −0.53; 95% CI −2.31, 1.25; *p* = 0.56), HOMA-IR (WMD, −0.21; 95% CI −0.45, 0.04; *p* = 0.10), and HDL-C (WMD, 2.05; 95% CI −0.28, 4.38; *p* = 0.08) were not different from those of the placebo group.	Probiotics may fulfill an important role in the regulation of HbA1c, QUICKI, TC, TG and LDL-C in patients with prediabetes. In addition, based on existing studies, we concluded that probiotics may regulate blood glucose homeostasis in a variety of ways.		High
Zeighamy Alamdary S. et al., 2022 [91]	Systematic review of randomized controlled trials	15 randomized controlled trials	1116 subjects	To compile the results of clinical trials investigating the effects of pro-/pre-/synbiotics on prediabetic subjects from 2010 to 2020		Positive and significant effects of probiotics in the reduction in hyperglycemia, insulin concentration levels, lipid profile, and BMI (body mass index). Administration of probiotics may provide beneficial and healthful effects in the clinical management of patients with prediabetes and metabolic syndrome.	Different probiotic compositions have shown beneficial and noticeable effects on glucose homeostasis, lipid profiles, BMI, and inflammatory markers in subjects with prediabetes and metabolic syndrome and healthy individuals and could be advantageous in recomposing the gut microbiota back into the normal state during the prediabetic state.		High
Zhang Q. et al., 2015 [92]	Meta-analysis of randomized controlled trials	7 randomized controlled trials	438 subjects	To investigate the effects of probiotics on glucose metabolism in patients with type 2 diabetes mellitus		Probiotic consumption significantly changed fasting plasma glucose (FPG) by −15.92 mg/dL and glycosylated hemoglobin (HbA1c) by −0.54% compared with control groups. Meta-analysis of trials with multiple species of probiotics found a significant reduction in FPG. The duration of intervention for ≥8 weeks resulted in a significant reduction in FPG. Subgroup analysis of trials with species of probiotics did not result in a significant meta-analysis effect. Furthermore, the duration of intervention < 8 weeks did not result in a significant reduction in FPG. The results also showed that probiotic therapy significantly decreased homeostasis model assessment of insulin resistance (HOMA-IR) and insulin concentration.	This meta-analysis suggests that consuming probiotics may improve glucose metabolism by a modest degree, with a potentially greater effect when the duration of intervention is ≥8 weeks, or multiple species of probiotics are consumed.		High

**Table 4:** (**A**) Obesity and microbiota: randomized and non-randomized controlled trials. (**B**) Probiotics and obesity: randomized and non-randomized clinical trials. (**C**) Probiotics and obesity: systematic reviews and meta-analyses.

**(A)**									
**Authors**	**Type of Studies**	**Population** **Characteristics**	**Type of Laboratory Evaluation**	**Type of** **Intervention** **(If Applicable)**	**Period of** **Intervention**	**End Point**	**Results**	**Conclusion**	**Strength of** **Evidence**
Alemán JO et al., 2018 [93]	Interventional study	Obese postmenopausal women	VLCD, 16S rRNA sequencing, metabolomics, transcriptomics	Diet with 800 kcal/day (time constrained by the goal of losing 10% of body weight)	46.2 ± 15.3 days	Weight loss, microbiota composition, metabolic changes	VLCD-induced weight loss led to changes in gut microbiota composition and associated metabolic benefits	VLCD dietary intervention in obese women changed the composition of several fecal microbial populations while preserving the core fecal microbiome. Changes in individual microbial taxa and their functions correlated with variations in plasma metabolomes, fecal bile acid composition, and adipose tissue transcriptome.	Medium
**(B)**									
**Authors**	**Type of Studies**	**Population** **Characteristics**	**Type of** **Intervention**	**Duration**	**End Point**	**Type of Laboratory Evaluation**	**Results**	**Conclusion**	**Strength of** **Evidence**
Larsen N. et al., 2013 [94]	Randomized control trials	Obese adolescents, 50 subjects, ages 12–15	Intake of *Lactobacillus salivarius* Ls-33 or placebo	12 weeks	Impact on fecal microbiota	Real-time quantitative PCR, gas chromatography for short-chain fatty acids	Significant increase in ratios of *Bacteroidetes*, *Prevotella*, and *Porphyromonas*, a group to Firmicutes. No significant change in cell numbers of fecal bacteria and short-chain fatty acids.	*Lactobacillus salivarius* Ls-33 might modify fecal microbiota in a way not related to metabolic syndrome.	High
Sharafedtinov KK et al., 2013 [95]	Randomized double-blind placebo-controlled pilot study	Obese hypertensive patients, 40 subjects, ages 30–69	Hypocaloric diet supplemented with probiotic cheese (*Lactobacillus casei* group) or control cheese	3 weeks	Body mass index (BMI) and blood pressure (BP)	Molecular methods, gas chromatography for polyamines, standard lab methods for blood and urine analysis	Significant reduction in BMI and BP in the probiotic cheese group. Higher intestinal Lactobacilli associated with higher BMI and urinary putrescine content.	Hypocaloric diet supplemented with probiotic cheese helps reduce BMI and BP values, recognized symptoms of metabolic syndrome.	High
Parnell J.A. et al., 2009 [96]	Randomized, double-blind, placebo-controlled trial	48 overweight and obese adults, BMI > 25, ages 20–70	21 g of oligofructose per day vs. placebo for 12 weeks	12 weeks	Body weight, satiety hormone concentrations, glucose regulation	Dual-energy X-ray absorptiometry, meal tolerance tests, plasma analysis for glucose, insulin, ghrelin, GLP-1, PYY	Reduction in body weight by 1.03 ± 0.43 kg in oligofructose group, increase of 0.45 ± 0.31 kg in control group. Lower ghrelin and higher PYY levels. Improved glucose regulation.	Oligofructose supplementation promotes weight loss and improves glucose regulation independent of other lifestyle changes.	High
Sanchez M. et al., 2014 [82]	Randomized, double-blind, placebo-controlled trial	125 obese men and women, ages 18–55, BMI ~29–41 kg/m²	Two capsules per day of *Lactobacillus casei* CGMC1.3724 or placebo, combined with reduced energy consumption	24 weeks	Body weight and plasma markers	Dual-energy X-ray absorptiometry, biochemical assays for plasma markers, 16S ribosomal RNA gene sequencing for microbiota	Significant weight loss in women taking LPR compared to placebo, but no significant difference in men. Women continued to lose weight during maintenance phase. Reductions in fat mass and leptin concentration in women.	*Lactobacillus casei* CGMC1.3724 formulation supports sustainable weight loss in obese women.	High
Safavi M et al., 2013 [97]	Randomized triple-masked controlled trial	70 overweight and obese children and adolescents, ages 6–18, BMI ≥ 85th percentile	Synbiotic supplementation (probiotics and prebiotics) or placebo for 8 weeks	8 weeks	BMI Z-score, waist circumference, waist-to-hip ratio, serum triglycerides, total cholesterol, LDL cholesterol	Biochemical assays for serum markers, stool examination for bacterial count	Significant reduction in BMI Z-score, waist circumference, waist-to-hip ratio, serum triglycerides, total cholesterol, and LDL cholesterol in synbiotic group.	Synbiotic supplementation helps control excess weight and improve cardiometabolic risk factors in children and adolescents.	High
Jung SP 2013 [98].	Randomized, double-blind, placebo-controlled clinical trial	62 overweight and obese adults, ages 19–60, BMI ≥ 23 kg/m², fasting blood sugar ≥ 100 mg/dL	Daily supplementation of 1010 CFU *Lactobacillus gasseri* BNR17 or placebo for 12 weeks	12 weeks	Body weight, body fat, waist and hip circumference, biochemical parameters	Bioelectrical impedance analysis, computed tomography, blood test for metabolic markers	Slight reduction in body weight in BNR17 group. Significant decrease in waist and hip circumference compared to placebo. No significant changes in visceral adipose tissue.	*Lactobacillus gasseri* BNR17 may reduce weight and waist and hip circumference without dietary changes.	High
Zarrati M. 2014 [99]	Randomized controlled trial	Overweight and obese individuals, ages 18–50, BMI 25–40 kg/m²	Probiotic yogurt vs. conventional yogurt, with or without weight-loss diet	8 weeks	Fat distribution, gene expression of pro-inflammatory factors in peripheral blood mononuclear cells	Anthropometric measurements, gene expression analysis, blood tests	Probiotic yogurt significantly reduced body weight, BMI, waist circumference, and fat mass. Reduced expression of TNF-α and IL-6 genes in the probiotic group compared to control.	Probiotic yogurt consumption, with or without a weight-loss diet, improves body composition and reduces pro-inflammatory gene expression in overweight and obese individuals.	High
Ipar N. et al., 2015 [100]	Randomized controlled trial	Obese children, ages 7–18	Synbiotic supplementation vs. placebo	12 weeks	Anthropometric measurements, lipid profile, oxidative stress markers	Anthropometric measurements, blood tests for lipid profile and oxidative stress markers	Significant improvements in BMI, waist circumference, and lipid profile in the synbiotic group. Reduction in oxidative stress markers compared to placebo.	Synbiotic supplementation improves anthropometric measures and lipid profile and reduces oxidative stress in obese children.	High
Doria E. et al., 2013 [101]	Randomized, double-blinded, placebo-controlled trial	40 slightly overweight women aged 30 to 54	Hypocaloric diet supplemented with phyto-supplement (Re-Code®) containing phloridzin, isoflavones, and probiotics	90 days (with measurements at T30, T60, T90)	Body weight, fat mass, waist, thigh, and buttock circumference	Anthropometric measurements (body weight, height, BMI, circumferences), ultrasound skinfold thickness, statistical analysis	Significant reduction in body weight, fat mass, and waist, thigh, and buttock circumference in the treatment group compared to placebo	Phyto-supplementation, combined with a mild hypocaloric diet and moderate physical activity, is effective in reducing body weight and fat mass in overweight women	High
**(C)**									
**Authors**	**Type of Studies**	**Number of Studies and Type of Study**	**Subject**		**End Point**	**Results**	**Conclusion**		**Strength of** **Evidence**
Geng J. et al., 2022 [102]	Review	Literature review, data synthesis	Obese individuals, various populations		Role of gut microbiota in obesity and related diseases	Gut microbiota composition and metabolites play a critical role in obesity and related diseases. Potential therapeutic treatments include probiotics, prebiotics, dietary interventions, and FMT.	There is overwhelming evidence that the composition of the gut microbiota and metabolites impact the progression of obesity and obesity-related diseases.		Medium
Gomes et al., 2018 [103]	Review	22 studies (literature review, data synthesis)	Obese individuals		Role of gut microbiota in obesity and metabolism	Gut microbiota dysbiosis in obese individuals is linked to increased Firmicutes, Clostridium, and several other species, contributing to inflammation and altered satiety signaling.	Obesity was characterized by the presence of intestinal dysbiosis. The resulting dysbiosis could change the functioning of the intestinal barrier and the GALT. Intestinal dysbiosis could alter the production of gastrointestinal peptides related to satiety.		Medium
Crovesy L. et al., 2017 [104]	Systematic review	14 studies (observational studies and clinical trials)	Obese and lean adults		Gut microbiota composition differences between obese and lean individuals	Obese individuals have higher Firmicutes/Bacteroidetes ratio, increased *Firmicutes*, *Fusobacteria*, *Proteobacteria*, and *Lactobacillus*, and decreased *Verrucomicrobia*.	Probiotics have the potential to help in weight loss and fat mass loss in overweight subjects. The probiotics that aid weight loss include *Lactobacillus gasseri*, *L. casei*, *L. rhamnosus*, *L. acidophilus*, and *L. plantarum*.		High
Borgeraas et al., 2017 [105]	Systematic review and meta-analysis	15 randomized controlled trials (RCTs)	Overweight and obese individuals		Effect of probiotics on weight, BMI, fat mass, and body fat percentage	Reduction in body weight (−0.60 kg), BMI (−0.27 kg/m²), and body fat percentage (−0.60%). However, the effect on fat mass was not always significant.	Probiotic supplementation may slightly reduce body weight, BMI, and body fat percentage, offering a potential approach for obesity management.		Moderate for fat mass and fat percentage due to smaller study sample sizes and variability in probiotic strains used.
Cao N. et al., 2024 [106]	Meta-analysis or randomized controlled trials (RCT)	11 randomized controlled trials (RCTs)	Overweight or obese women treated with probiotics		Weight loss, glucose metabolism (insulin, fasting blood glucose), lipid metabolism	Probiotics significantly reduced waist circumference (WC), insulin levels, and LDL cholesterol, but had no significant effect on weight, BMI, or fat mass.	Probiotics can effectively reduce some metabolic parameters, especially WC and LDL-C, in overweight and obese women, but have limited effect on weight loss and BMI.		Moderate: Results varied by duration of intervention and inclusion of dietary/exercise factors, but consistent reductions in LDL-C and WC were noted.
Musazadeh V et al., 2022 [107]	Meta-analyses (umbrella review)	29 meta-analyses	Effects of probiotic supplementation on obesity		Evaluate the effect of probiotics on BMI, body weight, and waist circumference (WC)	Probiotics significantly reduced BMI (ES = −0.21), body weight (ES = −0.38), and waist circumference (ES = −0.60).	Probiotics can be an effective intervention for managing obesity by reducing BMI, weight, and waist circumference.		Moderate in 83% of studies; low or very low in 17% of studies.

**Table 5:** (**A**) Hyperhomocysteinemia and microbiota: systematic reviews and meta-analysis. (**B**) Probiotics and hyperhomocysteinemia: randomized and non-randomized clinical trials.

**(A)**									
**Authors**	**Type of Studies**	**Number of Studies and Type of Study**		**Subject**	**End Point**		**Results**	**Conclusion**	**Strength of** **Evidence**
Kaye et al. (2020) [108].	Systematic review	5 studies: 3 RCTs, 2 meta-analyses		75,541	Daily supplementation with 0.5–5.0 mg of folic acid typically lowers plasma Hcy levels by approximately 25%		Hyperhomocysteinemia is a known risk factor for coronary artery disease. In this regard, elevated levels of Hcy have been found in the majority of patients with vascular disease.	Folic acid supplementation should be recommended to any patient who has an elevated Hcy level	High
**(B)**									
**Authors**	**Type of Studies**	**Population** **Characteristics**	**Type of Laboratory Evaluation**	**Type of** **Intervention**	**Duration**	**End Point**	**Results**	**Conclusion**	**Strength of** **Evidence**
Strozzi et al. (2008) [109]	Pilot study controlled clinical trial	23 healthy volunteers	Strain effectiveness was evaluated by determination of the folate concentration in feces evacuated within 48 h before and after administration of the probiotics.	Volunteers were randomly assigned to 1 of 3 groups for treatment with a specific probiotic strain (5 × 10^9^ colony-forming units/d), *Bifidobacterium adolescentis* DSM 18350, *B. adolescentis* DSM 18352, and *Bifidobacterium pseudocatenulatum* DSM 18353, to produce folates in the human intestine.	2 weeks	If the probiotic treatments would cause a significant increase in folic acid concentration in human feces in all treated groups	Ingestion of these probiotic strains resulted in a significant increase in folic acid concentration in human feces in all treated groups	There was an increase in Faecalibaculum and Dubosiella phyla. The demonstrated ability of the probiotic microorganisms *B. adolescentis* DSM 18350, *B. adolescentis* DSM 18352, and *B. pseudocatenulatum* DSM 18353 to synthesize and secrete folates in the human intestinal environment may provide a complementary endogenous source of such molecules.	Medium
Majewska et al. (2020) [110]	Randomized double-blind placebo-controlled trial	50 obese women (aged 45–70 years)	Blood tests	Subjects were randomly assigned to take either a multispecies probiotic supplement (n = 25) or placebo (n = 25) for 12 weeks	12-week supplementation with a multispecies probiotic	The purpose was to assess if supplementation with probiotics can potentially be a natural therapeutic method for metabolic disorders.	At the end of the study, a significant decrease in Hcy, tumor necrosis factor α (TNF-α), total cholesterol (TC), low-density lipoprotein cholesterol (LDL) and triglyceride (TG) levels were observed in the probiotic group.	These multidirectional effects can potentially reduce cardiometabolic risks.	High

**Table 6:** (**A**) Dyslipidemia and microbiota: randomized and non-randomized controlled trials. (**B**) Dyslipidemia and microbiota: systematic reviews and meta-analyses. (**C**) Probiotics and dyslipidemia: randomized and non-randomized clinical trials. (**D**) Probiotics and dyslipidemia: systematic reviews and meta-analyses.

**(A)**									
**Authors**	**Type of Studies**	**Population** **Characteristics**	**Type of Laboratory Evaluation**	**Type of ** **Intervention**	**Period of** **Intervention**	**End Point**	**Results**	**Conclusion**	**Strength of ** **Evidence**
Cotillard et al., 2013 [111]	Randomized clinical trial	38 obese and 11 overweight people, including 8 men and 41 women, without chronic diseases	At 0, 6 and 12 weeks, blood (total cholesterol, HDL, triglycerides, insulin, glucose, and inflammatory markers) and fecal samples were collected and anthropometric measurements were performed (body composition).	Low-calorie high-protein diet for 6 weeks, followed by maintenance diet for another 6 weeks	12 weeks	Investigating temporal relationships between food intake, gut microbiota, and metabolic and inflammatory phenotypes, to assess possible association between microbiota and fat metabolism	A 35% reduction in energy intake after the first 6 weeks was associated with a reduction in fat mass and adipocyte diameter and improvements in insulin sensitivity and markers of metabolism and inflammation. Quantitative metagenomic analysis of the gut microbiome revealed the existence of a high proportion of individuals (23–40%) with low microbial richness, who had dyslipidemia associated with adiposity, increased insulin resistance, and low-grade inflammation.	The concomitant improvement in gut microbiome gene richness and bioclinical variables by dietary intervention suggests the possibility of moving from risk identification to risk reduction, on the assumption that less-rich microbiota are also less healthy	High
Koren et al., 2011 [112]	Randomized cross-sectional study	15 patients aged 45 to 47 years with atherosclerosis and as many healthy controls matched for age and sex	454 pyrosequencing of 16S rRNA genes was used to examine the bacterial diversity of atherosclerotic plaques, oral samples from swabs, and fecal samples from stool collection.	These studies were based on screening examinations of randomly selected population cohorts.	/	To study the association between microbiota composition and pathophysiological conditions associated with dyslipidemia and ectopic fat deposition, such as atherosclerosis and hepatic steatosis	Shared OTUs were observed among all three sites (oral, gut, and atherosclerotic plaques), consistent with the possibility that the oral and gastrointestinal microbiota are involved in the inflammatory processes responsible for atherosclerosis and that the atherosclerotic plaque microbiota may derive from the oral cavity and/or the gut. In addition, specific components of the oral/intestinal microbiota correlate with markers of disease: *Streptococcus* correlates with HDL, *Fusobacterium* correlates with LDL and total cholesterol, members of the families *Erysipelotrichaceae* and *Lachnospiraceae* in the gut correlate with LDL and total cholesterol.	Bacteria in the oral cavity and perhaps the gut may be correlated with disease markers of atherosclerosis.	High
Zhou et al., 2023 [113]	Two-sample Mendelian randomization study	Individuals of European descent	//	//	//	Causal association between gut microbiota and dyslipidemia	The families *Lachnospiraceae* and *Lactobacillaceae* are of notable importance and should be recognized as crucial microbiota in ameliorating dyslipidemia. The *Bacillota* phylum emerges as the most influential regulator of body lipid levels.	These findings demonstrate a causal link between gut microbiota and dyslipidemia within humans. The families *Lachnospiraceae* and *Lactobacillaceae* assume a noteworthy role in ameliorating lipid metabolism abnormalities.	High
**(B)**									
**Authors**	**Type of Studies**	**Number of Studies and Type of Study**	**Subject**	**End Point**		**Results**		**Conclusion**	**Strength of** **Evidence**
Flaig et al., 2023 [114]	Narrative review	//	//	Association between gut microbiota, dysbiosis, and altered lipid metabolism; effects of key gut microbial metabolites on the development and progression of dyslipidemia; how diet impacts changes in the gut microbiota and the resulting influences on lipid metabolism		Improved dietary intake through the MD; statin therapy exerts its positive effects by increasing SCFA-producing bacteria like *Lactobacillus*, *Eubacterium*, *Faecalibacterium*, *Bifidobacterium* and *Akkermansia*, which are key in maintaining gut barrier integrity. Prebiotics, probiotics, synbiotics, FMT, and next-generation probiotics provide a simple, effective treatment modality for dyslipidemia by enriching the gastrointestinal tract of affected individuals with beneficial microbial species.		Substantial evidence supports the involvement of gut microbiota and states of dysbiosis in the development and progression of metabolic diseases, such as dyslipidemia.	Medium
**(C)**									
**Authors**	**Type of Studies**	**Population** **Characteristics**	**Type of Laboratory Evaluation**	**Type of** **Intervention**	**Duration**	**End Point**	**Results**	**Conclusion**	**Strength of** **evidence**
Tian Y et al., 2024 [115]	Randomized, double-blind, placebo-controlled clinical trial	33 patients with hyperlipidemia, divided into a probiotic group (n = 18) and a control group (n = 15)	16S rRNA gene pyrosequencing to examine bacterial diversity, from serum and stool samples	The probiotic group was given probiotics (2 g/day) and atorvastatin, 20 mg/day, while the control group was given placebo (2 g/day) and atorvastatin 20 mg/day.	3 months	To evaluate the role of probiotics (*Lactobacillus casei* Zhang, Bifidobactetium animalis subsp. lactis V9 and *Lactobacillus plantarum* P-8) in the treatment of hyperlipidemia, in combination with atorvastatin administration	Significant effect on levels of total cholesterol, triglycerides, and LDL cholesterol in the probiotic and control groups (*p* < 0.05). Gut microbial abundance in the probiotic group was significantly higher than that in the control group after 3 months (*p* < 0.05). At the phylum level, probiotics increased the abundance of Tenericutes and decreased that of Proteobacteria. At the genus level, probiotics increased the abundance of *Bifidobacterium*, *Lactobacillus*, and *Akkermansia* and decreased that of *Escherichia*, *Eggerthella*, and *Sutterella*.	Probiotics optimize the structure of the gut microbiota and decrease the amount of harmful bacteria in patients with hyperlipidemia.	High
Wang S et al., 2022 [116]	Multicenter randomized, placebo- controlled clinical trial	365 participants with T2D divided into 4 groups at 1:1:1:1 ratio	HbA1c, serum insulin, and C peptide were analyzed. Pyrosequencing of 16S rRNA genes to examine bacterial diversity.	The 4 groups were divided as follows: BBR (0.6 g per 6 pills, 2 v/day before a meal) + probiotics (4 g per 2 powder strips, 1 v/day at bedtime) (Prob + BBR); probiotics + placebo (Prob); BBR + placebo (BBR); or placebo + placebo (Plac).	12 weeks	To evaluate whether therapy combining probiotics and berberine, combined with an antidiabetic and hypolipidemic regimen, could reduce postprandial lipidemia in T2D	Prob + BBR was superior to BBR or Prob alone in improving postprandial col tot and LDL col levels with a decrease in multiple species of postprandial lipidomic metabolites	BBR and Prob may exert a synergistic hypolipidemic effect on PL, acting as a reservoir of intestinal lipids to achieve better control of lipidemia and CV risk in T2D.	High
Trotter RE et al., 2020 [117]	Randomized, double-blind, placebo-controlled study.	94 men and women aged 18–65 years with BMI between 20 and 34.9	Blood samples (100 mL) were collected from the antecubital vein in a lithium heparin tube and analyzed for total chol, HDL-c, triglycerides, non-HDL cholesterol, total/HDL cholesterol ratio, LDL-c and VLDL-c using a lipid panel reagent disc on the Piccolo Xpress Chemistry blood analyzer (Abaxis, Union City, CA, USA). Blood pressure and pulse wave analysis were also evaluated.	Subjects were given 15 mg/day supplementation of *B. subtilis* for 4 weeks.	4 weeks	To evaluate how Bacillus subtilis DE 111 supplementation may be helpful in improving the dyslipidemia picture and consequently CV risk factors	Supplementation with 15 mg daily resulted in a significant reduction in total cholesterol compared with baseline measurements, as well as LDL cholesterol. In addition, modest improvements in endothelial function and significant changes in several plasma lipids were observed	*B. subtilis* supplementation may be useful in improving risk factors associated with CVD.	High
Salamat et al., 2024 [118]	The double-blind randomized controlled trial	56 adult men aged 60 years or younger with dyslipidemia with TG of 200–400 mg/dL and LDL of 130–160 mg/dL randomly assigned to intervention and control groups	Blood and stool samples were collected at baseline and at the end of the study. Food intake, physical activity, anthropometric measures, serum IL-10 and fecal SCFAs were assessed before and after the intervention.	Subjects received synbiotic powder or placebo twice daily for 12 weeks.	12 weeks	To investigate the effects of multispecies synbiotic supplementation on serum interleukin 10 and fecal short-chain fatty acids (SCFAd) in patients with dyslipidemia	Serum IL-10 increased in the synbiotic group. Synbiotic supplementation increased the fecal concentrations of acetate, butyrate, propionate, and valerate.	A significant increase in fecal abundance of *Lactobacillus* and *Bifidobacterium* and serum HDL was observed in the synbiotic group.	High
**(D)**									
**Authors**	**Type of Studies**	**Number of Studies and Type of Study**	**Subject**	**End Point**		**Results**		**Conclusion**	**Strength of** **Evidence**
Sivamaruthi et al., 2019 [119]	Systematic review	/	Hypercholesterolemic subjects, healthy subjects, diabetes patients	To analyze the ability of probiotics (*Enterococcus faecium* CRL 183 and *Lactobacillus helveticus* 416) fermented with isoflavone-containing soy products to reduce cholesterol levels in hypercholesterolemic subjects		Supplementation of the soy product with 50 mg isoflavone daily for 42 days significantly improved total and LDL cholesterol while HDL levels remained unchanged. A 12-week supplementation with a mixture of *L. plantarum* strains (10 CFU/day) significantly increased HDL levels and reduced LDL, col tot, triglycerides, LDL/HDL ratio, and LDL levels. In dyslipidemic children, supplementation of a mixture of *Bifidobacterium* strains for three months improved the concentration of serum levels of col tot, HDL, TG and LDL. Consumption of a single probiotic strain (*E. faecium*) and selenium (50 μg) for one year did not alter HDL-C and TG levels, while it reduced TC and LDL-C in elderly people.		Probiotic consumption significantly improved the health status of hypercholesteremic patients by reducing LDL, total cholesterol and triglyceride levels and increasing HDL cholesterol.	High
Sivamaruthi et al., 2021 [120]	Systematic review	seventeen (*Lactobacillus* in nine studies, *Bifidobacterium* in eight studies and *Enterococcus* in two studies)	Hypercholesterolemic subjects	To analyze dietary interventions with probiotics in humans and their effects on cardiovascular risk factors and hypercholesterolemia		Supplementation of *B. longum* and red yeast rice in 33 patients with low CVD risk and no CVD risk led to a reduction in LDL-c levels; administration of *L. plantarum*, along with regular diet, to 23 people with hypercholesterolemia led to a reduction in LDL-c level. Administration of a soy product supplemented with isoflavones and fermented with *E. faecium* and *L. helveticus* to 17 patients led to a reduction in LDL-c level of up to 14.8 percent. Probiotic mixture consisting of *B. lactis* MB, *B. bifidum* and *B. longum* showed a significant reduction in TC, LDL-c and TG levels and an increase in HDL-c levels in dyslipidemic children. Probiotic mixture supplementation of three strains of *L. plantarum* significantly reduced TC (13.6%), LDL-c and LDL-c levels in hypercholesterolemic adults.		Probiotics have the propensity to become dietary supplements in moderate/severe hypercholesterolemic patients, which significantly reduces the CVD risk.	High
Gadelha CJMU et al., 2019 [121]	Systematic review	14 clinical studies	Subjects older than 18 years, predominantly with dyslipidemia	To examine the effects of probiotic supplementation on the prevention and treatment of dyslipidemia		Probiotic supplementation significantly reduced total cholesterol, LDL, and triglycerides and increased HDL values, especially when combined with other treatments (statins). The group with col tot > 200 mg/dL had the best response to probiotic treatment. Some benefits were also observed on anthropometric variables, glycemic control, oxidative stress, inflammatory markers, and immune system.		This study has shown that probiotic supplementation should be indicated as an additional treatment for lipid profile alterations.	High
Ettinger G et al., 2014 [122]	Meta-analysis	//	485 participants with “high”, “borderline”, and “normal” serum cholesterol levels	Examine the role of the microbiome in the prevention and treatment of cardiovascular disease		Several specific probiotic strains have been identified as effective in the management of hypercholesterolemia. The most effective probiotic strain clinically proven to reduce LDL-C levels by about 11.6 percent in hypercholesterolemic adults is *Lactobacillus reuteri* NCIMB 30242.		Probiotic consumption significantly reduced LDL-C and total cholesterol levels in all categories, compared with control.	High
Shimizu M et al., 2015 [123]	Meta-analysis	11 randomized controlled clinical trials describing data on differences before and after intervention in serum lipids, (col tot, LDL, HDL and TG)	The participants were healthy or hypercholesterolemic individuals of all ages (from infants to elderly people).	Demonstrate that probiotic supplementation may be useful in the primary prevention of hypercholesterolemia by leading to reduced risk factors for cardiovascular disease		Changes in col tot and LDL. Triglyceride and HDL levels did not differ significantly between the probiotic and control groups. Reductions in col tot and LDL levels with the probiotic intervention were greater in mildly hypercholesterolemic subjects. A subanalysis determined that the long-term (>4 weeks) probiotic intervention was statistically more effective in reducing TC and LDL-C than the short-term intervention, and high-dose probiotics more effectively reduced LDL-C levels than low-dose probiotics.		*Lactobacillus acidophilus* and Gaius reduced TC and LDL levels to a greater extent than the other bacterial strains.	High

**Table 7:** (**A**) Sarcopenia and microbiota: randomized and non-randomized controlled trials. (**B**) Probiotics and sarcopenia: randomized and non-randomized clinical trials. The upward arrow (↑) denotes an increase, whereas the downward arrow (↓) denotes a decrease.

**(A)**								
**Authors**	**Type of Studies**	**Population** **Characteristics**	**Type of** **Laboratory Evaluation**	**End Point**		**Results**	**Conclusion**	**Strength of** **Evidence**
Picca et al., 2020 [124]	Case–control study	N = 18 sarcopenic patients N = 17 healthy patients	Microbiota analysis on fecal samples	Microbiota composition		↑: *Bifidobacteriacee*, *Peptostreptococcacee* in sarcopenic group	Sarcopenic patients show modifications in microbiota composition	Medium
Wang et al., 2023 [125]	Case–control study	N = 50 sarcopenic patients N = 50 healthy controls	Microbiota analysis	Microbiota composition		↓: *Bifidobacterium longum*, *Prevotella coprii* in sarcopenic group	Sarcopenic patients show modifications in microbiota composition	Medium
Lee et al., 2020 [126]	Case–control study	N = 27 sarcopenic patients N = 33 healthy controls	Microbiota analysis	Microbiota composition		↓: *Prevotella copri* in sarcopenia group ↑: Anaerotruncus in sarcopenia group	Sarcopenic patients show modifications in microbiota composition	Medium
Liu et al., 2023 [127]	Case–control study	N = 141 sarcopenic patients N = 142 healthy controls	Microbiota analysis	Microbiota composition		↓: *Prevotella coprii*; BCAA metabolism in sarcopenia group ↑: *Bifidobacteria* in sarcopenia group	Sarcopenic patients show modifications in microbiota composition and less BCAA metabolism	Medium
Ticinesi et al., 2020 [128]	Case–control study	N = 5 sarcopenic patients N = 12 healthy controls	Shotgun sequencing on fecal samples	Microbiota composition		↓: SCFA production, *Faecalibacterium prausnizii*; *Roseburia inulinivorans*, *Alistipes shahii* in sarcopenia group	Sarcopenic patients show modifications in microbiota composition and less production of SCFA	Medium
Wang et al., 2022 [129]	Cross-sectional study	N = 141 sarcopenic patients N = 1276 healthy controls	Shotgun sequencing on fecal samples	Microbiota composition		↓: beta-diversity in sarcopenia group ↑: Clostridium	Sarcopenic patients show less beta-diversity in microbiota composition	Medium
Kang et al., 2021 [130]	Longitudinal study	N = 27 sarcopenic patients N = 60 healthy controls	Sequencing on fecal samples	Changes in microbiota composition		↓: alpha-diversity, beta-diversity, SCFA production, Firmicutes in sarcopenia patients ↑: *Porphyromondanaceae*, *Lactobacillaceae* in sarcopenia patients	Sarcopenic patients show modifications in microbiota composition	Medium
**(B)**								
**Authors**	**Type of Studies**	**Population** **Characteristics**	**Type of** **Intervention**	**Duration**	**End Point**	**Results**	**Conclusion**	**Strength of** **Evidence**
Lee et al., 2018 [126]	Randomized, double-blind study	Elderly patients with frailty	Administration of lactobacillus plantarum TWK10	>6 weeks	Muscle mass (kg) HGS (kg)	↑: muscle strength, endurance, HGS, gait speed ↓: sarcopenia, muscle weakness	Treatment with probiotics ameliorates muscle function	High
Chaiyasut et al., 2022 [131]	Randomized, double-blind study	Healthy older adults	Administration mixture of probiotics (2.0 × 10^10^ CFU of *L. paracasei* HII01; 2.0 × 10^10^ CFU of *B. breve*; 1.0 × 10^10^ CFU of *B. longum*) (Lactomason Co., Ltd., Jinju-si, Republic of Korea)	12 weeks	Muscle mass (%)	↑: HDL-c; muscle % ↓: VAT %, body fat %	Treatment with probiotics ameliorates body composition and lipid profile	High
Tominaga et al., 2021 [132]	Not controlled, experimental study	Patients with frailty	Administration of 20 g of prebiotic 1-kestose daily	8 weeks	Fecal microbiota composition	↑: increase in *Bifidobacterium longum* population	Treatment with probiotics modifies microbiota composition	Medium
Karim et al., 2022 [133]	Randomized, double-blind study	Patients with chronic heart failure and sarcopenia	Administration of probiotic with 112 billion UFC	12 weeks	ASM (kg) HGS (kg)	↑: improvement in HGS, SPPB and gait speed in probiotic group	Treatment with probiotics ameliorates muscle function	High
Karim et al., 2022 [134]	Randomized double-blind study	Patients with COPD and sarcopenia	Administration of probiotic with 112 billion UFC	16 weeks	ASM (kg) HGS (kg)	↑: improvement in handgrip; SPPB, gait speed in probiotic group	Treatment with probiotics ameliorates muscle function	High
Rondanelli et al., 2022 [135]	Randomized double-blind study	Patients with sarcopenia	Administration of novel food composed of Leucine, Omega-3 fatty acids and probiotic *Lactobacillus paracasei* PS23	2 months	ASM (kg) HGS (kg)	↑: improvement in ALM and plasma amino acids in probiotic group ↓: decrease in visceral adiposity in probiotic group	Treatment with probiotics ameliorates body composition and increases plasma amino acids	High
Ford et al., 2020 [136]	Placebo-controlled, double-blind, cross-sectional study	Elderly woman	1.54 × 10^9^ *Bifidobacterium bifidum* HA-132, 4.62 × 10^9^ *Bifidobacterium* *Breve HA*-129, 4.62 × 10^9^ *Bifidobacterium longum* HA-135, 4.62 × 10^9^ *Lactobacillus acidophilus* HA-122, and 4.62 × 10^9^ *Lactobacillus plantarum HA-119*	2-week periods with 2-week diet washout	ASM (kg)	↑: increase in fat-free mass	Treatment with probiotics ameliorates body composition	High
Qaisar et al., 2024 [137]	Randomized, controlled study	Elderly man with sarcopenia	*B. longum* DSM 24736, *B. breve* DSM 24732, DSM 24737, *Streptococcus thermophilus* DSM 24731, e lactobacilli (DSM 24735, DSM 24730, DSM 24733, *L. delbrueckii* subsp. *bulgaricus* DSM 24734	16 weeks	Sar-QoL scores, fecal zonulin; gait speed (%), HGS (%)	↑: gait speed, HGS	Treatment with probiotics ameliorates muscle function	High

**Table 8:** (**A**) NAFLD and microbiota: randomized and non-randomized controlled trials. (**B**) NAFLD and microbiota: systematic reviews and meta-analyses. (**C**) Probiotics and NAFLD: randomized and non-randomized clinical trials. (**D**) Probiotics and NAFLD: systematic reviews and meta-analyses.

**(A)**									
**Authors**	**Type of Studies**	**Population** **Characteristics**	**Type of Laboratory Evaluation**	**Type of** **Intervention** **(If Applicable)**	**Duration**	**End Point**	**Results**	**Conclusion**	**Strength of** **Evidence**
Jumpertz, R. et al., 2011 [138]	Observational study	12 lean and 9 obese individuals	Pyrosequencing bacterial 16S ribosomal RNA (rRNA) genes present in the feces of participants and measuring ingested and stool calories with the use of bomb calorimetry	-	-	To evaluate changes in gut microbiota during diets that varied in caloric content (2400 compared with 3400 kcal/d). To study how gut bacterial community structure is affected by altering the nutrient load in lean and obese individuals and whether their microbiota are correlated with the efficiency of dietary energy harvest.	The alteration of the nutrient load induced rapid changes in the gut microbiota. These changes were directly correlated with stool energy loss in lean individuals such that a 20% increase in Firmicutes and a corresponding decrease in Bacteroidetes were associated with an increased energy harvest of ≈150 kcal. A high degree of overfeeding in lean individuals was accompanied by a greater fractional decrease in stool energy loss.	These results show that the nutrient load is a key variable that can influence the gut (fecal) bacterial community structure over short time scales. Furthermore, the observed associations between gut microbes and nutrient absorption indicate a possible role of the human gut microbiota in the regulation of the nutrient harvest.	Low
Mouzaki, M. et al., 2013 [139]	Prospective, cross-sectional study	50 subjects included: 11 with simple steatosis (SS), 22 with non-alcoholic steatohepatitis (NASH), and 17 living liver donors as healthy controls (HCs)	One stool sample was collected from each participant. Quantitative real-time polymerase chain reaction was used to measure total bacterial counts, Bacteroides/Prevotella (herein referred to as Bacteroidetes), *Clostridium leptum*, *C. coccoides*, bifidobacteria, *Escherichia coli* and Archaea in stool. Clinical and laboratory data, food records, and activity logs were collected.	-	-	To identify differences in intestinal microbiota between adults with biopsy-proven NAFLD (SS or NASH) and HCs	Patients with NASH had a lower percentage of Bacteroidetes (Bacteroidetes to total bacteria counts) compared to both SS and HC (*p* = 0.006) and higher fecal *C. coccoides* compared to those with SS (*p* = 0.04). There were no differences in the remaining microorganisms. As body mass index (BMI) and dietary fat intake differed between the groups (*p* < 0.05), we performed linear regression adjusting for these variables. The difference in *C. coccoides* was no longer significant after adjusting for BMI and fat intake. However, there continued to be a significant association between the presence of NASH and lower percentages of Bacteroidetes even after adjusting for these variables (*p* = 0.002; 95% confidence interval = −0.06 to −0.02).	There is an inverse and diet-/BMI-independent association between the presence of NASH and percentage Bacteroidetes in the stool, suggesting that the IM may play a role in the development of NAFLD.	Low
Zhang C. et al., 2018 [140]	Observational study	15 healthy volunteers, who normally consume an omnivorous diet (study group), 7 healthy omnivorous volunteers (control group 1) and 7 healthy long-term vegetarians (lacto-ovo-vegetarian diet, control group 2)	Blood and fecal samples were collected and weight was measured days 0 and 91 for all individuals to determine the composition of their microbiota.	Subjects in study group changed to a lacto-ovo-vegetarian diet for 3 months. The participants had not taken antibiotics 3 months before the study nor during the study period. Blood and fecal samples were collected and weight was measured days 0 and 91 for all individuals.	3 months	To investigate the effect of a 3-month lacto-ovo-vegetarian diet on the diversity of gut microbiota and the immune system in healthy omnivorous volunteers.	The short-term vegetarian diet did not have any major effect on the diversity of the immune system and the overall composition of the metagenome. The prevalence of bacterial genera/species with known beneficial effects on the intestine, including butyrate producers and probiotic species, and the balance of autoimmune-related variable genes/families were, however, altered in the short-term vegetarians. A number of bacterial species that are associated with the expression level of IgA, a key immunoglobulin class that protects the gastrointestinal mucosal system, were also identified. Furthermore, a lower diversity of T-cell repertoire and expression level of IgE, as well as a reduced abundance of inflammation-related genes in the gut microbiota, were potentially associated with a control group with long-term vegetarians.	Thus, the composition and duration of the diet may have an impact on the balance of pro-/anti-inflammatory factors in the gut microbiota and immune system.	Low
Miele, L. et al., 2009 [141]	Observational study	35 consecutive patients with biopsy-proven NAFLD, 27 with untreated celiac disease (as a model of intestinal hyperpermeability) and 24 healthy volunteers	Assessment of the presence of SIBO by glucose breath testing (GBT), intestinal permeability by means of urinary excretion of (51)Cr-ethylene diamine tetraacetate ((51)Cr-EDTA) test, and the integrity of tight junctions within the gut by immunohistochemical analysis of zona occludens-1 (ZO-1) expression in duodenal biopsy specimens	-	-	To investigate intestinal permeability in patients with NAFLD and evaluated the correlations between this phenomenon and the stage of the disease, the integrity of tight junctions within the small intestine, and prevalence of small intestinal bacterial overgrowth (SIBO)	Patients with NAFLD had significantly increased gut permeability (compared with healthy subjects; *p* < 0.001) and a higher prevalence of SIBO, although both were lower than in the untreated celiac patients. In patients with NAFLD, both gut permeability and the prevalence of SIBO correlated with the severity of steatosis but not with presence of NASH.	NAFLD in humans is associated with increased gut permeability and this abnormality is related to the increased prevalence of SIBO in these patients. The increased permeability appears to be caused by disruption of intercellular tight junctions in the intestine, and it may play an important role in the pathogenesis of hepatic fat deposition.	Low
Fava, F. et al., 2013 [142]	Randomized, controlled, single-blind, parallel design	88 subjects at increased MetS risk	High monounsaturated fat (MUFA)/high glycemic index (GI) (HM/HGI); high MUFA/low GI (HM/LGI); high carbohydrate (CHO)/high GI (HC/HGI); and high CHO/low GI (HC/LGI). Dietary intakes, MetS biomarkers, fecal bacteriology and SCFA concentrations were monitored.	Subjects were fed a high-saturated-fat diet (HS) for 4 weeks (baseline), then randomized onto one of the five experimental diets for 24 weeks: HS; high monounsaturated fat (MUFA)/high glycemic index (GI) (HM/HGI); high MUFA/low GI (HM/LGI); high carbohydrate (CHO)/high GI (HC/HGI); and high CHO/low GI (HC/LGI).	28 weeks	To determine the effect of the amount and type of dietary fat and carbohydrate on fecal bacteria and short-chain fatty acid (SCFA) concentrations in people ‘at risk’ of MetS.	High-MUFA diets did not affect individual bacterial population numbers but reduced total bacteria and plasma total and LDL cholesterol. The low-fat, HC diets increased fecal *Bifidobacterium* (*p* = 0.005, for HC/HGI; *p* = 0.052, for HC/LGI) and reduced fasting glucose and cholesterol compared to baseline. HC/HGI also increased fecal Bacteroides (*p* = 0.038), whereas HC/LGI and HS increased *Faecalibacterium prausnitzii* (*p* = 0.022 for HC/HGI and *p* = 0.018, for HS). Importantly, changes in fecal Bacteroides numbers correlated inversely with body weight (r = −0.64). A total bacterial reduction was observed for high-fat diets HM/HGI and HM/LGI (*p* = 0.023 and *p* = 0.005, respectively) and HS increased fecal SCFA concentrations (*p* < 0.01).	This study provides new evidence from a large-scale dietary intervention study that HC diets, irrespective of GI, can modulate human fecal saccharolytic bacteria, including Bacteroides and bifidobacteria. Conversely, high-fat diets reduced bacterial numbers, and in the HS diet, there was increased excretion of SCFAs, which may suggest a compensatory mechanism to eliminate excess dietary energy.	High
Zelber-Sagi, S. et al., 2018 [143]	Cross-sectional study	789 individuals, 40–70 years old, who underwent screening colonoscopy between 2013 and 2015 in a single center in Israel	NAFLD and IR were evaluated by ultrasonography and homeostasis model assessment.			To test the association of meat type and cooking method with NAFLD and insulin resistance (IR)	High consumption of total, red and/or processed meat was independently associated with higher odds of NAFLD and IR, respectively, when adjusted for body mass index, physical activity, smoking, and alcohol, energy, saturated fat and cholesterol intake. High intake of meat cooked using unhealthy methods and heterocyclic amines (formed by cooking meat at high temperatures for a long duration) were independently associated with higher odds of IR.	High consumption of red and/or processed meat is associated with both NAFLD and IR. High HCA intake is associated with IR. If confirmed in prospective studies, limiting the consumption of unhealthy meat types and improving preparation methods may be considered as part of NAFLD lifestyle treatment.	Medium
Wehmeyer, M.H et al., 2016 [144]	Observational study	55 consecutive patients diagnosed with NAFLD compared to an age- and gender-matched cohort of 88 healthy individuals by univariate analysis	Biochemical data: AST, ALT, GGT, gluco-lipid pattern. Hepatic fibrosis was evaluated by a transient elastography of the liver.	The efficacy of the dietary intervention was assessed in a subgroup of 24 NAFLD patients 6 months after receiving dietary advice.		To assess the dietary patterns associated with non-alcoholic fatty liver disease (NAFLD) and the efficacy of dietary interventions in a real-life setting	NAFLD patients consumed more calories per day as compared with healthy controls (*p* < 0.001). The absolute amounts of most nutritional components ingested by NAFLD patients were higher than those of the controls. However, there were no significant differences with regard to the relative consumption of carbohydrates (*p* = 0.359), fat (*p* = 0.416), and fructose (*p* = 0.353) per 1000 kcal energy intake. NAFLD patients displayed a higher intake of glucose/1000 kcal (*p* = 0.041) and protein/1000 kcal (*p* = 0.009) but a lower intake of fibers/1000 kcal (*p* < 0.001) and mineral nutrients/1000 kcal (*p* = 0.001) than healthy controls. In the longitudinal analysis, patients significantly reduced their caloric intake, and their ALT levels improved 6 months after the dietary counseling (*p* < 0.001).	The study demonstrates that dietary patterns of patients with NAFLD display great variability and little disease specificity, while the most distinctive feature compared with healthy controls was higher energy intake in NAFLD patients.	Low
Cotillard, A. et al., 2013 [111]	Clinical trial	38 obese and 11 overweight individuals.	Anthropometric markers. Biochemical parameters: plasma glucose and lipid homeostasis, inflammatory markers (PCR, IL-6). Subcutaneous abdominal adipose tissue samples were obtained at all time points by needle biopsy from the periumbilical area under local anesthesia (1% xylocaine) to measure the adipocyte diameter and for immunohistochemical studies.	Diet-induced weight-loss and weight-stabilization interventions	12 weeks	To investigate the temporal relationships between food intake, gut microbiota and metabolic and inflammatory phenotypes	Individuals with reduced microbial gene richness (40%) present more pronounced dys-metabolism and low-grade inflammation.	Dietary intervention improves low gene richness and clinical phenotypes, but seems to be less efficient for inflammation variables in individuals with lower gene richness. Low gene richness may therefore have predictive potential for the efficacy of intervention.	Medium
Parker, H.M et al., 2019 [145]	Double-blind randomized controlled trial	Fifty apparently healthy overweight men (BMI 25.0–29.9 kg/m^2^; waist > 94 cm) randomly allocated to consume fish oil or placebo (olive oil capsules).	Anthropometric parameters: standing height, weight, waist circumference. Body composition measured using BIA. Biochemical parameters: serum aminotransferases (ALT, AST, GGT) and triglycerides (TG), omega-3 index testing. Dietary and physical activity habits. MRI and 1H-MRS methods for quantifying abdominal visceral (VAT) and subcutaneous (SAT) adipose tissue, liver fat (intrahepatic lipid; IHL) concentration and composition.	Treatment group: total daily dose: 1728 mg marine triglycerides, of which 588 mg EPA and 412 mg DHA, combined with 200 mg antioxidant (coenzyme Q10). Placebo group: olive oil capsules daily for 12 weeks	12 weeks	To evaluate effect of fish oil supplementation on liver fat	No significant time or group × time effect for fish oil versus placebo for liver fat, liver enzymes, anthropometry, or body composition including VAT, with similar finding for sub-analysis of participants with NAFLD.	Omega-3 PUFA did not appear to be an effective agent for reducing liver fat in overweight men.	High
Šmíd V et al., 2022 [146]	Double-blind placebo-controlled trial	Sixty patients with metabolic syndrome and NAFLD were randomized in a double-blind placebo-controlled trial	Biochemical parameters: AST, ALT, GGT, gluco-lipid assessment. Liver stiffness was evaluated by ultrasonography. The 1H MRS was used for visceral and liver fat determination. Plasma lipidomics was determined using UPLC-HR-MS. Genomic DNA was isolated from peripheral blood white cells by the standard salting-out procedure. The specific variants of the PNPLA3 (rs738409 and rs738408), TM6SF2 (rs58542926), and MBOAT7 (rs641738) genes were typed by PCR.	Intervention group assumed 3.6 g/day n-3-PUFA for one year vs. placebo.	1 year	To assess the effects of n-3-PUFA administration on lipid metabolism and the progression of NAFLD in patients with metabolic syndrome	After 12 months of n-3-PUFA administration, a significant decrease in serum GGT activity was recorded compared with the placebo group (2.03 ± 2.8 vs. 1.43 ± 1.6; *p* < 0.05). Although no significant changes in anthropometric parameters were recorded, a significant correlation between the reduction in liver fat after 12 months of treatment and weight reduction was observed; furthermore, this effect was clearly potentiated by n-3-PUFA treatment (*p* < 0.005). In addition, n-3-PUFA treatment resulted in substantial changes in the plasma lipidome, with n-3-PUFA-enriched triacylglycerols and phospholipids being the most expressed lipid signatures.	Twelve months of n-3-PUFA treatment of patients with NAFLD patients was associated with a significant decrease in GGT activity, the liver fat reduction in those who reduced their weight, and beneficial changes in the plasma lipid profile.	High
**(B)**									
**Authors**	**Type of Studies**	**Number of Studies and Type of Study**	**Subject**	**End Point**		**Results**	**Conclusion**		**Strength of** **Evidence**
Maestri M. et al., 2023 [147]	Narrative Review	-	-	To investigate the impact of the gut microbiota on the molecular mechanisms underlying NAFLD; to understand how current therapeutic approaches used to treat NAFLD/MAFLD and its associated comorbidities may influence the natural history of the disease through gut microbiota modulation. Finally, to point the view to what may become future therapeutic weapons in NAFLD/MAFLD, acting on the gut microbiota.		The gut–liver axis has a strong impact in the promotion of NAFLD and in the progression of the wide spectrum of its manifestations. Western diet negatively affects intestinal permeability and the gut microbiota composition and function, selecting pathobionts, whereas Mediterranean diet fosters health-promoting bacteria, with a favorable impact on lipid and glucose metabolism and liver inflammation. Drugs for the treatment of type 2 diabetes mellitus (T2DM), such as metformin, glucagon-like peptide-1 (GLP-1) agonists, and sodium-glucose cotransporter (SGLT) inhibitors, are not only effective in the regulation of glucose homeostasis, but also in the reduction in liver fat content and inflammation, and they are associated with a shift in the gut microbiota composition towards a healthy phenotype. Even bariatric surgery significantly changes the gut microbiota, mostly due to the modification of the gastrointestinal anatomy, with a parallel improvement in histological features of NAFLD.	Mediterranean diet, drugs for the treatment of type 2 diabetes mellitus, and bariatric surgery have positive impacts on gut microbiota and NAFLD. Other options with promising effects in reprogramming the gut–liver axis, such as fecal microbial transplantation (FMT) and next-generation probiotics, deserve further investigation for future inclusion in the therapeutic armamentarium of NAFLD.		Medium
Aron-Wisnewsky J. et al., 2013 [148]	Narrative review	-	-	To summarizes what is currently known of microbiota composition in obesity and the physiopathogenesis of NAFLD in that context		Gut microflora may stimulate hepatic fat deposition and promote NASH though several mechanisms: it promotes obesity by improving energy yield from food, it regulates gut permeability, low-grade inflammation and immune balance, it modulates dietary choline metabolism, it regulates bile acid metabolism, and finally it increases endogenous ethanol production by bacteria.	The gut microbiota is involved in gut permeability, low-grade inflammation and immune balance; it modulates dietary choline metabolism, regulates bile acid metabolism and produces endogenous ethanol. All of these factors are molecular mechanisms by which the microbiota can induce NAFLD or its progression toward overt non-alcoholic steatohepatitis.		Medium
Wieland, A et al., 2015 [149]	Systematic review	Nine studies (five human and four animal models)	226 humans	To perform a comprehensive review of the medical literature investigating associations between intestinal dysbiosis and NAFLD, with a particular emphasis on studies that characterize the microbiome in NAFLD.		Because of the anatomical links between the intestines and the liver, dysbiosis may also disrupt hepatic function and thereby contribute to the pathogenesis of non-alcoholic fatty liver disease (NAFLD) through these mechanisms: − Facilitation of host energy harvest and utilization by the microbiota; − Altered dietary choline metabolism by the microbiota; − Impact of the microbiota on bile acid metabolism; − Gut permeability alterations.	Investigations in humans and animals demonstrate associations between intestinal dysbiosis and NAFLD; however, causality has not been proven and mechanistic links require further delineation.		High
Roychowdhury, S. et al., 2018 [150]	Narrative review	-	-	To review the progression of NAFLD, discussing the mechanistic modes of hepatocyte injury and the potential role for manipulation of the gut microbiome as a therapeutic strategy in the prevention and treatment of NAFLD		There is no concrete evidence to support probiotics as monotherapy for NAFLD, which has a multi-hit pathophysiology. Nonetheless, manipulation of the gut microbiome using probiotics may be used as combination therapy, in addition to lifestyle interventions and other available natural or pharmacologic options, especially in patients who are struggling with compliance.	Only diet and lifestyle change have been demonstrated to improve obesity and NAFLD, but patient compliance is problematic. As more is being discovered about the gut microbiome and its role in obesity and liver disease, future directions in targeting the gut microbiome as a therapeutic option for NAFLD is warranted. However, several questions remain unanswered, such as the actual mechanism of action of probiotics in NAFLD, the differences in efficacy between children and adults with NAFLD, the comparison of efficacy of available probiotics, the specific targets of each probiotic, and long-term outcomes.		Medium
Aron-Wisnewsky J. et al., 2020 [148]	Narrative review	-	-	To provide a broad insight into microbiome signatures for human NAFLD and to explore issues with disentangling these signatures from underlying metabolic disorders.		Whilst animal studies have demonstrated a potential causal role of gut microbiota in non-alcoholic fatty liver disease (NAFLD), human studies have only just started to describe microbiome signatures in NAFLD. Proteobacteria are consistently enriched in steatosis and non-alcoholic steatohepatitis. Bacterial signatures (*Clostridium* and *Lactobacillus*) overlap between NAFLD and metabolic diseases (type 2 diabetes mellitus).	Discrepant microbiome signatures across studies could be linked to the heterogeneity of geographical regions, ethnicity, population characteristics, microbiome sequencing tools, NAFLD diagnostic tools, disease spectrum, drug consumption and circadian rhythm.		Medium
Albillos A. et al., 2020 [151]	Narrative review	-	-	To find out the pathophysiological basis for therapy of liver diseases based on the gut–liver axis		Growing evidence indicates the pathogenetic role of microbe-derived metabolites, such as trimethylamine, secondary bile acids, short-chain fatty acids and ethanol, in the pathogenesis of non-alcoholic fatty liver disease. The identification of the elements of the gut–liver axis primarily damaged in each chronic liver disease offers possibilities for intervention.	Beyond antibiotics, upcoming therapies centered on the gut include new generations of probiotics, bacterial metabolites (postbiotics), fecal microbial transplantation, and carbon nanoparticles. FXR agonists target both the gut and the liver and are currently being tested in different liver diseases. Finally, synthetic biotic medicines, phages that target specific bacteria, and therapies that create physical barriers between the gut and the liver offer new therapeutic approaches.		Medium
Wu L. et al., 2021 [152]	Narrative review	-	-	To integrate related articles on gut microbiota, PPARs and NAFLD, and present a balanced overview on how the microbiota can possibly influence the development of NAFLD through PPARs		Clinical studies have shown that gut microbiome signatures in NAFLD may serve as diagnostic biomarkers for liver disease. In connection with this, PPARs are transcription factors involved in the regulation of lipid metabolism, energy balance, and inflammation, thus playing a decisive role in various metabolic diseases.	Peroxisome proliferator-activated receptors (PPARs) are members of the nuclear receptor superfamily and can regulate multiple pathways involved in metabolism, and serve as effective targets for the treatment of many types of metabolic syndromes, including NAFLD.		Medium
Arslan, N. et al., 2014 [153]	Narrative review	-	-	To review the relationship between intestinal microbial changes and obesity and its complications, including insulin resistance and NAFLD		Given that the gut and liver are connected by the portal venous system, it makes the liver more vulnerable to translocation of bacteria, bacterial products, endotoxins or secreted cytokines. Altered intestinal microbiota (dysbiosis) may stimulate hepatic fat deposition through several mechanisms: regulation of gut permeability, increasing low-grade inflammation, modulation of dietary choline metabolism, regulation of bile acid metabolism and producing endogenous ethanol.	High-energy diets alter intestinal microbiota and induce gut dysfunction, which subsequently result in visceral fat inflammation and systemic metabolic dysregulation. An obesogenic microbiota can alternate liver function by stimulating hepatic triglycerides and by modulating systemic lipid metabolism, which indirectly impact the storage of fatty acids in the liver. Several studies suggested that intestinal microbiota might also play an important part in the progression of NAFLD to NASH. Modulation of gut microbiota by diet modifications or by using probiotics, prebiotics and synbiotics as a treatment for obesity and fatty liver disease might be a topic of further investigations.		Medium
Houghton D. et al., 2016 [154]	Narrative review	-	-	To review the effects of lifestyle interventions (diet and physical activity/exercise) on gut microbiota and how this impacts NAFLD prognosis		The Western diet (high in fat and carbohydrates) is associated with an altered gut microbiota and increased risk of developing obesity and NAFLD. Fructans are the most extensively studied prebiotics and have been linked with modulation of the gut microbiota, resulting in positive health benefits. Probiotics have been suggested as a potential treatment for patients with NAFLD, due to their apparent ability to modulate the gut microbiota and impact metabolic control, inflammation, lipid profile and intestinal permeability. Exercise does appear to be able to modulate the gut microbiota and reduce the risk of NAFLD with different potential mechanisms: increased butyrate production, which is linked with colonic epithelial cell proliferation and modulation of mucosal immunity and exclusion of pathogens; increased primary bile acid secretion and cholesterol turnover; growth of beneficial bacteria; increased core body temperature; and reduced blood flow to the GI system, reducing gut transit time and substrate delivery to the microbiota.	This review reveals that diet, pre/probiotics, and exercise play a significant role in the function and diversity of the gut microflora. To date, studies have predominantly focused on preclinical models, which have limitations in the transferability of their data to humans. Although much is known, there are significant questions about how lifestyle therapies may influence the gut microbiota as a therapeutic target for NAFLD care.		Medium
**(C)**									
**Authors**	**Type of Studies**	**Population** **Characteristics**	**Type of Laboratory Evaluation**	**Type of** **Intervention**	**Duration**	**End Point**	**Results**	**Conclusion**	**Strength of** **Evidence**
Chang HC et al., 2013 [155]	Double-blinded, randomized clinical trial	40 subjects with BMI ≥27 aged 18–65 randomly divided into a control (n = 18) and an oat-treated (n = 16) group	Serum parameters (glucose, TG, cholesterol, LDL-C, HDL-C, FFA, AST, ALT, GGT, creatinine, blood urea nitrogen, albumin, uric acid,), BMI, waist-to-hip ratio, body fat	Oat group taking beta-glucan-containing oat cereal and the other a placebo (with a similar composition but without beta-glucan). One cereal pack (37.5 g) was prescribed to be mixed with 250 mL hot water and replace some staple foods in meals twice daily for 12 weeks.	12 weeks	To verify if oat, rich in beta-glucan, had a metabolic-regulating and liver-protecting effect in humans	Consumption of oat reduced body weight, BMI, body fat and the waist-to-hip ratio. Profiles of hepatic function, including AST, but especially ALT, were useful resources to help in the evaluation of the liver, since both showed decrements in patients with oat consumption. Nevertheless, anatomic changes were still not observed by ultrasonic image analysis. Ingestion of oat was well tolerated and there was no adverse effect during the trial.	Consumption of oat reduced obesity, abdominal fat, and improved lipid profiles and liver functions. Taken as a daily supplement, oat could act as an adjuvant therapy for metabolic disorders.	High
**(D)**									
**Authors**	**Type of Studies**	**Number of Studies and Type of Study**	**Subject**	**End Point**		**Results**	**Conclusion**		**Strength of** **Evidence**
Perumpail, B.J. et al., 2019 [156]	Narrative review	-	-	To deepen probiotic supplementation as a potential treatment method for NAFLD due to its ability to retard and/or reverse dysbiosis and restore normal gut flora		All studies reviewed indicate that probiotics had a beneficial effect in patients with NAFLD and its subset NASH. Results varied between studies, but there was evidence demonstrating improvement in liver enzymes, hepatic inflammation, hepatic steatosis, and hepatic fibrosis. No major adverse effects were noted. Currently, there are no guidelines addressing the use of probiotics in the setting of NAFLD.	Probiotics appear to be a promising option in the treatment of NAFLD. Future research is necessary to assess the efficacy of probiotics in patients with NAFLD.		Medium
Xiao, M.-W et al., 2020 [157]	Systematic review and meta-analysis	28 RCTs	1555 proven NAFLD patients	To verify if probiotics can be considered as a potential therapy for non-alcoholic fatty liver disease (NAFLD)		The use of probiotics was evaluated from 4 to 28 weeks. Overall, probiotic therapy had beneficial effects on body mass index (WMD: −1.46, 95% CI: [−2.44, −0.48]), alanine aminotransferase (WMD: −13.40, 95% CI: [−17.03, −9.77]), aspartate transaminase (WMD: −13.54, 95% CI: [−17.86, −9.22]), gamma-glutamyl transpeptidase (WMD: −9.88, 95% CI: [−17.77, −1.99]), insulin (WMD: −1.32, 95% CI: [−2.43, −0.21]), homeostasis model assessment insulin resistance (WMD: −0.42, 95% CI: [−0.73, −0.12]), and total cholesterol (WMD: −15.38, 95% CI: [−26.50, −4.25]), but not fasting blood sugar, lipid profiles, or tumor necrosis factor-alpha.	The systematic review and meta-analysis support that probiotics are superior to placebo in NAFLD patients and could be utilized as a common complementary therapeutic approach.		High
Loman, B.R et al., 2018 [158]	Systematic review and meta-analysis	25 RCTs	1309 patients	To systemically review and quantitatively synthesize evidence on prebiotic, probiotic, and synbiotic therapies for patients with NAFLD in randomized controlled trials		Meta-analysis indicated that microbial therapies significantly reduced BMI (−0.37 kg/m2; 95% confidence interval [CI], −0.46 to −0.28; *p* < 0.001), hepatic enzymes (ALT, −6.9 U/L [95%CI, −9.4 to −4.3]; AST, −4.6 U/L [95%CI, −6.6 to −2.7]; γ-GT, −7.9 U/L [95%CI, −11.4 to −4.4]; *p* < 0.001), serum cholesterol (−10.1 mg/dL 95%CI, −13.6 to −6.6; *p* < 0.001), LDL-c (−4.5 mg/dL; 95%CI, −8.9 to −0.17; *p* < 0.001), and TAG (−10.1 mg/dL; 95%CI, −18.0 to −2.3; *p* < 0.001), but not inflammation (TNF-α, −2.0 ng/mL; [95%CI, −4.7 to 0.61]; CRP, −0.74 mg/L [95%CI, −1.9 to 0.37]). Subgroup analysis by treatment category indicated similar effects of prebiotics and probiotics on BMI and liver enzymes but not total cholesterol, HDL-c, and LDL-c.	This meta-analysis supports the potential use of microbial therapies in the treatment of NAFLD and sheds light on their potential mode of action. Further research into these treatments should consider the limitations of biomarkers currently used for the diagnosis and progression of NAFLD, in addition to the inherent challenges of personalized microbial-based therapies.		High
Khan, M.Y. et al., 2019 [159]	Systematic review and meta-analysis	12 randomized controlled trials	624 subjects	To study the effect of probiotics/synbiotics on various laboratory and radiographic parameters in NAFLD management		The intervention arm, which comprising the probiotic and/or the synbiotic arm, showed a significant improvement in alanine aminotransferase levels (MD = −13.93, confidence interval (CI) = −20.20 to −7.66, *p* value of less than 0.0001, I = 92%) and aspartate aminotransferase levels (MD = −11.45, CI = −15.15 to −7.74, *p* value of less than 0.00001, I = 91%). There was a reduction in high-sensitivity C-reactive protein levels in the intervention arm (SMD = −0.68, CI = −1.10 to −0.26, *p* value of 0.001, I = 0%). The liver fibrosis score improved in the intervention arm (MD = −0.71, CI = −0.81 to −0.61, *p* value less than 0.00001, I = 0%).	Probiotic/synbiotic use improves aminotransaminase levels and reduces pro-inflammatory marker high-sensitivity C-reactive protein and liver fibrosis in NAFLD patients.		High
Sharpton, S.R. et al., 2019 [160]	Systematic review and meta-analysis	21 RCTs	1252 subjects	To evaluate the most current evidence for liver-specific and metabolic effects of microbiome-targeted therapies (MTTs) in persons with NAFLD		Probiotics/synbiotics were associated with a significant reduction in alanine aminotransferase activity [ALT, weighted mean difference (WMD): −11.23 IU/L; 95% CI: −15.02, −7.44 IU/L] and liver stiffness measurement (LSM) by elastography (reflecting inflammation and fibrosis) (WMD: −0.70 kPa; 95% CI: −1.00, −0.40 kPa), although analyses showed heterogeneity (I^2^ = 90.6% and I^2^ = 93.4%, respectively). Probiotics/synbiotics were also associated with increased odds of improvement in hepatic steatosis, as graded by ultrasound (OR: 2.40; 95% CI: 1.50, 3.84; I^2^ = 22.4%). No RCTs examined sequential liver biopsy findings. Probiotics (WMD: −1.84; 95% CI: −3.30, −0.38; I^2^ = 23.6%), but not synbiotics (WMD: −0.85; 95% CI: −2.17, 0.47; I^2^ = 96.6%), were associated with a significant reduction in body mass index.	The use of probiotics/synbiotics was associated with improvement in liver-specific markers of hepatic inflammation, LSM, and steatosis in persons with NAFLD. Although promising, given the heterogeneity in pooled analyses, additional well-designed RCTs are needed to define the efficacy of probiotics/synbiotics for treatment of NAFLD.		High

## Data Availability

The data presented in this study are available in the article.
